# Transcription factor NF-Y complex interacts with chromatin remodeling complexes SWI/SNF and RSC to coordinately regulate gene expression

**DOI:** 10.1093/nar/gkag543

**Published:** 2026-05-30

**Authors:** Kexuan Ma, Mengxue Li, Haiyi Yuan, Jian Zhao, Yuqi Qin

**Affiliations:** National Glycoengineering Research Center, Shandong University, Qingdao, 266237, China; State Key Laboratory of Microbial Technology, Shandong University, Qingdao, 266237, China; National Glycoengineering Research Center, Shandong University, Qingdao, 266237, China; National Glycoengineering Research Center, Shandong University, Qingdao, 266237, China; State Key Laboratory of Microbial Technology, Shandong University, Qingdao, 266237, China; National Glycoengineering Research Center, Shandong University, Qingdao, 266237, China; State Key Laboratory of Microbial Technology, Shandong University, Qingdao, 266237, China

## Abstract

The NF-Y (HAP2/3/5 or CCAAT-binding factor) complex is an evolutionarily conserved heterotrimeric transcription factor (TF) that binds the CCAAT box in ∼30% of eukaryotic promoters. While NF-Y alters chromatin structure, its mechanisms remain enigmatic. In *Penicillium oxalicum* and *Trichoderma reesei*, near-complete chromatin remodeling complexes (CRCs) SWI/SNF and RSC were captured using NF-Y subunits as bait. Resolution of the structural compositions of SWI/SNF and RSC in both fungi revealed four novel filamentous-fungi-specific subunits: SWI/SNF-specific Fif1/Fif2 and RSC-specific Fif3/Fif4. These subunits distinguish filamentous fungal SWI/SNF and RSC from their counterparts in unicellular yeast and multicellular metazoans. SWI/SNF subunits (Snf19/Sol1/Fif1) and RSC subunits (Rsc7/Fif3) serve as direct targets for NF-Y-CRC interaction. Functional studies of the *P. oxalicum* NF-Y subunits PoNF–YB and PoNF–YC, along with the SWI/SNF–specific PoFif1 and the RSC–specific PoFif3, revealed their overlapping yet distinct roles in fungal growth, development, and cell wall-degrading enzyme production. The chromatin occupancy of PoFif1 and PoFif3 at certain target promoters is PoNF–Y-dependent. These findings suggest a model in which NF-Y interacts with the CRCs to coordinately regulate these growth, developmental, and metabolic processes. This model may explain how a canonical TF exhibits “pseudo-chromatin remodeler” activity: through its engagement with authentic CRCs, namely, SWI/SNF and RSC.

## Graphical Abstract

**Graphical Abstract:**
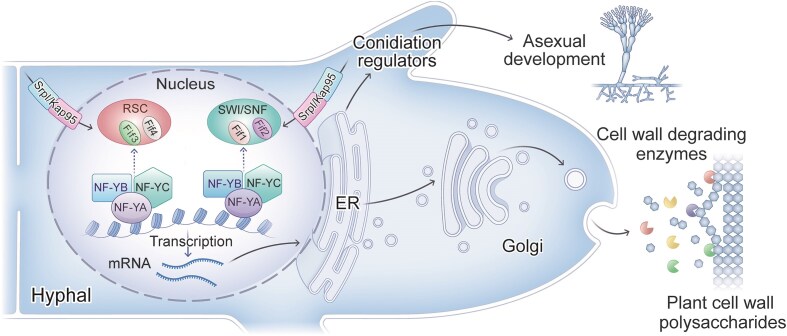

## Introduction

The NF-Y (Nuclear Factor Y) complex, also known as the CCAAT-binding factor (CBF) or HAP2/3/5 complex, is a heterotrimeric transcription factor (TF) composed of NF-YA (CBF-B/HAP2), NF-YB (CBF-A/HAP3), and NF-YC (CBF-C/HAP5) subunits, evolutionarily conserved from yeast to humans [1, 2]. The complex binds the CCAAT box, a core *cis-*regulatory element located 60–100 bp upstream of the transcription start site (TSS) in ∼30% of eukaryotic promoters [3]. The complex is termed master TF or “ruler” TF as it plays key roles in regulating diverse biological processes (BPs) such as cell cycle dynamics, development, differentiation, and stress adaptation across yeasts, invertebrates, plants, and mammals [4–6]. While eukaryotic TFs typically require cofactors (co-activators/co-repressors) to function as effectors of TFs [7], the identity of cofactors associated with the NF-Y complex remains elusive, despite its role as a key TF governing pleiotropic gene expression.

NF-Y’s regulatory role in chromatin structural dynamics is well-documented. In mammals, NF-Y functions in nucleosomal disruption [8]. Its binding to the CCAAT box is critical for establishing a local open chromatin configuration at the promoter of the fibroblast growth factor receptor 2 gene [9]. NF-Y facilitates master TF binding through chromatin conformational optimization and maintains transcriptional fidelity by preserving nucleosome-depleted promoter regions, suggesting its function as a “pseudo-chromatin remodeler” that mimics ATP-dependent chromatin remodeling activity [10, 11]. Beyond mammals, NF-Y has been reported to influence chromatin structure in fungi as well. In *Aspergillus nidulans*, disruption of the NF-Y complex abolishes the DNase I-hypersensitive site and eliminates nucleosome positioning at the transcriptional start site in the 5′ region of the target gene [12]. In *Trichoderma reesei*, mutation of the NF-Y binding motif, the CCAAT box, can induce a shift in nucleosome positioning [13]. However, the underlying mechanisms responsible for NF-Y’s chromatin-modifying function remain enigmatic.

Chromatin remodeling complexes (CRCs) utilize energy derived from ATP hydrolysis to displace, eject, destabilize, or remodel chromatin [14], thereby restructuring chromatin architecture and regulating DNA accessibility. This function governs essential nuclear processes, including DNA replication, repair, recombination, chromosome segregation, and transcription [15]. Typically, eukaryotic cells contain multiple types of CRCs. Four families of CRCs have been identified in *Saccharomyces cerevisiae*: switch/sucrose non-fermentable (SWI/SNF), imitation switch (ISWI), chromodomain helicase DNA-binding, and inositol requiring 80 (INO80) [16]. The yeast SWI/SNF complex was the first CRC to be characterized [17]. The SWI/SNF family includes at least two subfamilies: one is yeast SWI/SNF (*Drosophila* BAP and human BAF or hSWI/SNF-α), the other is yeast RSC (Remodels the Structure of Chromatin) (*Drosophila* PBAP and human PBAF or SWI/SNF-β) [18].

Structural studies have elucidated SWI/SNF and RSC complexes in model organisms like *S. cerevisiae* and humans. The SWI/SNF and RSC from various organisms typically have 12–17 subunits, including the core ATPase subunit, actin-related protein (ARP), body modules, and organism-specific accessory subunits [19–23]. The SWI/SNF and RSC complexes exhibit both shared and distinct subunit compositions. For example, while *S. cerevisiae* SWI/SNF (ScSWI/SNF) and RSC (ScRSC) share the Arp7 and Arp9 subunits, each complex also contains its own specific subunits, such as ScSWI/SNF specific Snf5, Snf6, and Snf11, and ScRSC specific Sfh1, Rsc1, and Rsc7 [24].

Despite evolutionary conservation of SWI/SNF and RSC architecture across eukaryotes, significant compositional heterogeneity is also evident in different organisms. Comparative analyses reveal species-specific subunit variations. For example, ScSWI/SNF uniquely contains Snf6, Snf11, and Taf14; human BAF complexes feature distinct subunits including BAF45A/B/C, BRD9, and BCL11A/B [20, 21]. These species-specific adaptations suggest functional diversification of chromatin remodeling machinery across eukaryotes.

Eurotiomycetes and Sordariomycetes represent the most extensively studied fungal classes in mycological research. These two taxonomic groups collectively account for over 40% of all sequenced fungal genomes within the fungi kingdom (data from MycoCosm of JGI, as of 1 May 2025), demonstrating their key roles in fungal research. Filamentous fungi from these two groups, such as *Trichoderma, Neurospora, Magnaporthe, Fusarium, Metarhizium*, and *Beauveria* in the Sordariomycetes and *Penicillium, Aspergillus, Epidermophyton, Trichophyton, Chaetothyriales*, and *Capnodiales* in the Eurotiomycetes group, are important research objects because of their extensive applications in biomedicine, agriculture, industry, and biotechnology [25–27]. From an evolutionary perspective, filamentous fungi developed a unique route to multicellularity, manifesting complicated or unusual life cycles and genetic architectures that differ from those observed in animals or plants [28, 29]. However, despite detailed characterization of SWI/SNF and RSC in unicellular yeasts and multicellular metazoans, the subunit composition and functional roles of these CRCs in filamentous fungi remain unexplored.

Here, we report that SWI/SNF and RSC act as cofactors for the NF-Y complex in the two representative filamentous fungi *Penicillium oxalicum* (Eurotiomycetes) and *T. reesei* (Sordariomycetes). We resolved the complete subunit architectures of SWI/SNF and RSC complexes in both species, defined how NF-Y physically interacts with specific subunits within SWI/SNF and RSC assemblies, and proposed models for how these complexes coordinately regulate key fungal BPs.

## Materials and methods

### Fungal strains and media

All strains used in this study are listed in Supplementary Spreadsheet S1. The wild-type (WT) *P. oxalicum* 114-2 (CGMCC 5302) and *T. reesei* QMP served as parent strains [30, 31]. The *P. oxalicum* WT strain and mutants generated in this study were grown on wheat bran agar (10% w/v wheat bran juice) at 30°C for 5 days for conidiation, while the *T. reesei* WT strain and mutants were grown on potato dextrose agar at 30°C for 7 days for conidiation. For phenotypic analysis, tandem affinity purification, real-time quantitative polymerase chain reaction (PCR), and transcriptome analysis, the following media were used: potato dextrose broth (PDB); Vogel’s Minimal Medium (1 × VMM) [32] supplemented with one of the following carbon sources: 2% (w/v) glucose (VMMG), 2% (w/v) xylose (VMMX), 2% (w/v) glycerol (VMMGly), 2% (w/v) ball-milled cellulose (VMMC), or 1% ball-milled cellulose plus 1% wheat bran (VMMCW). These carbon sources were selected to assess fungal growth and gene expression under conditions of readily metabolizable sugars (glucose, xylose, glycerol) versus complex plant polymers (cellulose, wheat bran). The above media were added with 1.5% agar for solid culture.

### Construction of different mutants

The gene names, corresponding loci, and UniProt entries of the proteins involved in this study are shown in Supplementary Spreadsheet S2. The primers used in this study are shown in Supplementary Spreadsheet S3.

To use the PoNF-YA, PoNF-YB, and PoNF-YC subunits of the *P. oxalicum* NF-Y complex as bait proteins for tandem affinity purification and mass spectrometry (TAP-MS), we constructed three corresponding HA-FLAG-tagged strains: PoYA-TAP, PoYB-TAP, and PoYC-TAP. The construction strategies and verification of these strains are shown in Supplementary Fig. S1. To use the PoSnf21, PoFif1, PoFif2, and PoFif3 subunits of the *P. oxalicum* SWI/SNF and RSC complexes as bait proteins for TAP-MS, we constructed four corresponding HA-FLAG-tagged strains: the PoSnf21-TAP, PoFif1-TAP, PoFif2-TAP, and PoFif3-TAP strains. The construction strategies and verification of these strains are shown in Supplementary Fig. S2. For example, to construct the PoYA-TAP strain, primer pairs PoYA-TAP-UF/PoYA-TAP-UR and PoYA-DF/PoYA-DR were used to amplify the 5′-upstream and 3′-downstream homologous arms of the PoNF-YA gene using *P. oxalicum* 114–2 genomic DNA as the template. Primer pair hph-F/hph-R was used to amplify the hygromycin B resistance gene (*hph*) from plasmid pSilent-1 [33]. The three PCR fragments (5′-upstream region, 3′-downstream region of PoNF-YA, and *hph*) were then fused by fusion PCR. The resulting product was amplified using nested primers PoYA-TAP-CSF/PoYA-CSR and transformed into *P. oxalicum* to generate the PoYA-TAP mutant. Proper integration of the FLAG-HA tag was verified by sequencing (Supplementary Fig. S1A–C).

To use the TrNF-YC, TrFif1, and TrFif3 subunits of the *T. reesei* NF-Y, SWI/SNF and RSC complexes as bait proteins for TAP-MS, we constructed three corresponding HA-FLAG-tagged strains: TrYC-TAP, TrFif1-TAP, and TrFif3-TAP. The construction strategies and verification of these strains are shown in Supplementary Fig. S3. For example, to construct the TrYC-TAP strain, primer pairs TrYC-TAP-UF/TrYC-TAP-UR and TrYC-TAP-DF/TrYC-TAP-DR were used to amplify the 5′-upstream and 3′-downstream homologous arms of the TrNF-YC gene using *T. reesei* QMP genomic DNA as the template. Primer pair pyrG-F/pyrG-R was used to amplify the *pyrG* marker gene using *A. nidulans* genomic DNA as the template. The three PCR fragments (5′-upstream region, 3′-downstream region of TrNF-YC, and *pyrG*) were fused by fusion PCR. The resulting product was amplified using nested primers TrYC-TAP-CSF/TrYC-TAP-CSR and transformed into *T. reesei* to generate the TrYC-TAP mutant. Proper integration of the FLAG-HA tag was verified by sequencing (Supplementary Fig. S3A–C).

To determine the subcellular localization of PoFif1, PoFif2, PoFif3, and PoNF-YC, we constructed Fif1-GFP, Fif2-GFP, Fif3-GFP, and YC-GFP strains. The construction strategies and verification results for these strains are shown in Supplementary Fig. S4. For example, to construct the Fif1-GFP strain, the 5′- and 3′-homologous arms of the PoFif1 locus were amplified with primer pair Fif1-GFP-UF/Fif1-GFP-UR, using *P. oxalicum* 114–2 genomic DNA as a template. The *gfp* gene was amplified from plasmid pEGFP with primers gfp-F/gfp-R, and the *hph* gene was amplified from plasmid pSilent-1 with primers hph-F/hph-R. The four PCR fragments (Fif1 5′-upstream region, Fif1 3′-downstream region, *gfp*, and *hph*) were fused by fusion PCR. The resulting product was amplified using nested primers Fif1-GFP-CSF/Fif1-CSR and transformed into *P. oxalicum* WT to generate the Fif1-GFP strain.

To delete the genes Pofif1, Pofif3, PoNF-YB, and PoNF-YC, we constructed the Δfif1, Δfif3, ΔYB, and ΔYC strains. The construction strategies and verification data for all strains are shown in Supplementary Fig. S5. For example, to construct the Δfif1 strain, primer pairs ΔFif1-UF/ΔFif1-UR and Fif1-DF/Fif1-DR were used to amplify the 5′-upstream and 3′-downstream homologous arms of the Pofif1 gene using *P. oxalicum* 114–2 genomic DNA as the template. Primer pair hph-F/hph-R was used to amplify the *hph* marker gene from plasmid pSilent-1. The three PCR fragments (5′-upstream region, 3′-downstream region of Pofif1, and *hph*) were fused by fusion PCR. The resulting product was amplified using nested primers ΔFif1-CSF/Fif1-CSR and transformed into *P. oxalicum* to generate the Δfif1 mutant.

To generate strains for chromatin immunoprecipitation-quantitative PCR (ChIP-qPCR) experiments by deleting the genes of PoNF-YB and PoNF-YC in the Fif1-TAP and Fif3-TAP backgrounds, respectively, we constructed the Fif1-TAP-ΔYB, Fif1-TAP-ΔYC, Fif3-TAP-ΔYB, and Fif3-TAP-ΔYC strains. The construction strategies and verification data for all strains are shown in Supplementary Fig. S6. For example, to construct the Fif1-TAP-ΔYB strain, primer pairs ΔYB-UF/Fif1-TAP-ΔYB-UR and ΔFif1-TAP-ΔYB-DF/YB-DR were used to amplify the 5′-upstream and 3′-downstream homologous arms of the PoNF-YB gene. Primer pairs ptrA-F/ptrA-R were used to amplify the pyrithiamine resistance gene (*ptrA*) from plasmid pME2892. Then, the three PCR fragments (5′- and 3′-flanking regions of the PoNF-YB gene and *ptrA*) were fused with fusion PCR. The fused product was then amplified using nested primers ΔYB-CSF/YB-CSR and was transformed into the Fif1-TAP to obtain the Fif1-TAP-ΔYB mutant.

### Tandem affinity purification and mass spectrometry

Fresh conidial suspensions of the HA-FLAG-tagged TAP strains (*P. oxalicum*: PoYA-TAP, PoYB-TAP, PoYC-TAP, PoSnf21-TAP, PoFif1-TAP, PoFif2-TAP, PoFif3-TAP; *T. reesei*: TrYC-TAP, TrFif1-TAP, TrFif3-TAP) and their respective untagged parental control strains (*P. oxalicum* 114–2 and *T. reesei* QMP) were inoculated into 1 L of VMMG liquid and cultivated at 30 °C and 200 rpm for 24 h. The mycelia were collected, filtered, and washed twice with 0.96% NaCl (w/v) containing 1% dimethyl sulfoxide and 1 mM phenylmethylsulfonyl fluoride. Then, the mycelia were ground with liquid nitrogen, transferred to a 50 ml centrifuge tube, and supplemented with 15 ml of protein lysis buffer (NaCl 9 g, 1 M Tris–HCl, pH 7.5, glycerol 100 ml, and NP40 1 ml, per 1 l) and 0.05% Protease Inhibitor Cocktail (MedChemExpress, China). After thoroughly mixing the mycelia with the lysis buffer, the mixture was placed on ice for 10 min. The samples were centrifuged at 10 000 rpm and 4°C for 30 min to obtain the supernatant. For the first-step affinity purification, ANTI-FLAG M2 affinity resin (Smart-lifesciences, China) was added to the supernatant and incubated overnight at 4°C with gentle rotation. 3 × FLAG peptide (500 μl, 150 ng/μl) was used to elute the bound proteins from the ANTI-FLAG M2 affinity resin to obtain the first protein eluate. For the second-step affinity purification, ANTI-HA resin (Smart-lifesciences, China) was added to the first protein eluate and incubated overnight at 4°C with gentle rotation. Finally, 80 μl of 8 M urea was used to elute the bound proteins from the ANTI-HA resin, yielding the final protein eluate, which contained the putative interacting proteins of the bait protein.

The final eluate was divided into three parts. One part was separated by 12.5% sodium dodecyl sulfate–polyacrylamide gel electrophoresis (SDS–PAGE) and stained with silver stain. One part was analyzed using western blot with anti-HA antibody (ABclonal, China). The other part was analyzed by LC-MS/MS (APT, Shanghai, China) to identify the putative interacting proteins of the bait protein. Protein digestion was performed using trypsin. The resulting peptides were analyzed via LC-MS/MS using a Q Exactive mass spectrometer coupled to an Easy-nLC 1000 system (Thermo Fisher Scientific). The raw mass spectrometry data were analyzed using Mascot 2.2.2 software [34]. Proteins identified in the HA-FLAG-tagged TAP strains but absent in their corresponding untagged controls were considered reliable interaction candidates. emPAI was used for the estimation of absolute protein amount by using the following formula emPAI = 10^PAI^ – 1 [35]. PAI = *N*_observed_/ *N*_observable_. *N*_observed_ is the number of experimentally observed peptides identified by LC-MS/MS, and *N*_observable_ is the number of theoretically observable tryptic peptides according to the amino acid sequence of each protein. Theoretical peptide counts (*N*_observable_) were derived from PeptideMass of Expasy (https://web.expasy.org/peptide_mass/).

### Yeast two-hybrid assay

Yeast two-hybrid (Y2H) assays were conducted according to the Matchmaker Gold Yeast Two-Hybrid System manual (Takara Bio, Japan). Briefly, the coding sequences (CDSs) of *P. oxalicum* NF-Y subunits were individually cloned into the pGADT7 vector, which contains an activation domain (AD), and transformed into *S. cerevisiae* strain Y187. Similarly, the CDSs of *P. oxalicum* SWI/SNF and RSC subunits were individually cloned into the pGBKT7 vector, which contains a DNA-binding domain (BD), and transformed into *S. cerevisiae* strain Y2HGold. All transformed yeast strains were verified for the absence of autoactivation and toxicity prior to interaction testing. Y187 strains containing AD-fusion constructs and Y2HGold strains containing BD-fusion constructs were co-incubated to allow mating. Diploid cells resulting from successful mating, identifiable by their characteristic “Mickey Mouse” morphology, were subsequently selected. Diploid cells were assessed for protein–protein interactions by cultivation on quadruple-dropout medium (QDO: SD/-Ade/-His/-Leu/-Trp) and on QDO supplemented with X-α-Gal and aureobasidin A (QDO/X-α-Gal/Aba). Plates were incubated at 30°C for 3–5 days, and interactions were scored on the basis of colony growth and the appearance of blue coloration. For each mating experiment, positive and negative controls were included to validate the system. The positive control consisted of Y2HGold harboring pGBKT7-53 (encoding BD-p53) mated with Y187 harboring pGADT7-T (encoding AD-SV40 large T-antigen). The negative control comprised Y2HGold harboring pGBKT7-Lam (encoding BD-Lamin) mated with Y187 harboring pGADT7-T.

### Phenotypic analysis

For observation of colony morphology, 1 µl of fresh conidia suspension (10^7^ conidia ml^−1^) was point-inoculated onto VMMG, VMMX, VMMGly, or VMMC agar and then cultivated at 30°C. Colony diameters were measured daily for 7 days. For conidial quantification, 200 µl of conidial suspension (10^7^ conidia ml^−1^) was spread evenly on VMMG agar. After 5-day incubation at 30°C, 10-mm diameter agar plugs were excised, and conidia eluted in 2 ml physiological salt (0.2% w/v Tween 80 and 0.8% w/v NaCl) by vortex mixing. Conidial counts were determined using a hemocytometer. Hyphal and conidial development was examined microscopically after staining with lactophenol cotton blue (0.05% w/v cotton blue, 20% w/v phenol, 40% v/v glycerol, 20% v/v lactic acid in distilled water) for 20 min. Hyphal branching was evaluated by random observation of 50 hyphal elements (Nikon Eclipse 80i, 20 × objective). Hyphal growth unit length (L_hgu_) was calculated according to [36].

### Subcellular localization observation

Hyphae from GFP-tagged strains were examined using a high-sensitivity laser scanning confocal microscope (ZEISS LSM880; Carl Zeiss, Germany). Nuclei were stained with Hoechst 33 342 (Sigma–Aldrich, USA) for 15 min in the dark. GFP fluorescence was detected under 488 nm excitation. Hoechst-stained nuclei were visualized under 405 nm excitation.

### Real-time quantitative PCR

Fresh spores (1 × 10^7^ spores/ml) were pre-cultured in VMMGly at 30°C for 22 h. Equal mycelial aliquots (0.3 g) collected by vacuum filtration were transferred to 50 ml of fresh medium (VMMG, VMMGly, VMMCW, or PDB) and incubated at 30°C with 200 rpm shaking for 24 h. Harvested mycelia were fully ground in liquid nitrogen, followed by total RNA extraction using RNAiso Plus reagent (Takara, Japan). Complementary DNA was synthesized using the PrimeScript™ RT reagent Kit with gDNA Eraser (Takara, Japan). Quantitative PCR was performed on a LightCycler® 480 system (Roche, USA) with SYBR® Premix Ex Taq™ (Takara, Japan), using primers listed in Supplementary Spreadsheet S2. The amplicon lengths ranged from 100 to 200 bp. All RT-qPCR experiments were performed using three independent biological replicates, with gene expression copy numbers calculated via gene-specific standard curves and normalized to *actin* gene expression levels.

### Transcriptome analysis

Strains WT, Δfif1, and Δfif3 were cultivated in triplicate as previously described in the section “Real-time quantitative PCR.” Fresh mycelia were harvested and ground in liquid nitrogen after 24 h of cultivation in VMMG or VMMCW. Total RNA was extracted with RNAiso Plus reagent (Takara, Japan), and incubated with 10 U DNase I at 37°C for 30 min to remove genomic DNA. Messenger RNA (mRNA) was enriched from total RNA using Oligo(dT) magnetic beads prior to library construction. The quality of the mRNA samples was assessed [OD_260_/OD_280_: 1.8–2.2; OD_260_/OD_230_: >1.5; RNA integrity number: >8.0] before subsequent library construction. Transcriptome sequencing based on BGISEQ- 500 RNA-seq was performed by the Beijing Genomics Institute (BGI, Shenzhen, China). Saturation analysis of each sample was performed to determine its availability for omics analysis. The filtered clean reads for each gene were normalized to fragments per kilobase of transcript per million mapped reads (FPKM) for differential expression analysis. The fold change (FC) for differential expression was calculated by comparing the mean FPKM value from the three replicates of the mutant to that of the control WT strain. Significantly differentially expressed genes (DEGs) between samples were identified through a significance test with combined thresholds |(log_2_(FoldChange)| ≥ 1 and *Q*-value < 0.05). ShinyGO 0.82 (https://bioinformatics.sdstate.edu/go/) was used for Gene Ontology (GO) annotation and functional enrichment analysis with a threshold of FDR ≤ 0.05. Genesis software was used for cluster analysis [37].

### Chromatin immunoprecipitation-quantitative PCR

Strains WT, ΔYB, ΔYC, Fif1-TAP, Fif1-TAP–ΔYB, Fif1-TAP–ΔYC, Fif3-TAP, Fif3-TAP–ΔYB, and Fif3-TAP–ΔYC were cultivated in triplicate as previously described in the section “Real-time quantitative PCR.” The ChIP experiments were conducted according to the established protocol [38] with minor adjustments. Briefly, fungal hyphae grown in VMMG or VMMC medium were cross-linked using 1% formaldehyde for 10 min at 30°C and 200 rpm with shaking. To terminate the cross-linking reaction, glycine was added to a final concentration of 125 mM. The resulting mycelia were collected via vacuum filtration, ground in liquid nitrogen, and resuspended in a ChIP-lysis buffer (50 mM HEPES at pH 7.5, 150 mM NaCl, 1 mM ethylenediaminetetraacetic acid, 0.5% Triton X-100, 0.1% sodium deoxycholate, 0.1% SDS, 1 mM PMSF, and 0.1% protease inhibitor cocktail). To obtain chromatin fragments ranging from 200 to 500 bp, the mixture was subjected to sonication. Subsequently, equal amounts of extracted chromatin (1 mg) was utilized for immunoprecipitation (IP) with an anti-HA antibody (Proteintech, USA). Both the obtained IP products and 0.1 mg input chromatin DNA (without IP) of each sample were subjected to RNase treatment to eliminate RNA, followed by proteinase K digestion and incubation at 65°C to reverse the DNA-protein cross-links. The purified DNA from both IP and input samples was recovered through phenol-chloroform extraction and ethanol precipitation. Finally, the DNA was quantified by real-time PCR. The primers are listed in Supplementary Spreadsheet S2. The enrichment level, termed ChIP efficiency, was determined using the percent of input method [39] with the following formula: ChIP efficiency = 2^−ΔCt^ × 100%, ΔCt = Ct_IP_−(Ct_Input _− log_2_10).”

### Statistical analysis

Unless otherwise specified (e.g. *L*_hgu_ was calculated by randomly selecting 50 hyphal elements), all quantitative experiments in this study were performed with three biological replicates. Data are presented as the mean ± standard deviation. Differences between two groups were assessed using an unpaired, two-tailed Student’s *t*-test. A *P*-value of < .05 was considered statistically significant, denoted in the text and figures as *P < .05, **P < .01, and ***P < .001.

## Results

### NF-Y complex preys on the CRCs SWI/SNF and RSC in both fungi

For the TAP-MS experiments in *P. oxalicum* and *T. reesei*, proteins detected in the HA-FLAG-tagged TAP strains were considered reliable interactors only if they were absent from all three biological replicates of both corresponding untagged parental controls (*P. oxalicum* 114–2 and *T. reesei* QMP). The complete ranked lists of the control strains are provided in Supplementary Spreadsheets S4, Sheet 9 for *P. oxalicum* 114–2 and S5, Sheet 4 for *T. reesei QMP*; the top 10 proteins identified in each control strain are presented in Supplementary Fig. S7. All proteins were ranked in descending order based on their emPAI values [35].

First, TAP-MS was performed using the *P. oxaliucm* PoNF-YC subunit as bait. SDS–PAGE and western blot analyses confirmed the presence of the bait protein PoNF-YC in the TAP eluates from the HA-FLAG-tagged strain PoYC-TAP, in contrast to the untagged control *P. oxalicum* 114–2 (Supplementary Fig. S8A and B, red arrows). The complete ranked list of reliable interactors from the PoYC-TAP is provided in Supplementary Spreadsheet S4, Sheet 1. 17 proteins were observed in the intersection of three biological replicates of PoYC-TAP samples but not in any of the controls (Supplementary Fig. S8C). Among these, three subunits of the NF-Y complex ranked as the top three (Supplementary Fig. S8D, red dots/backgrounds), and the shared subunits of SWI/SNF and RSC, PoRSC8 and PoSnf21 were also detected (Supplementary Fig. S8D, yellow dots/backgrounds). Notably, in the first sample (PoYC-TAP-1), alongside PoRSC8 and PoSnf21, four other shared subunits of SWI/SNF and RSC (PoArp4, PoSsr3, PoArp9, and PoSsr4), the SWI/SNF specific subunits (PoSnf5, PoSnf59, and PoTaf14), and the RSC specific subunits (PoSfh1, PoRsc1, and PoRsc7) were also identified (Supplementary Fig. S8E). The second sample (PoYC-TAP-2) further detected the SWI/SNF specific subunit PoSnf5 and the RSC specific subunit PoSfh1. These findings suggest a close association between the NF-Y complex and the CRCs SWI/SNF and RSC.

**Figure 1.**
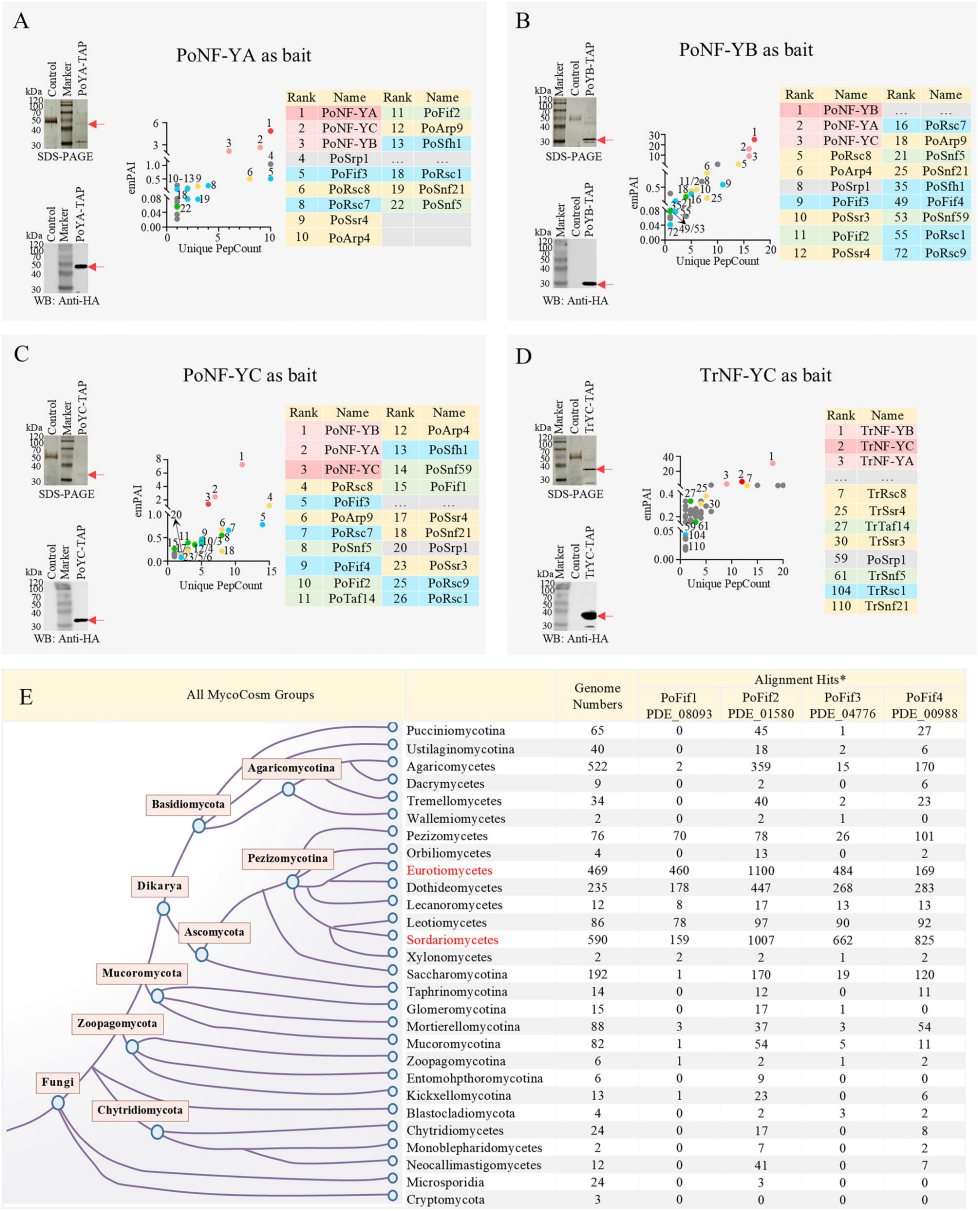
The TAP-MS results of using the subunits of NF-Y as bait in *P. oxaliucm* and *T. reesei*. (**A**–**D**) SDS–PAGE, WB, and protein rank analysis of TAP using PoNF-YA, PoNF-YB, PoNF-YC, and TrNF-YC as the baits, respectively. The list of identified proteins was pre-filtered to exclude proteins detected in the control samples, and then ranked by emPAI value from highest to lowest. Dark red dots/backgrounds, the bait proteins. Light red dots/backgrounds, the two non-bait subunits of the NF-Y complex. Yellow dots/backgrounds, the shared subunits between SWI/SNF and RSC. Green dots/backgrounds, SWI/SNF-specific subunits. Blue dots/backgrounds, RSC-specific subunits. Not all ranking positions are displayed in the figure. The emPAI values for all samples and complete ranked lists are provided in Supplementary Spreadsheets S4, Sheets 2–4 and S5, Sheet 1. (**E**) The distribution of homologous proteins of PoFif1, PoFif2, PoFif3, and PoFif4 in kingdom fungi. The information on the tree (left) was retrieved from MycoCosm of JGI (https://mycocosm.jgi.doe.gov/mycocosm/home) [43]. The red words (Eurotiomycetes and Sordariomycetes) refer to the classes to which *P. oxalicum* and *T. reesei* belong, respectively. The number of fungal genomes is as of 12 January 2025. BlastP was performed using proteins of PoFif1, PoFif2, PoFif3, and PoFif4 as queries, respectively. *, Expect, 1.0E-6; Scoring Matrix, BLOSUM62; Score > 200.

Subsequently, we used the three subunits of PoNF-Y from *P. oxaliucm* and one TrNF-YC subunit of TrNF-Y from *T. reesei* as baits for TAP-MS (Fig. 1). Specifically, after using PoNF-YC as bait, we repeated the experiment with PoNF-YA and PoNF-YB (Fig. 1A–C). Although replicates were not conducted for each NF-Y subunit in this experiment, the fact that all three subunits belong to the same trimeric NF-Y complex allows them to be considered biological replicates. The reliable interactors identified by MS from the eluates of PoYA-TAP, PoYB-TAP, and PoYC-TAP are presented in Supplementary Spreadsheet S4, Sheets 2–4. SDS–PAGE and western blot analyses confirmed the presence of the bait proteins in all three TAP eluates. Compared with the untagged control, the HA-FLAG-tagged strains (PoYA-TAP, PoYB-TAP, and PoYC-TAP) displayed specific bands of ~42, 30, and 35 kDa, respectively (Fig. 1A, red arrows). These observed sizes closely match the theoretical molecular weights of the bait subunits PoNF-YA (37.4 kDa), PoNF-YB (24.5 kDa), and PoNF-YC (29.4 kDa).

In the TAP-MS of PoYA-TAP, PoYB-TAP, and PoYC-TAP, the top three positions were exclusively occupied by the three NF-Y subunits (Fig. 1A–C, dark/light red dots/backgrounds), even though the bait subunit (dark red dots/backgrounds) did not necessarily rank first. Additionally, we identified 11, 15, and 17 subunits of the *P. oxalicum* SWI/SNF (PoSWI/SNF) and/or PoRSC complexes in the eluates of PoYA-TAP, PoYB-TAP, and PoYC-TAP, respectively (Fig. 1A–C and Supplementary Spreadsheet S4, Sheets 2–4).

For example, with PoNF-YC as the bait, among the top 26 reliable proteins (including the top three NF-YA/B/C subunits), 13 homologs of known SWI/SNF and RSC subunits were identified (Fig. 1C). These 13 proteins included: (i) Subunits shared between SWI/SNF and RSC: core ATPase PoSnf21 (18th), actin module subunits PoArp9 (6th), and PoArp4 (12th), body module subunits PoRsc8 (4th), PoSsr4 (17th), and PoSsr3 (23rd) (Fig. 1C, yellow dots/backgrounds); (ii) SWI/SNF-specific subunits: PoSnf5 (8th), PoTal14 (11th), and PoSnf59 (14th) (Fig. 1C, green dots/backgrounds); and (iii) RSC-specific subunit PoRsc7 (7th), PoSfh1 (13th), PoRsc9 (25th), and PoRsc1 (25th) (Fig. 1C, blue dots/backgrounds). Additionally, four uncharacterized filamentous-fungi-specific CRC subunits (named PoFif1–4) showed strong association with both complexes. Subsequent validation confirmed PoFif1 (15th) and PoFif2 (10th) as SWI/SNF-specific and PoFif3 (5th) and PoFif4 (9th) as RSC-specific. Specifically, among these top 26 proteins, 17 corresponded to components of PoSWI/SNF and PoRSC complexes. All 12 subunits (later-defined) of PoRSC were captured within this set, and 11 of 12 PoSWI/SNF subunits (later-defined) were captured (Fig. 1C), representing near-complete (17 of 18 subunits) coverage of both complexes' subunits.

Next, we performed TAP-MS in *T. reesei* using its TrNF–YC subunit as bait (one sample) (Fig. 1D, Supplementary Spreadsheets S5, Sheet 1). Compared with the untagged control, the HA-FLAG-tagged strain TrYC-TAP showed a specific band at ~35 kDa (Fig. 1D, red arrow), which closely matches the theoretical molecular weight (31.5 kDa) of the bait subunit TrNF-YC (Fig. 1D). Subunits of both the *T. reesei* SWI/SNF (TrSWI/SNF) and TrRSC complexes were observed, including the shared subunits TrSnf21, TrRsc8, TrSsr4, and TrSsr3 (Fig. 1D, yellow dots/backgrounds); the SWI/SNF-specific subunits TrTaf14 and TrSnf5 (Fig. 1D, green dots/backgrounds); and the RSC-specific subunit TrRsc1 (Fig. 1D, blue dots/backgrounds) (Supplementary Spreadsheet S5, Sheet 1).

Collectively, these results demonstrate a robust interaction between the NF-YA/B/C complex and the two CRCs: SWI/SNF and RSC. Interestingly, however, the identified SWI/SNF and RSC subunits ranked considerably lower in *T. reesei* than in *P. oxalicum*. This discrepancy is likely attributable to the use of total mycelial lysates rather than nuclear extracts in the TAP experiments. *T. reesei*, characterized by its potent protein secretion capacity [40, 41], exhibits a high abundance of cytoplasmic proteins involved in protein folding and secretion [42]. Indeed, we also observed that molecular chaperones associated with protein folding and secretion, such as Hsp60 (6th) and Hsp70 (12th), appeared at relatively high ranks (Supplementary Spreadsheet S5, Sheet 1). This elevated cytoplasmic protein background in *T. reesei* mycelial lysates contributes to an increased false-positive rate and thereby lowers the relative ranks of genuine nuclear interaction partners.

### Identification of four uncharacterized filamentous-fungal-specific subunits associated with the CRCs

As the complete subunit architecture of SWI/SNF and RSC complexes in filamentous fungi remains uncharacterized, this study first aims to define their compositions.

In yeast, the ATPase subunits of ScSWI/SNF and ScRSC complexes are Snf2 [Snf22 in *Schizosaccharomyces pombe* SWI/SNF (SpSWI/SNF)] and Sth1 [Snf21 in *S. pombe* RSC (SpRSC)] proteins, respectively. However, when we performed BLAST searches for ATPase subunits in *P. oxalicum* and *T. reesei* using ScSnf2/ScSth22 and SpSth1/SpSnf21 as queries, only one protein (UniProt S7ZVH2) in *P. oxalicum* and one protein (UniProt G0RDG7) in *T. reesei* were identified as homologs of these ATPase subunits. This finding indicated that SWI/SNF and RSC in both fungi share a core ATPase subunit Snf21, unlike yeast where SWI/SNF and RSC each have distinct core subunits. S7ZVH2 and G0RDG7 were named PoSnf21 and TrSnf21, respectively. Our original plan was to obtain the respective components of SWI/SNF or RSC from the two distinct ATPase subunits, but the existence of a single core ATPase subunit disproved this plan. Nevertheless, we can still use TAP-MS to prey on all components of SWI/SNF or RSC using Snf21 as the bait and then classify two complexes based on their specific subunits.

**Figure 2.**
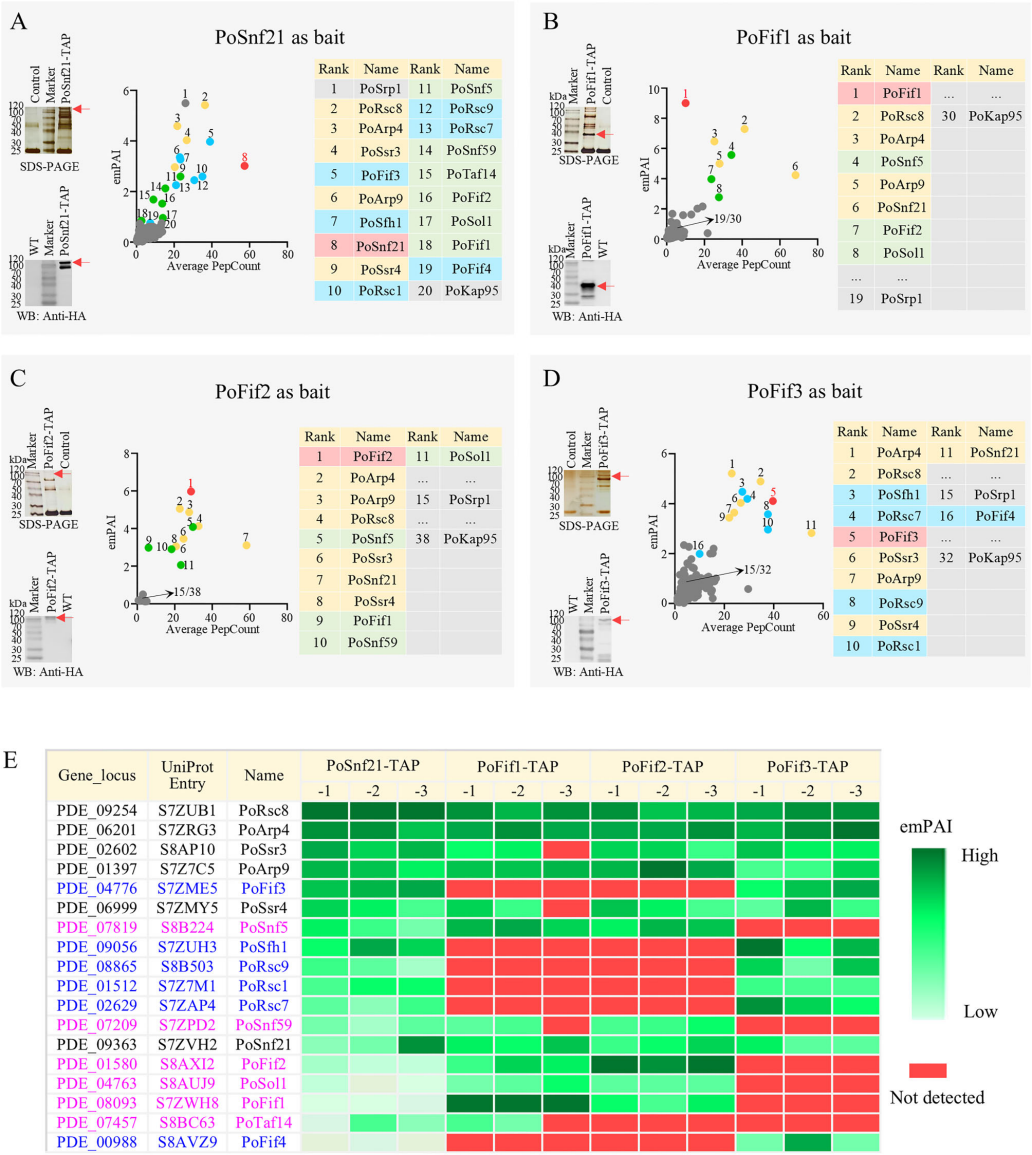
The determination of compositions of SWI/SNF and RSC complexes in *P. oxalicum*. (**A**–**D**) SDS–PAGE, WB, and protein rank analysis of TAP using PoSnf21, PoFif1, PoFif2, and PoFif3 as the baits, respectively. TAP-MS using PoFif1, PoFif2,and PoFif3 as the bait, respectively. Red arrows, the bait proteins. The theoretical molecular weights of four baits are 163.1, 33.1, 77.8, and 92.2 kDa, respectively. The list of identified proteins was pre-filtered to exclude proteins detected in the control samples and then ranked by emPAI value from highest to lowest. Red dots/ backgrounds, the bait proteins. Yellow dots/backgrounds, the shared subunits between SWI/SNF and RSC. Green dots/backgrounds, SWI/SNF-specific subunits. Blue dots/backgrounds, RSC-specific subunits. Gray dots/backgrounds, non-NF-Y or non-CRCs proteins, including Importin PoSrp1 (Importin α) and/or PoKap95 (Importin β). (**E**) TAP-MS results from triplicate samples of subunits of the CRCs in *P. oxalicum*. Black font: the shared subunits between SWI/SNF and RSC. Magenta font: SWI/SNF-specific subunits. Blue font: RSC-specific subunits. The emPAI gradient is represented by a green color scale (dark-green, high value; light-green, low value) for each TAP strain sample. Undetected proteins are marked in red. Not all ranking positions are displayed in the figure. The emPAI values for all samples and complete ranked lists are provided in Supplementary Spreadsheets S4, Sheets 5–8.

TAP-MS experiments were performed using PoSnf21, PoFif1, PoFif2, and PoFif3 from *P. oxalicum* as baits, respectively (Fig. 2). Fifty-four proteins were observed in all three HA-FLAG-tagged PoSnf21-TAP samples but not in any of the controls. They were shown in Fig. 2A and Supplementary Spreadsheet S4, Sheet 5. The bait PoSnf21 ranks eighth (Fig. 2A). The top 20 proteins include: (i) 14 proteins (ranks 2–4, 6, 7, 9–15, and 17) that are homologs of components in yeast SWI/SNF and/or RSC. They contain typical domains associated with CRCs, such as the SWI-SNF_Ssr4 domain in PoSsr4, Snf5 domains in PoSnf5 and PoSfh1; (ii) two proteins (rank 1 PoSrp1 and rank 20 PoKap95) that are homologs of subunits α and β of the heterodimer Importin in yeast; (iii) four previously uncharacterized proteins: three lacking specific domains (ranks 5, 16, and 18) and one bromodomain-containing protein (rank 19) (Fig. 2A and Supplementary Fig. S9).

SmartBLAST analysis (NCBI Landmark database) [44] confirmed that homologs of these four uncharacterized proteins are absent in 27 model organisms—including *S. cerevisiae, S. pombe, Arabidopsis thaliana, Drosophila melanogaster*, and *Mus musculus*—but are conserved within Eurotiomycetes, Dothideomycetes, Leotiomycetes, and Sordariomycetes in fungi kingdom (Fig. 1E). These classes belong to the subphylum Pezizomycotina, which comprises most known filamentous fungi [45]. Consequently, we designated them as filamentous-fungi-specific subunits PoFif1 (PDE_08 093), PoFif2 (PDE_01 580), PoFif3 (PDE_04 776), and PoFif4 (PDE_00 988). Homologs of PoFif1-4 and their sequence identities in representative filamentous fungi such as *Aspergillus, Trichoderma, Neurospora, Magnaporthe, Fusarium, Metarhizium*, and *Beauveria* are listed in Supplementary Spreadsheet S6.

### The filamentous-fungi-specific subunits distinguish *P. oxalicum* SWI/SNF and RSC complexes

Then, we performed TAP-MS using PoFif1, PoFif2, and PoFif3 as bait (Fig. 2B–D), respectively, to (i) ascertain whether they are components of SWI/SNF or RSC and (ii) distinguish SWI/SNF and RSC from each other. Proteins exclusively detected in triplicate PoFif1-TAP (71 proteins), PoFif2-TAP (19 proteins), and PoFif3-TAP (152 proteins) samples but absent in controls are listed in Supplementary Spreadsheet S4, Sheets 6–8.

When PoFif1 was used as the bait, the bait PoFif1 ranks first (Fig. 2B, red dots/background); proteins ranked second (PoRsc8), third (PoArp4), fifth (PoArp9), and sixth (PoSnf21) are shared subunits between SWI/SNF and RSC (Fig. 2B, yellow dots/background); the protein ranked in seventh is PoFif2; proteins ranked fourth (PoSnf5), and eighth (PoSol1) are SWI/SNF-specific subunits (Fig. 2B, green dots/background); and there were no RSC-specific subunits observed. Similarly, when PoFif2 was used as the bait, shared subunits between SWI/SNF and RSC and SWI/SNF-specific subunits were observed, but no RSC-specific subunits were observed (Fig. 2C).

In contrast, when PoFif3 was used as the bait, shared subunits between SWI/SNF and RSC (Fig. 2D, yellow dots/background) and RSC-specific subunits, including PoFif4 (ranked 16th) (Fig. 2D, blue dots/background) were observed, but there were no SWI/SNF-specific subunits observed.

For clarity, triplicate samples of PoSnf21-TAP, PoFif1-TAP, PoFif2-TAP, and PoFif3-TAP are presented together. PoFif1 and PoFif2 were consistently copurified, whereas PoFif3 and PoFif4 were consistently copurified. SWI/SNF-specific subunits (PoSnf5, PoSnf59, PoSol1, and PoTal14) were exclusively associated with PoFif1 and PoFif2, whereas RSC-specific subunits (PoSfh1, PoRsc1, PoRsc7, PoRsc9) were exclusively associated with PoFif3 and PoFif4 (Fig. 2E).

Furthermore, to confirm whether Fif1/Fif2 and Fif3/Fif4 are stable subunits of the SWI/SNF and RSC complexes, respectively, we conducted TAP-MS experiments with one sample each using PoFif1, PoFif2, and PoFif3 as bait under high-salt conditions (500 mM NaCl) (Supplementary Fig. S10 and Supplementary Spreadsheet S7). Both the PoFif1 and PoFif2 purifications recovered all 12 subunits of the SWI/SNF complex, including PoFif1 and PoFif2 themselves (Supplementary Fig. S10B and C). Similarly, purification with PoFif3 recovered all 12 subunits of the RSC complex, including PoFif4 (Supplementary Fig. S10D). These results indicate that Fif1/Fif2 are constitutive and stable subunits of the SWI/SNF complex, whereas Fif3/Fif4 are constitutive subunits of the RSC complex.

### Conserved composition of SWI/SNF and RSC complexes in *T. reesei*

**Figure 3.**
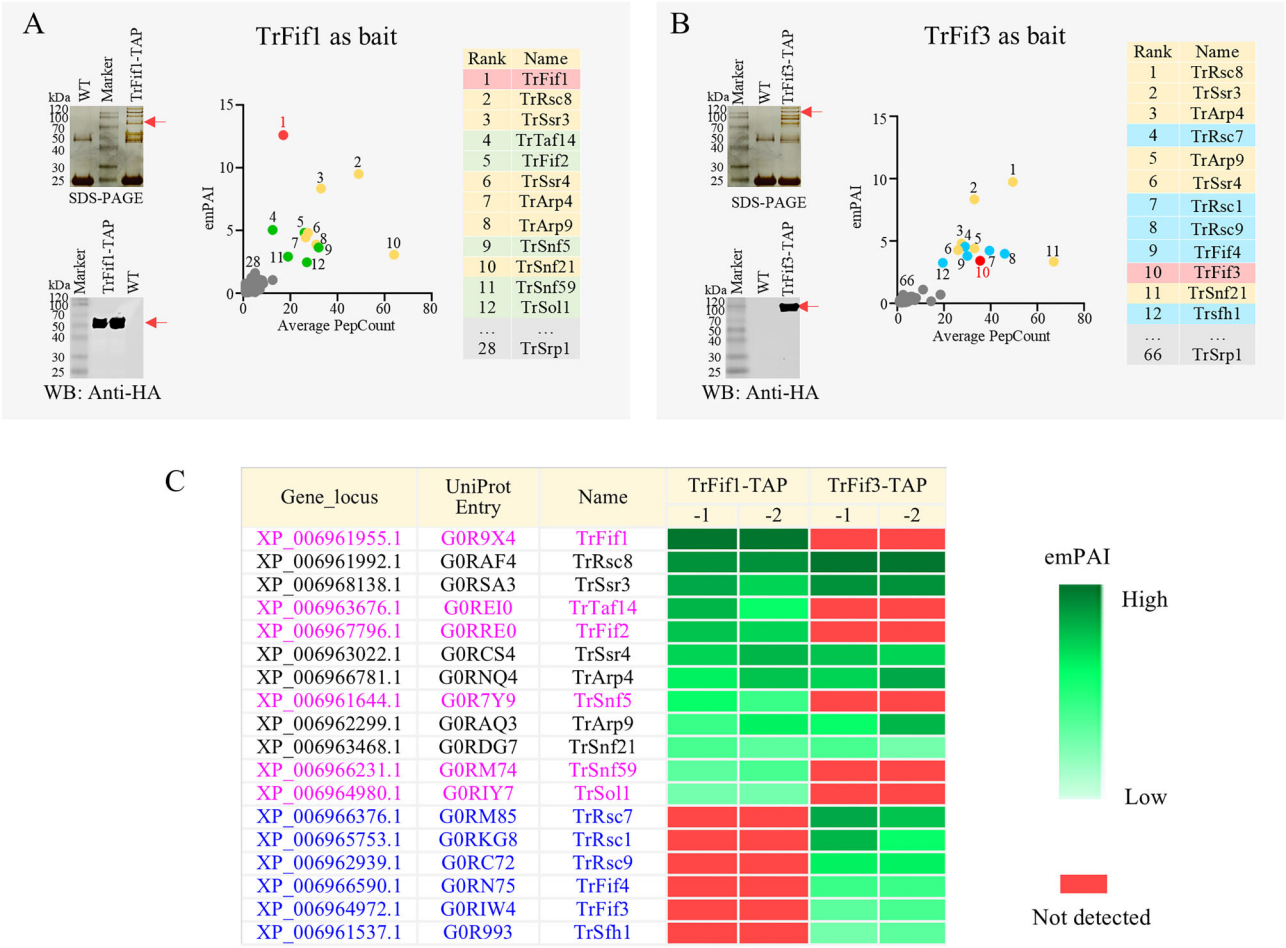
The determination of compositions of SWI/SNF and RSC complexes in *T. reesei*. (**A, B**) SDS–PAGE, WB, and protein rank analysis of TAP using TrFif1 and TrFif3 as the baits, respectively. Red arrows, the bait proteins. The theoretical molecular weights of the baits are 57.3 and 102.5 kDa, respectively. The list of identified proteins was pre-filtered to exclude proteins detected in the control samples and then ranked by emPAI value from highest to lowest. Red dots/backgrounds, the bait proteins. Yellow dots/backgrounds, the shared subunits between SWI/SNF and RSC. Green dots/backgrounds, SWI/SNF-specific subunits. Blue dots/backgrounds, RSC-specific subunits. Gray dots/backgrounds, non-NF-Y or non-CRC proteins, including Importin TrSrp1 (Importin α) and/or TrKap95 (Importin β). (**C**) TAP-MS results from duplicate samples of subunits of the CRCs in *T. reesei*. Black font: the shared subunits between SWI/SNF and RSC. Magenta font: SWI/SNF-specific subunits. Blue font: RSC-specific subunits. The emPAI gradient is represented by a green color scale (dark-green, high value; light-green, low value) for each TAP strain sample. Undetected proteins are marked in red. Not all ranking positions are displayed in the figure. The emPAI values for all samples and complete ranked lists are provided in Supplementary Spreadsheets S5, Sheets 2 and 3.

In *T. reesei*, TAP-MS analyses using TrFif1 and TrFif3 as bait proteins yielded similar results (Fig. 3). When TrFif1 subunit was used as the bait, shared subunits between SWI/SNF and RSC (Fig. 3A, yellow dots/backgrounds), and SWI/SNF-specific subunits including TrFif2 (ranked 5th) (Fig. 3A, green dots/backgrounds) were observed but no RSC-specific subunits were detected.

In contrast, when TrFif3 subunit was used as the bait, shared subunits between SWI/SNF and RSC (Fig. 3B, yellow dots/background) and RSC-specific subunits including TrFif4 (ranked 9th) (Fig. 3B, blue dots/backgrounds), were observed, but no SWI/SNF-specific subunits were detected.

Furthermore, consistent with the observations in *P. oxalicum* PoSnf21-TAP and PoFif1/2/3-TAP (Fig. 2A–D), the heterodimeric Importin subunit TrSrp1 was also identified in TrFif1-TAP and TrFif3-TAP (Fig. 3A and B). This recurrent association suggests that one or more subunits of SWI/SNF and RSC complexes may require nuclear import mediated by a heterodimeric Importin complex for the assembly.

For clarity, duplicate samples of TrFif1-TAP and TrFif3-TAP are presented together. TrFif1 and TrFif2 subunits were consistently copurified, whereas TrFif3 and TrFif4 subunits were consistently copurified. TrSWI/SNF-specific subunits (TrSnf5, TrSnf59, TrSol1, and TrTal14) were exclusively associated with TrFif1 and TrFif2, whereas TrRSC-specific subunits (TrSfh1, TrRsc1, TrRsc7, and TrRsc9) were exclusively associated with TrFif3 and TrFif4 (Fig. 3C).

### The models of SWI/SNF and RSC complexes in filamentous fungi versus yeast and humans

**Figure 4.**
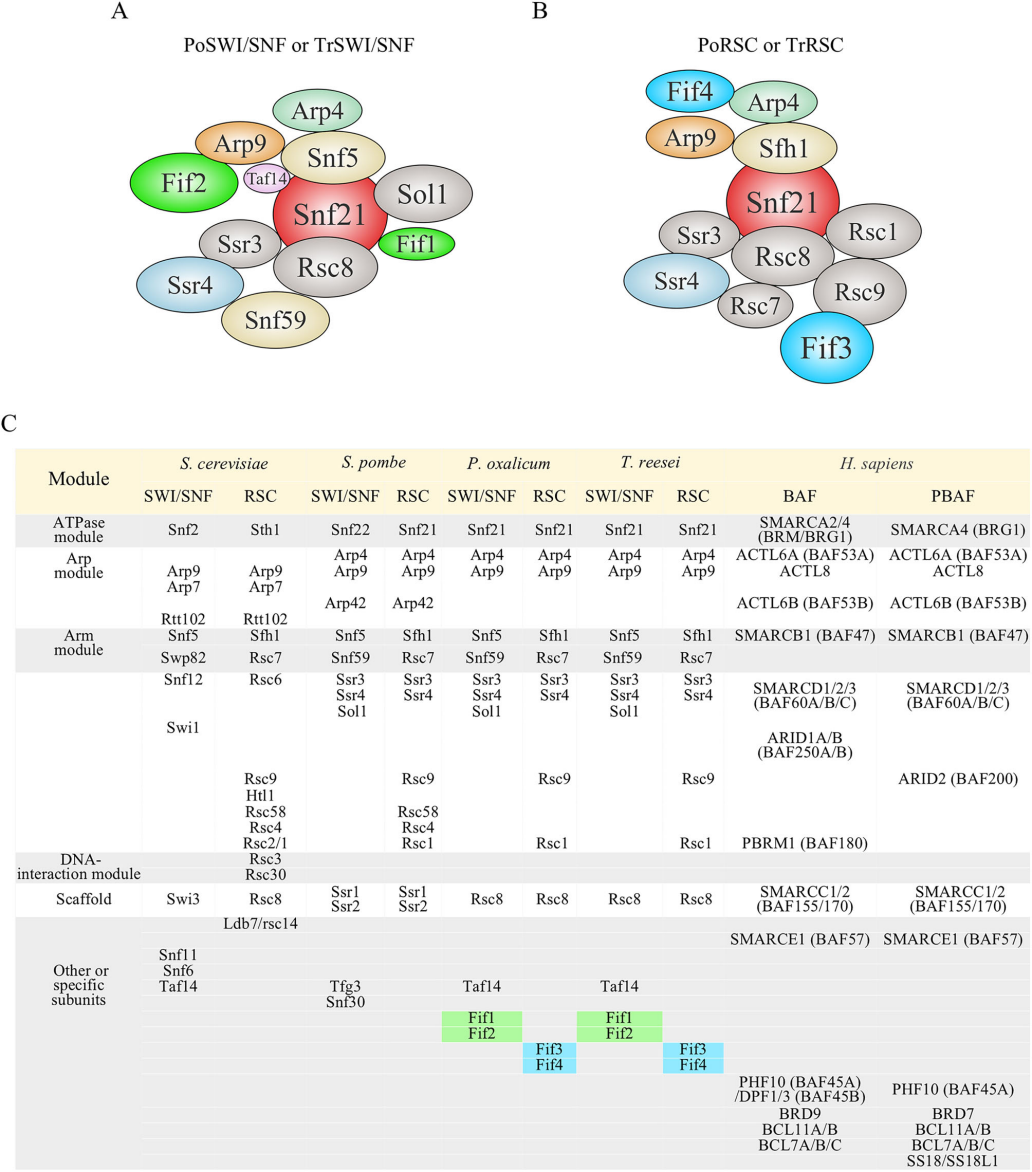
The comparison of SWI/SNF and RSC among different organisms. (**A**) The models of PoSWI/SNF and TrSWI/SNF complexes. (**B**) The models of PoRSC and TrRSC complexes. Subunits of the SWI/SNF and RSC complexes in the two filamentous fungi were named according to their top-scoring orthologs identified by sequence alignment against those in *S. cerevisiae, S. pombe*, and *H. sapiens*. The alignment results are provided in Supplementary Spreadsheet S8. The models were drawn and the positions of non-specific subunits were assigned according to references [20, 21, 46]. The positions of filamentous fungus-specific subunits, Fif1, Fif2, Fif3, and Fif4, were predicted based on the subunits with which they are most likely to form protein–protein interactions, as determined by the highest ipTM + pTM scores. ipTM, interface predicted Template Modeling score.  pTM, predicted Template Modeling score. The prediction was performed using AlphaFold Multimer (https://cosmic-cryoem.org/tools/alphafoldmultimer/) [47]. The scores (ipTM + pTM) for Fif1, Fif2, Fif3, Fif4 and their putative interacting subunits are listed in Supplementary Spreadsheet S9. (**C**) The comparison of SWI/SNF and RSC from budding yeast, fission yeast, *P. oxalicum, T. reesei*, and humans.

Integrating results from Figs 2 and 3 reveals a conserved organization of SWI/SNF and RSC complexes in two filamentous fungi, as well as the differences between the organization in these fungi and that of SWI/SNF and RSC complexes from yeast and humans (Fig. 4).

Both PoSWI/SNF and TrSWI/SNF comprise 12 subunits: six shared subunits (Snf21, Ssr3, Rsc8, Arp4, Arp9, and Ssr4) and six SWI/SNF-specific subunits (Snf5, Snf59, Sol1, Taf14, Fif1, and Fif2) (Fig. 4A).

Both PoRSC and TrRSC also comprise 12 subunits: six shared subunits (Snf21, Ssr3, Rsc8, Arp4, Arp9, and Ssr4) and six RSC-specific subunits (Rsc1, Rsc7, Rsc9, Sfh1, Fif3, and Fif4) (Fig. 4B).

Notably, SWI/SNF-specific Fif1/Fif2 and RSC-specific Fif3/Fif4—unique to filamentous fungi—distinguish filamentous fungi’s SWI/SNF and RSC from their counterparts in unicellular yeast and multicellular animals (Fig. 4C), representing novel adaptations not observed in yeast, plants, or metazoans.

### Identification of binding patterns in NF-Y-CRCs interaction

The NF-Y complex comprises three subunits, while SWI/SNF and RSC complexes typically contain 12 subunits each. The precise interaction mechanisms, specifically determining which NF-Y subunit(s) interact(s) with which component(s) of SWI/SNF and RSC complexes, required elucidation. Y2H strains expressing subunits of PoNF-Y, PoSWI/SNF, and PoRSC complexes were constructed. Following confirmation of absent toxicity and autoactivation in these Y2H strains (Supplementary Fig. S11), we performed pairwise interaction assays to identify subunit–subunit interactions. The results, assessed by colony growth on QDO medium and QDO/X-α-Gal/Aba, revealed distinct interaction patterns (Supplementary Fig. S12A). The results revealed that: (i) The PoSWI/SNF and PoRSC shared subunit PoSsr4 and the SWI/SNF-specific subunit PoSol1 exhibited strong interaction with all three PoNF-Y subunits, as evidenced by robust colony growth on QDO and intense blue staining on QDO/X-α-Gal/Aba; (ii) some subunits displayed weaker or more selective interactions: PoFif1 formed colonies on QDO but showed faint blue staining; PoSnf59 generated consistently smaller colonies on both media; and PoRsc7 interacted strongly with NF-YB but weakly with NF-YA and NF-YC; and (iii) with the exception of the subunits listed earlier, none of the other PoSWI/SNF or PoRSC subunits exhibited interaction with any of the three PoNF-Y subunits. These differential patterns suggest that while interaction with Ssr4 and Sol1 may facilitate general association between NF-Y and CRCs, other subunits likely contribute to fine-tuning through weaker interactions.

### Nuclear localization and constitutive expression of CRC subunits

Given the key transcriptional regulatory roles of SWI/SNF and RSC complexes, these CRCs likely contribute to characteristic fungal adaptations such as filamentous hyphal growth, conidiation, secondary metabolite (SM) biosynthesis, and high-yield production of extracellular cell wall-degrading enzymes (CWDEs), processes regulated mainly through transcriptional control [48, 49]. Critically, Y2H analysis identified Fif1 (SWI/SNF-specific) and Fif3 (RSC-specific) as direct interactors mediating NF-Y-CRC associations. Based on these findings and their presence exclusive in filamentous fungi, we selected the SWI/SNF subunit Fif1 and RSC subunit Fif3 from *P. oxalicum* as representative targets for functional evaluation of their respective complexes.

We investigated their subcellular localization. GFP-tagged localization in VMMG or VMMC revealed nuclear enrichment of PoFif1–Fif3 (Supplementary Fig. S12B–D), consistent with their identities as subunits of SWI/SNF and RSC complexes that function as chromatin remodelers in the nucleus. We next examined expression levels of PoFif1–Fif4 alongside representative CRC subunits, including shared subunits (core ATPase Snf21, ARP module Arp4, body module Rsc8), SWI/SNF-specific Snf5, and RSC-specific Sfh1, across nutrient-rich (PDB) and minimal media conditions: VMMG, VMM with VMMGly, and VMMCW. No significant differences in subunit expression were observed across media (Supplementary Fig. S13), indicating constitutive expression of these CRC genes.

### SWI/SNF-specific PoFif1 and RSC-specific PoFif3 exert opposing regulatory roles on CWDE production

In natural habitats, filamentous fungi predominantly encounter recalcitrant carbon substrates, particularly lignocellulosic plant biomass. To decompose these complex polymers for growth, they have evolved potent CWDE production systems. We therefore investigated the roles of PoFif1 and PoFif3 in CWDE production using wheat bran and microcrystalline cellulose as simulated natural substrates.

**Figure 5.**
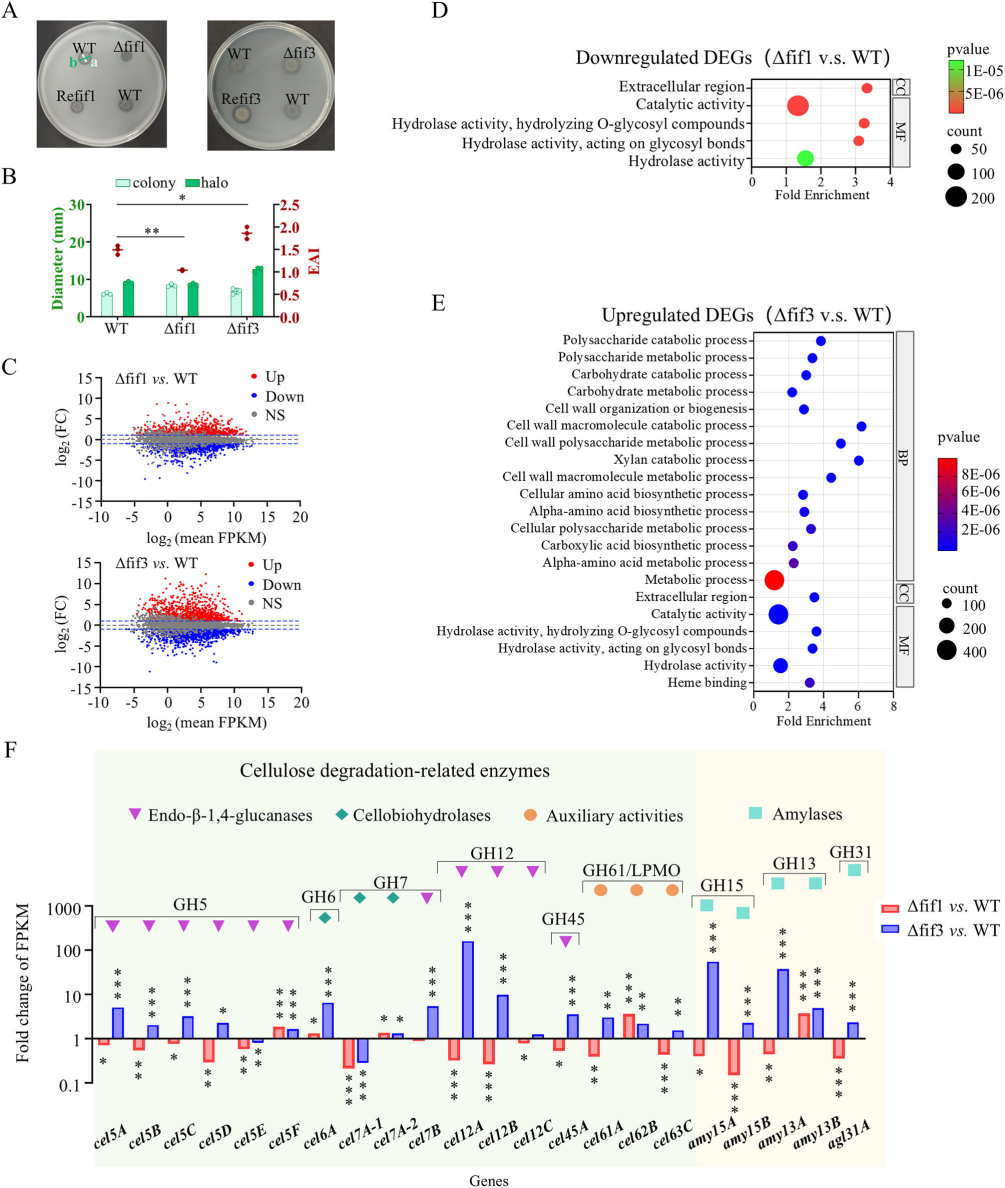
Regulatory roles of Fif1 and Fif3 under recalcitrant carbon sources. (**A**) Colony phenotypes of the WT, Δfif1, Refif1, Δfif3, and Refif3 strains on VMMC agar. Sage-green line (a), colony diameter; dark-green line (b), diameter of the cellulolytic halo. (**B**) EAI of the Δfif1 and Δfif3 mutants. EAI values were calculated as the ratio of the cellulolytic halo diameter (b) to colony diameter (a) from panel (**A**). *P < .05; **P < .01. (**C**) MA plots of Δfif1 versus WT (Top) and Δfif3 versus WT (Bottom). Up: significantly upregulated; Down: significantly downregulated; NS: not significant. (**D**) GO enrichment of downregulated DEGs in Δfif1. (**E**) GO enrichment of upregulated DEGs in Δfif3. The enrichment analysis was conducted in ShinyGO, v.0.82 (https://bioinformatics.sdstate.edu/go/). BP, biological process; CC, cellular component; MF, molecular function. (**F**) Cellulose degradation-related gene (green backgrounds) and amylase gene (yellow backgrounds) expression patterns in Δfif1 and Δfif3 mutants. Bars represent the fold change in gene expression (mutant average FPKM/WT average FPKM). Upward-pointing bars, upregulation. downward-pointing bars, downregulation. *P < .05, **P < .01, ***P < .001.

The gene deletion mutants (Δfif1 and Δfif3) and their respective complemented strains (Refif1 and Refif3) were generated. The phenotypes of the mutants grown on VMMC medium, together with the transcriptome data, are presented in Fig. 5. When cultured on VMMC agar medium, the Δfif3 mutant exhibited significantly enhanced radial colony expansion compared to the WT strain. Δfif3 colonies were encircled by an expanded cellulolytic halo, suggesting enhanced cellulolytic enzyme secretion and improved cellulose degradation activity (Fig. 5A, right plate). Both Refif1 and Refif3 behaved similarly to the WT strain, displaying no obvious cellulolytic halo (Fig. 5A). Quantitative analysis revealed distinct cellulolytic halo-to-colony diameter ratios. This ratio is commonly defined as the enzymatic activity index (EAI) to measure the efficiency of fungal extracellular enzyme production [50]. The EAIs for WT, Δfif1, and Δfif3 were 1.49 ± 0.10, 1.03 ± 0.01, and 1.86 ± 0.13, respectively. The EAI of Δfif1 was significantly lower than that of WT, while the EAI of Δfif3 was significantly higher than that of WT (Fig. 5B). These observations indicate that PoFif3 absence may result in overproduction and hypersecretion of cellulolytic enzymes that consequently improve cellulose utilization efficiency in the mutant. Conversely, absence of PoFif1 appears to exert opposite regulatory effects on cellulolytic enzyme production.

Next, we conducted transcriptome analysis in Δfif1 and Δfif3 strains cultured in VMMC liquid medium. Analysis of differential gene expression showed that, compared to the WT strain, the Δfif1 mutant showed 1440 DEGs (754 up, 696 down), as shown in the MA plot (Fig. 5C, top). The Δfif3 strain showed 2 463 DEGs (1200 up, 1 263 down) (Fig. 5C, bottom). Detailed information on DEGs is provided in Supplementary Spreadsheet S10. The larger number of DEGs in Δfif3 compared to Δfif1 indicates that deletion of PoFif3 has a greater transcriptional impact than deletion of PoFif1. These DEGs were then subjected to GO enrichment analysis. The results of GO analysis revealed contrasting patterns between mutants. In Δfif1, downregulated genes yielded significant GO enrichment (Fig. 5D), while upregulated genes showed no enrichment. Conversely, in Δfif3, upregulated genes displayed significant enrichment (Fig. 5E), whereas downregulated genes did not.

GO enrichment analysis of upregulated genes in the Δfif3 mutant revealed predominant associations with BPs related to cell wall component metabolism, including “polysaccharide metabolic process,” “carbohydrate metabolic process,” “cell wall macromolecule catabolic process,” and “xylan catabolic process” (Fig. 5E). Notably, carbohydrates such as cellulose and xylan are major constituents of the plant cell wall. The sole enriched CC term was “extracellular region,” exactly the localization where CWDEs are secreted and enriched. MF terms included “hydrolase activity, hydrolyzing O-glycosyl compounds” where a major component of O-glycosyl compounds is precisely cell wall polysaccharides—represented by cellulose, hemicellulose, and pectin. These findings collectively suggest that PoFif3 absence promotes upregulation of CWDE-encoding genes.

Conversely, while downregulated genes in Δfif1 showed no significant BP term enrichment, they demonstrated enrichment in both the “extracellular region” (CC) and “hydrolase activity, hydrolyzing O-glycosyl compounds” (MF) categories (Fig. 5D). These identical terms appeared in enrichment results for downregulated genes in Δfif1 versus upregulated genes in Δfif3 (Fig. 5E), indicating opposing regulatory roles for Fif1 and Fif3 in modulating CWDE production-related functions.

In addition, Δfif3 upregulated genes were also enriched for “heme binding” (MF), primarily represented by cytochrome P450 genes. Given the established role of cytochrome P450 enzymes in fungal SM biosynthesis, this observation suggests PoFif3 may functionally participate in secondary metabolic pathways.

Then, we conducted detailed analysis of CWDE gene expression profiles in Δfif1 and Δfif3 mutants. Based on structurally conserved catalytic and carbohydrate-binding modules, *P. oxalicum* is predicted to encode 114 CWDEs [51]. These enzymes comprise five major classes: cellulolytic enzymes, β-glucosidases, hemicellulases, pectinases, expansin-like proteins, and CAZy-unassigned plant CWDEs. PoFif1 and PoFif3 exhibited differential regulatory effects on most CWDE-encoding genes. In the Δfif1 mutant, the majority (89/114 genes) of CWDE genes were downregulated, whereas Δfif3 showed predominant upregulation (80/114 genes) of CWDE genes (Supplementary Fig. S14).

For example, among 17 cellulose-degrading enzymes—including 14 cellulases (11 endo-β-1,4-glucanases and 3 cellobiohydrolases) classified into glycoside hydrolase (GH) families 5, 6, 7, 12, and 45, along with 3 lytic polysaccharide monooxygenases (LPMOs; formerly GH61)—13 genes were downregulated in Δfif1, while 15 were upregulated in Δfif3 (Fig. 5F, green backgrounds). Furthermore, we analyzed amylase gene expression, as filamentous fungi frequently encounter starch-containing plant seeds in nature and have evolved robust amylase-producing capacity, although amylases are not classified as CWDEs. Amylase genes exhibited a regulatory pattern similar to that of CWDEs: all five were upregulated in Δfif3, while four were downregulated in Δfif1 (Fig. 5F, yellow backgrounds). qPCR analysis of the major cellulase genes *cel7A-1* and *cel5A* was performed. Compared with the WT, *cbh1* expression was significantly downregulated in Δfif1. In contrast, both *cel7A-1* and *cel5A* were significantly upregulated in Δfif3 (Supplementary Fig. S15A). These findings demonstrate that absence of PoFif1 and PoFif3 triggers opposing transcriptional responses in most CWDE genes and thus indicates their divergent regulatory roles in CWDE production.

### Divergent and shared regulatory roles of PoFif1 and PoFif3 in response to glucose conditions

Unlike natural environments where filamentous fungi primarily utilize complex lignocellulosic biomass as carbon sources, laboratory studies and industrial applications typically employ readily metabolized substrates such as glucose. We therefore investigated the regulatory roles of the PoFif1 and PoFif3 subunits under glucose conditions. Transcriptome data of all 12 cellulase genes and 3 LPMO genes revealed that, compared to the housekeeping gene *actin*, which had FPKM values ranging from 1500 to 2000 in the WT, Δfif1, and Δfif3 strains, most cellulase genes showed very low FPKM values. Notably, the key cellulases, including the cellobiohydrolases *cel7A-1* and *cel6A* as well as the β–1,4–endoglucanases *cel5A* and *cel7B*, displayed FPKM values below 10, indicating strong repression (Supplementary Fig. S16). The results are likely attributable to carbon catabolite repression (CCR) [52, 53]. Therefore, our analysis focused primarily on the roles of PoFif1 and PoFif3 in fungal growth and development.

**Figure 6.**
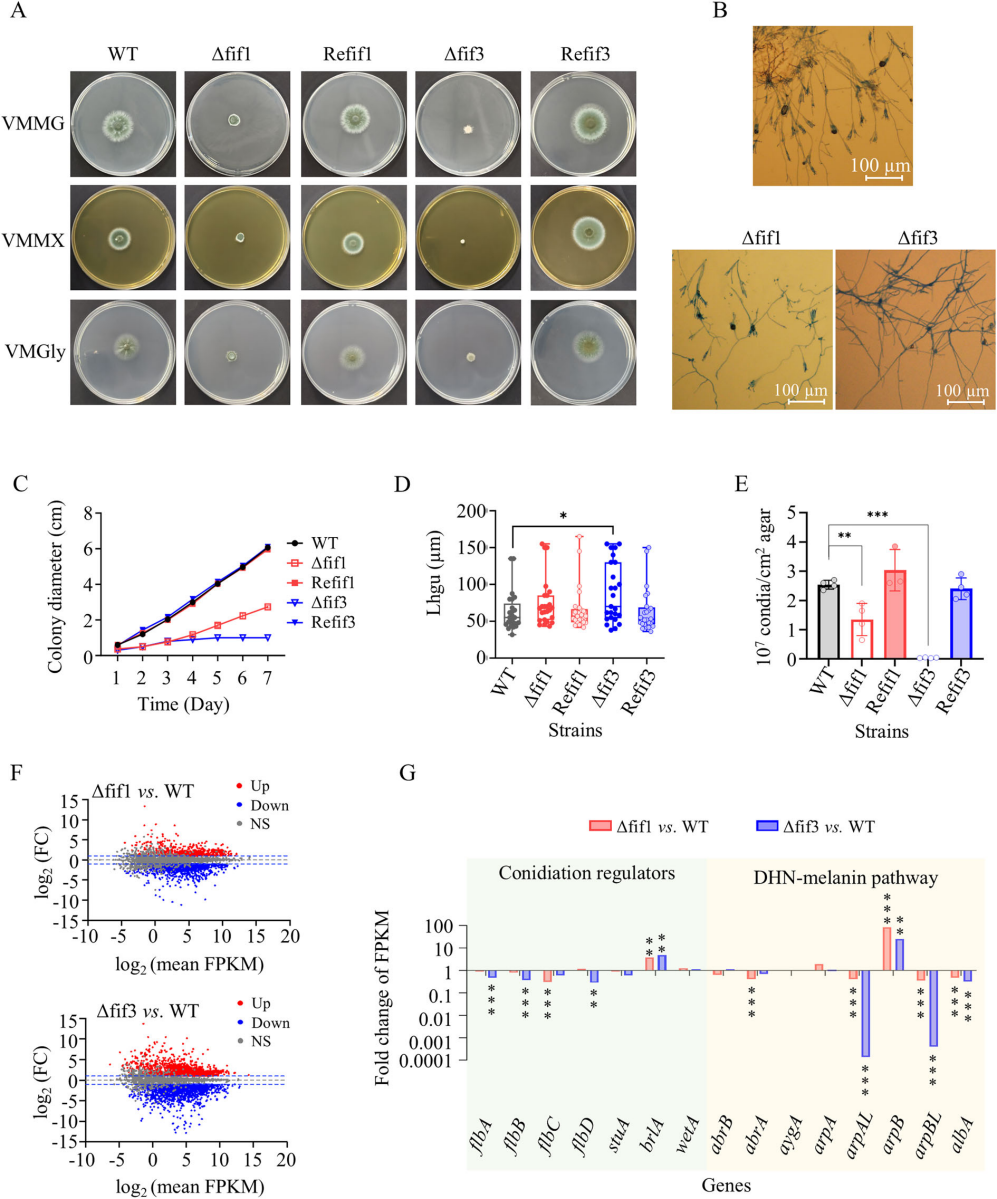
Effects of Fif1 and Fif3 on fungal growth and asexual development. (**A**) Colony morphology. (**B**) Conidiophore observation. (**C**) Measurement of colony radial extension rate. (**D**) Determination of hyphal growth unit length. (**E**) Conidia quantification. (**F**) MA plots of Δfif1 versus WT (Top) and Δfif3 versus WT (Bottom). Up: significantly upregulated; Down: significantly downregulated; NS: not significant. (**G**) Conidiation regulator gene (green background) and DHN-melanin pathway gene (yellow background) expression patterns in Δfif1 and Δfif3 mutants. Bars represent the fold change in gene expression (mutant average FPKM/WT average FPKM). Upward-pointing bars, upregulation. downward-pointing bars, downregulation. *P < .05, **P < .01, ***P < .001.

Effects of PoFif1 and PoFif3 on fungal growth, conidiation, and expression of conidiation and melanin pathway genes are shown in Fig. 6. When cultured on media VMMG, VMMX, and VMMGly, both Δfif1 and Δfif3 mutants exhibited significantly impaired radial growth, evidenced by substantially smaller colony diameters compared to the WT strain, whereas the Refif1 and Refif3 strains showed colony sizes comparable to that of the WT (Fig. 6A). Quantitative analysis of fungal radial growth rate (*Ē*) on VMMG agar revealed the WT strain grew at 385.9 ± 13.2 µm/h, while mutants showed significant reductions: Δfif1 at 171.1 ± 13.9 µm/h (55.7% reduction) and Δfif3 at 49.1 ± 9.9 µm/h (82.7% reduction) (Fig. 6C). Hyphal growth unit length (*L*_hgu_ or *G*) measurements showed values of 62.6 µm for WT, 74.9 µm for Δfif1, and 88.1 µm for Δfif3 (Fig. 6D). While Δfif1 showed no statistically significant difference in *L*_hgu_ compared to WT, Δfif3 exhibited a significant increase, indicative of reduced *L*_hgu_. Applying the equation *Ē* = µ_max_  *G*, maximum specific growth rates (µmax) were calculated as 4.57/h for WT, 2.28/h for Δfif1, and 0.56/h for Δfif3. The Refif1 and Refif3 strains did not differ significantly from the WT strain in both fungal radial growth rate and L_hgu_ (Fig. 6C and D). These results demonstrate that deletion of both PoFif1 and PoFif3 impairs fungal growth, with PoFif3 absence exerting stronger inhibitory effects than PoFif1 absence.

The Δfif1 and Δfif3 mutants exhibited not only reduced fungal growth but also significantly lighter pigmentation compared to the WT strain. Generally, two factors contribute to lightened colony color: decreased conidia production and/or impaired pigment synthesis in conidia. Conidia production in the Δfif1 and Δfif3 mutants on VMMG was quantified, revealing reductions to 72 ± 4% and 5 ± 0.1% of the WT levels, respectively. In contrast, the Refif1 and Refif3 strains exhibited conidia production comparable to that of the WT strain, with no significant difference (Fig. 6E). At 22 h post-inoculation (hpi), broom-like conidiophores were observed in the WT strain, indicating conidiation initiation. In contrast, the Δfif1 mutant displayed sporadic initiation of conidiophore development, while the Δfif3 mutant exhibited complete absence of conidiophore formation (Supplementary Fig. S17A). By 28 hpi, WT produced dense conidiophores, Δfif1 formed sparse conidiophores at significantly reduced density, and Δfif3 remained entirely aconidiate (Fig. 6B).

Next, we conducted transcriptome analysis in Δfif1 and Δfif3 strains cultured in VMMG liquid medium. Analysis of differential gene expression showed that, compared to the WT strain, the Δfif1 mutant had 2290 DEGs (1061 up, 1229 down), as shown in the MA plot (Fig. 6F, top). The Δfif3 strain showed 3 598 DEGs (1580 up, 2018 down) (Fig. 6F, bottom). Detailed information on DEGs is provided in Supplementary Spreadsheet S11. These DEGs were then subjected to GO enrichment analysis.

GO enrichment analysis of DEGs revealed distinct functional divergences between Δfif1 and Δfif3 mutants (Supplementary Fig. S18). Upregulated DEGs in Δfif1 were broadly enriched in terms associated with small molecule metabolism, such as “carboxylic acid metabolic process,” “purine ribonucleotide biosynthetic process,” and “alpha-amino acid metabolic process” (Supplementary Fig. S18A). In contrast, upregulated DEGs in Δfif3 exclusively enriched to nuclear chromosome events such as “DNA replication,” “DNA repair,” and “DNA recombination” (Supplementary Fig. S18C). Downregulated DEGs in Δfif1 predominantly associated with “carbohydrate metabolic processes” and “hydrolase activity hydrolyzing O-glycosyl compounds” (Supplementary Fig. S18B).

Notably, these “carbohydrate metabolic processes” and “hydrolase activity hydrolyzing O-glycosyl compounds” terms were consistent with the enriched GO terms observed for downregulated DEGs in Δfif1 under lignocellulosic conditions (Fig. 5D). Within the latter term, 40% of annotated genes encoded CWDEs, indicating a conserved disruption in CWDE gene expression in Δfif1 under both conditions. Downregulated DEGs in Δfif3 showed significant enrichment in “oxidation-reduction process” and “oxidoreductase activity” (Supplementary Fig. S18D). These results showed divergent regulatory roles for PoFif1 and PoFif3 in coordinating metabolic pathways and genome integrity mechanisms.

Analysis of key conidiation regulator gene expression (Fig. 6G, green backgrounds) revealed significant downregulation of *flbC* in the Δfif1 mutant and significant downregulation of *flbA, flbB*, and *flbD* in the Δfif3. Fungi commonly produce characteristic pigments, with melanin, a key component of spore walls being synthesized through the dihydroxynaphthalene (DHN)-melanin pathway. This pathway comprises six sequentially acting genes: *abrB*/*yA*→*abrA*→*aygA*→*arpA*→*arpB*→*albA*/*wA* [54]. Among these, *abrB* encodes a laccase; *abrA* encodes a pigment biosynthesis protein brown 1; *albA* encodes a polyketide synthase (PKS); while *arpA* and *arpB* encode tetrahydroxy-naphthalene reductases. Transcriptome data revealed that both Δfif1 and Δfif3 mutants exhibited significant downregulation of *arpAL* and *arpBL* (paralogs of *arpA* and *arpB*, respectively), and *albA* compared to the WT (Fig. 6G, yellow backgrounds). qPCR analysis was performed on two early genes of the DHN-melanin pathway, *abrB* and *abrA*. Compared with the WT, the expression of both genes was downregulated in the Δfif1 and Δfif3 mutants (Supplementary Fig. S15B). These findings suggest that the whitening of Δfif1 or Δfif3 colonies results from the combined effects of reduced conidia and impaired melanin synthesis.

### Carbon source-dependent regulation of biosynthetic gene clusters by SWI/SNF and RSC complexes

**Figure 7.**
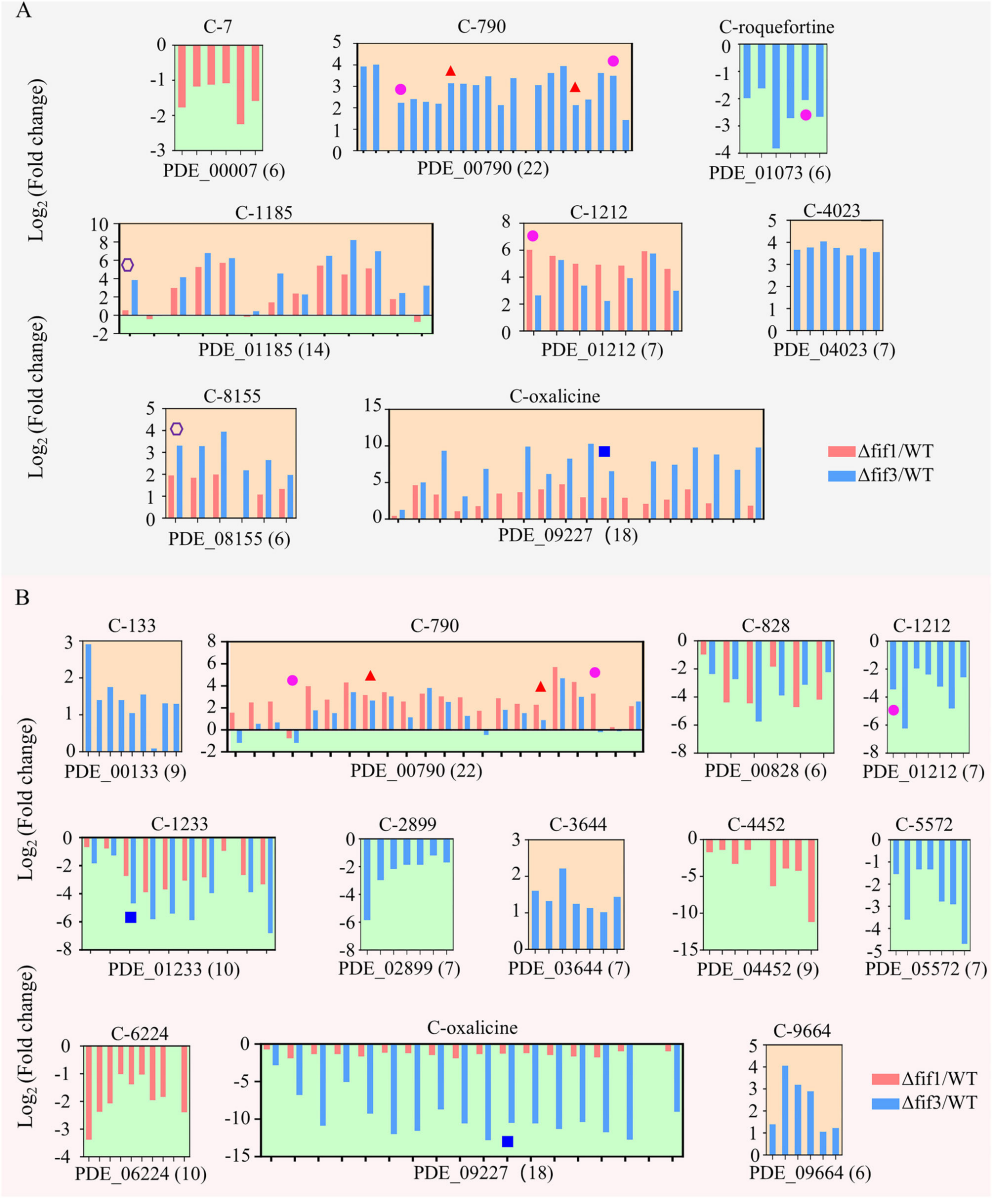
Effects of PoFif1 and PoFif3 on co-regulated gene clusters. (**A**) Clusters identified under lignocellulosic conditions. (**B**) Clusters identified under glucose conditions. The two clusters with known products were designated as C-roquefortine and C-oxalicine, respectively. The clusters of unknown products were named according to the number of their first gene: e.g. a cluster whose first gene is PDE_00 007 was named C-7, and one with PDE_00 790 was named C-790. Co-regulated gene clusters were defined as physically linked genomic regions meeting: ≥6 contiguous genes with |log_2_(FoldChange)| ≥ 1, or ≥ 8 genes (allowing one gap) with |log_2_(FoldChange)| ≥ 1. Bars represent the log_2_(fold change) in gene expression (mutant average FPKM/WT average FPKM). Upward-pointing bars, upregulation. downward-pointing bars, downregulation. Orange background: genes with upregulated expression; green background: genes with downregulated expression. The gene locus tag below the *x*-axis indicates the first gene in each cluster, followed by the total number of contiguous genes in parentheses. For example: PDE_00 007 (7) in subpanel denotes a cluster spanning seven consecutive genes from PDE_00 007 to PDE_00013. Core backbone biosynthetic genes in each BGC are denoted: blue solid square, PKS gene; magenta solid circle: nonribosomal peptide synthetase (NRPS) gene; red solid triangle: dimethylallyltryptophan synthase gene; purple hollow hexagon: NRPS-like gene.

Beyond substantial extracellular enzyme production such as CWDEs, filamentous fungi also exhibit capacity for synthesizing diverse SMs. These SMs are frequently associated with SM biosynthetic gene clusters (BGCs), wherein physically linked genes are co-regulated to enable coordinated expression of specialized metabolic pathways. Co-regulated gene clusters affected by PoFif1 and PoFif3 under lignocellulosic and glucose conditions are presented in Fig. 7.

Under lignocellulosic conditions, physically linked co-regulated gene clusters were identified in both Δfif1 and Δfif3 mutants (Fig. 7A). Eight clusters comprising 6, 22, 6, 14, 7, 7, 6, and 18 genes, respectively, were detected. Among these clusters, four showed mutant-specific regulation: Cluster C-7 upregulated exclusively in Δfif1, and Clusters C-790 (upregulated), C-roquefortine (downregulated), and C-4023 (upregulated) exclusively in Δfif3. The other four clusters were upregulated in both mutants (Fig. 7A). There are 28 predicted BGCs in *P. oxalicum* (Supplementary Table S1) [30]. Significantly, seven clusters (all except Cluster C-7) overlapped either completely or partially with 7 of the 28 predicted BGCs. These included Cluster C-roquefortine associated with roquefortine C and meleagrin biosynthesis and Cluster C-oxalicine associated with oxalicine B biosynthesis [55, 56]. In summary, all four BGCs (C-1185, C1212, C-8155, and C-oxalicine) affected by PoFif1 absence were upregulated, and 6 (C-790, C-1185, C1212, C4023, C-8155, and C-oxalicine) of 7 BGCs affected by PoFif3 absence were also upregulated, collectively suggesting that both PoFif1 and PoFif3 likely function as negative regulators of BGC expression (Fig. 7A).

Under glucose conditions, 12 co-regulated gene clusters containing 9, 22, 6, 7, 10, 7, 7, 9, 7, 10, 18, and 6 genes, respectively, were identified (Fig. 7B). Four clusters (C-790, C-828, C-1233, C-oxalicine) showed consistent regulation in both mutants, with C-790 upregulated and C-828, C-1233, C-oxalicine downregulated in both Δfif1 and Δfif3, while the remaining eight clusters exhibited mutant-specific regulation. Four glucose-responsive clusters (C-790, C-1212, C-1233, C-oxalicine) overlapped either completely or partially with predicted SM clusters.

Notably, three clusters (C-790, C1212, and C-oxalicine) are both cellulose-responsive and glucose-responsive. All three are BGCs but displayed divergent regulatory patterns: Under cellulose, the C-790 was upregulated only in Δfif3, whereas under glucose, it was upregulated in both mutants; the C-1212 was upregulated in both mutants under cellulose but downregulated in Δfif3 under glucose; and the C-oxalicine was upregulated in both mutants under cellulose but downregulated in both mutants under glucose. These findings demonstrate complex carbon source-dependent regulation of BGCs by PoFif1 and PoFif3.

### Disruption of either PoNF-YB or PoNF-YC impairs fungal growth and CWDE expression while dysregulating core biosynthetic genes

**Figure 8.**
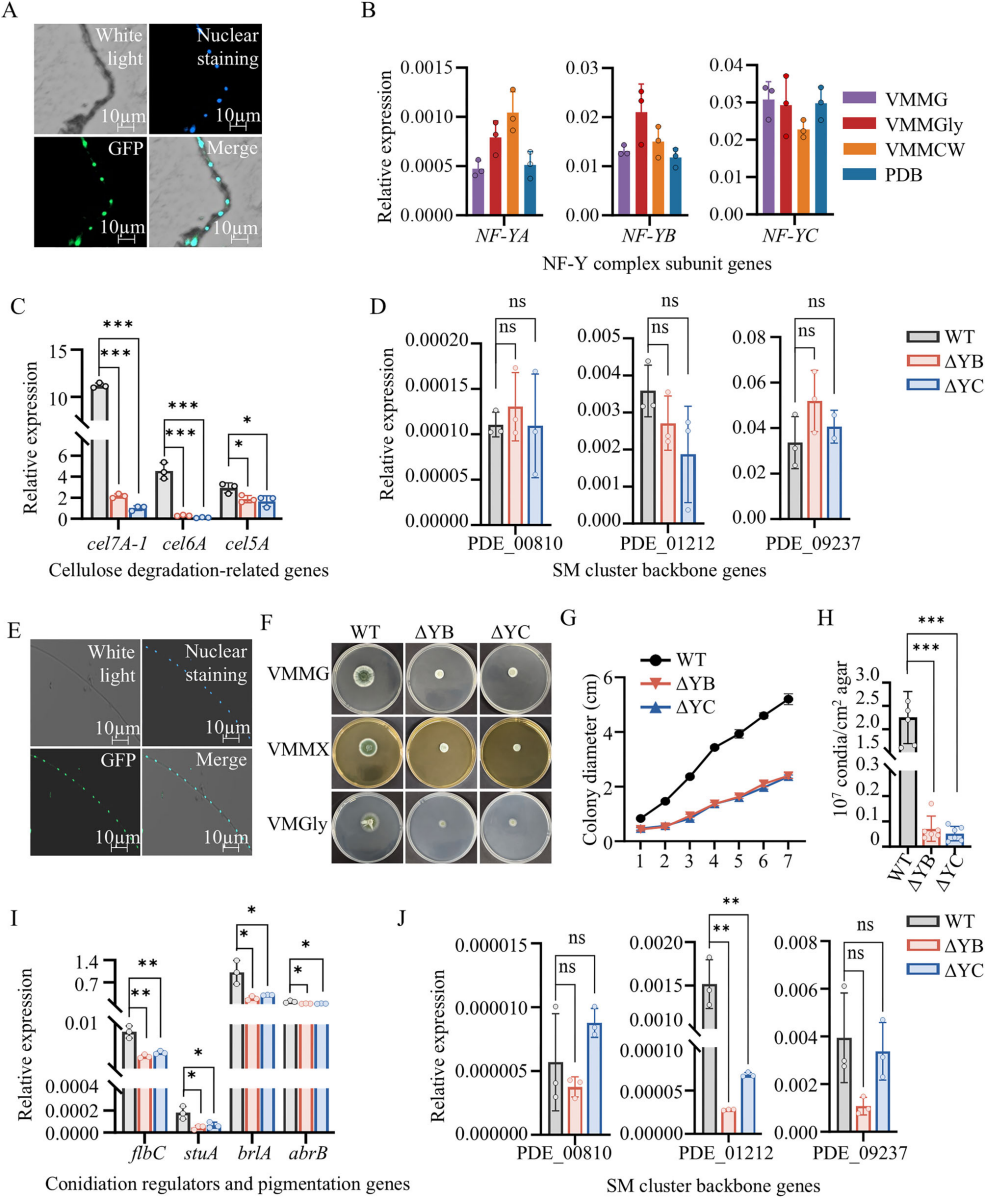
Phenotypic and transcriptional characterization of the ΔYB and ΔYC. Nuclear localization of NF-YC subunit under lignocellulosic (**A**) and glucose (**E**) conditions, respectively. Each image is divided into four parts: upper left, white light; upper right, Hoechst 33 342 was used to stain nuclei in blue; bottom left, green fluorescence; bottom right, merged image of green fluorescence and nuclear staining. Transcription level of the NF-Y subunit genes (**B**), cellulose-degrading-related genes (**C**), and SM core biosynthetic genes (**D**) assayed by qRT-PCR. (**F**) Colony phenotypes of the WT, ΔYB, and ΔYC on VMMG, VMMX, and VMMGly agar plate. (**G**) Measurement of colony radial extension rate. (**H**) Conidia quantification. Transcription level of the development-related genes (**I**) and SM core biosynthetic genes (**J**) assayed by qRT-PCR. All qRT-PCR values (B–D, I–J) were normalized to *actin* transcript levels set to 1. Error bars represent SD; asterisks indicate statistical significance (*P < .05, **P < .01, ***P < .001).

Building on the established roles of PoFif1/Fif3 in modulating CWDE gene expression (Fig. 5), fungal development (Fig. 6), and SM BGC activation/repression (Fig. 7), we sought to investigate whether the PoNF-Y complex also regulates these processes. We therefore constructed knockout mutants for each of the two PoNF-Y subunits, PoNF-YB and PoNF-YC, in *P. oxalicum* (designated ΔYB and ΔYC) and examined their respective functions. The phenotypes of ΔYB and ΔYC under lignocellulosic and glucose conditions are presented in Fig. 8.

All three PoNF-Y subunits (PoNF-YA, PoNF-YB, and PoNF-YC) exhibited constitutive expression patterns on four tested media (VMMG, VMMC, VMMGly, and PDB) with no significant variation (Fig. 8B). The nuclear localization of PoNF-YC under both lignocellulosic and glucose conditions was also observed (Fig. 8A and E). The constitutive expression of PoNF-Y subunits and nuclear localization are consistent with those observed for PoSWI/SNF and PoRSC subunits (Supplementary Fig. S13).

Under lignocellulose-induced conditions, we analyzed the transcription levels of three key cellulase genes, including two cellobiohydrolase genes (*cel7A-1* and *cel6A*) and one endo-β–1,4–glucanase gene (*cel5A*). These genes were selected because their encoded products represent the most abundant exocellulase and endocellulase, respectively. All three genes showed significant downregulation in the ΔYC and ΔYB strains (Fig. 8C). We also selected three core backbone biosynthetic genes (NRPS gene PDE_00 810, NRPS gene PDE_01 212, and PKS PDE_09 237) from BGCs altered in Δfif1 and Δfif3 mutants under lignocellulosic conditions (located in Clusters C-790, C-1212, and C-oxalicine respectively; Fig. 7A) and quantified their transcript levels. All three genes showed no significant changes in ΔYB or ΔYC (Fig. 8D). This indicates that the cluster-specific alterations observed in Fig. 7A are likely attributable specifically to PoFif1 or PoFif3 absence rather than NF-Y dysfunction.

Under glucose conditions, both the ΔYB and ΔYC strains exhibited smaller colonies (Fig. 8F), reduced radial growth rates (137.9 ± 8.6 µm/h and 144.8 ± 7.3 µm/h, respectively), which corresponded to only 44.3% and 45.1% of the WT rate (Fig. 8G), impaired conidiophore development (Supplementary Fig. S17B), and a significant decrease in conidiation, with yields accounting for only 3.1% and 2.3% of the WT level, respectively (Fig. 8H). These phenotypes align with those observed in the Δfif1 and Δfif3 mutants (Fig. 6A–C and E). Expression levels of key conidiation regulator genes (*flbC, stuA, brlA*) and first (*abrB*) genes in the DHN-melanin pathway were measured. All genes showed significant downregulation (Fig. 8I). We also selected the core biosynthetic genes (NRPS gene PDE_00 810 and PDE_01 212, and PKS gene PDE_09 237) from three BGCs that showed significant changes in the Δfif1 and/or Δfif3 mutants under glucose conditions (located in Clusters C-790, C-1212, and C-oxalicine respectively; Fig. 7B) and measured their expression. The expression levels of PDE_00 810 and PDE_09 237 remained unchanged in the ΔYB and ΔYC strains, whereas they were upregulated in Δfif1 and downregulated in Δfif3 mutants, respectively. Only PDE_01 212 was downregulated in both ΔYB and ΔYC, recapitulated the expression pattern observed in the Δfif3 mutant (Fig. 8J).

In summary, ΔYB and ΔYC exhibits phenotypes similar to Δfif1 and Δfif3, including reduced radial growth and impaired conidiophores and conidiation. However, despite these similarities, specific phenotypic differences exist. For instance, the expression of CWDE genes is downregulated in ΔYB and ΔYC, consistent with the pattern observed in Δfif1, but conversely upregulated in Δfif3. The up- or downregulation patterns of core biosynthetic genes in ΔYB and ΔYC differ from those observed in Δfif1 and/or Δfif3. These findings show the functional complexity and partial divergence between NF-Y and the PoFif1 and PoFif3 subunits.

### PoFif1 and PoFif3 show NF-Y-dependent enrichment at specific promoters

**Figure 9.**
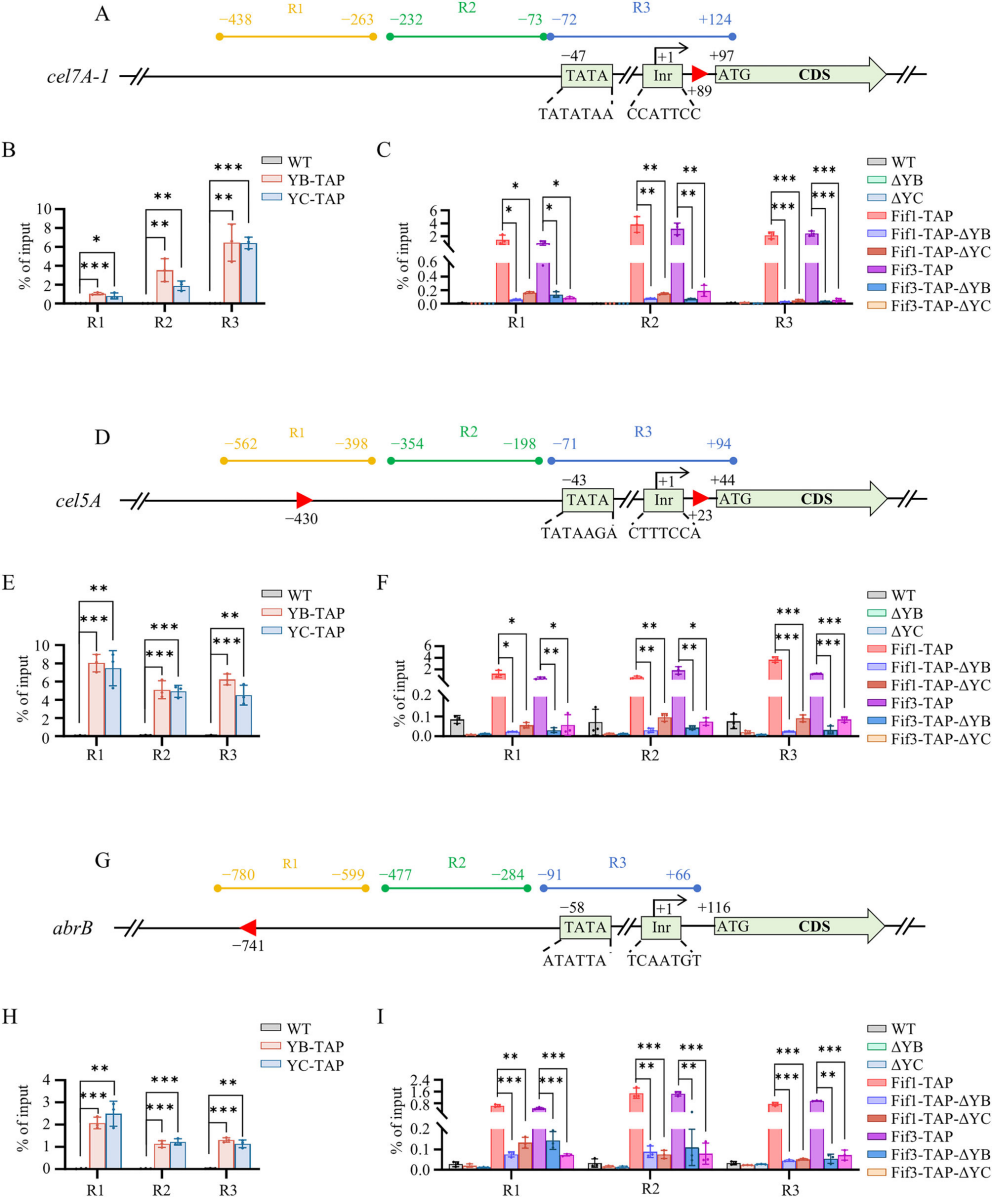
Enrichment of PoNF–YB, PoNF–YC, PoFif1, and PoFif3 at specific loci of target genes. Schematic diagrams of the ChIP-qPCR experimental strategy for the cellulase genes *cbh1* (**A**) and *eg2* (**D**), and the developmental regulatory gene *abrB* (**G**). The TSS is marked as +1. The initiator (Inr) and TATA box are indicated. Three specific regions (R1–R3) within each gene were designed for qPCR analysis. Red triangles denote the putative CCAAT box for NF-Y, with triangle orientation reflecting the direction of the binding motif. ChIP–qPCR results of enrichment of PoNF–YB and PoNF–YC at the indicated regions of *cbh1* (**B**), *eg2* (**E**), and *abrB* (**H**). ChIP–qPCR results of enrichment of PoFif1 and PoFif3 at the indicated regions of *cbh1* (**C**), *eg2* (**F**), and *abrB* (**I**). All values represent the mean of three biological replicates; error bars indicate standard deviation. Significance is marked as follows: *P < .05, **P < .01, ***P < .001.

ChIP-qPCR assays were performed to determine whether PoFif1 and PoFif3 are enriched at the promoters of key cellulase genes (*cel7A-1* and *cel5A*) and the first gene of the development-related DHN-melanin pathway (*abrB*) (Fig. 9).

First, we investigated whether the two NF–Y subunits, NF–YB and NF–YC, bind to the promoter regions of these three genes. The assays were conducted in the WT, YB-TAP, and YC-TAP strains. Strains were cultured in VMMC medium for two CWDE gene assays and in VMMG medium for developmental gene assays. The schematic design is shown in Fig. 9A, D, and G. For each gene, three representative regions (designated R1–R3) were analyzed. R1 and R2 are located upstream of the core promoter, while R3 spans the core promoter itself, encompassing the TATA box and the initiator element (Inr). The results demonstrated that both the NF–YB and NF–YC subunits were enriched across all three regions of each of the three genes examined (Fig. 9B, E, and H), indicating that NF–Y binds to these promoter regions.

We next investigated whether PoFif1 and PoFif3 also show enrichment at these promoter regions (Fig. 9C, F, and I). The assays were conducted in the following strains: WT, ΔYB, ΔYC, Fif1-TAP, Fif3-TAP, as well as strains derived from Fif1-TAP and Fif3-TAP by further deletion of NF–YB (Fif1-TAP–ΔYB; Fif3-TAP–ΔYB) or NF–YC (Fif1-TAP–ΔYC; Fif3-TAP–ΔYC). The results showed that PoFif1 and PoFif3 were enriched across all three regions of the three genes examined. However, this enrichment was substantially reduced upon deletion of either NF-YB or NF-YC, indicating that the promoter occupancy of both PoFif1 and PoFif3 is dependent on the NF-Y complex.

Our observation that PoFif1 enrichment at these promoters depends on NF-Y was expected, given that deletion of either PoFif1 or PoNF-Y subunits similarly results in the downregulation of cellulase genes and defects in fungal growth and development. However, the finding that PoFif3 enrichment at the two cellulase genes is also NF-Y-dependent was unexpected. This is because, as supported by both our data and previous reports [57–60], NF-Y functions as a positive regulator of cellulase expression with its deletion causing reduced cellulase gene expression, whereas loss of PoFif3 leads to the upregulation of cellulase genes. This suggests that PoFif3 may antagonize NF-Y function at these promoters.

## Discussion

**Figure 10.**
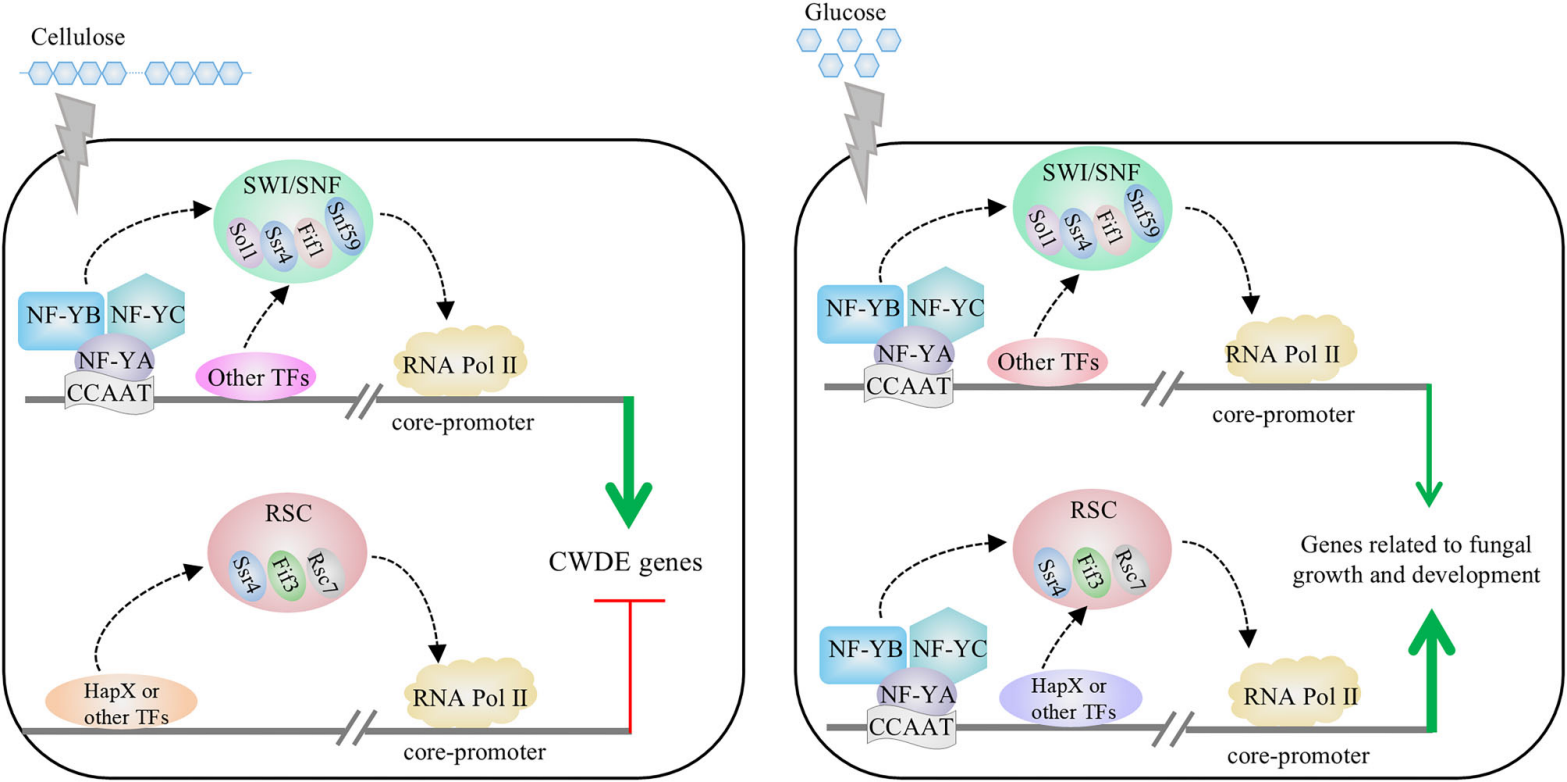
The model of cooperative regulation of gene expression by NF–Y complex and CRCs. Left, under lignocellulosic conditions; right, under glucose condistions. For detailed descriptions, see the main text.

Based on prior studies of the TF NF-Y, SWI/SNF, and RSC complexes by other researchers, as well as findings from this work, a model in which the NF-Y complex interacts with the SWI/SNF and RSC complexes to coordinately regulate target gene expression is proposed (Fig. 10). The NF-Y complex binds the CCAAT-box in the promoters of its target genes. The SWI/SNF and RSC, imported into the nucleus via the Srp1/Kap95 Importin heterodimer, are then interact with NF-Y through different modules: Ssr4/Sol1/Snf59/Fif1 mediates SWI/SNF interaction, while Ssr4/Rsc7/Fif3 facilitates RSC interaction. Subsequently, SWI/SNF and RSC utilize ATPase-driven chromatin remodeling to generate a loosened or compact chromatin conformation, thereby activating or repressing the expression of certain NF-Y-dependent genes.

Many studies report TF NF-Y as a positive regulator of cellulolytic gene expression in filamentous fungi under lignocellulosic conditions [57–60], consistent with our observation of cellulolytic gene downregulation in ΔYB and ΔYC, and SWI/SNF-specific mutant Δfif1. However, we note that while cellulolytic gene expression is significantly reduced in ΔYB and ΔYC, it is upregulated in the RSC-specific mutant Δfif3. This inverse relationship indicates that cellulolytic gene upregulation in Δfif3 results specifically from PoFif3 absence rather than defective NF-Y-RSC coordination.

While direct physical interactions between TFs and CRC holo-complexes remain undocumented in filamentous fungi, studies in humans and plants demonstrate that SWI/SNF-type complexes functionally interplay with multiple TFs [61], including some master TFs [62, 63]. Indeed, another TF was identified in our study. Specifically, a bZIP TF, PDE_09 548 (UniProt S7ZVW5), designated HapX in fungi [64], was consistently detected in triplicate TAP-MS analyses using either the core ATPase subunit PoSnf21 or the RSC-specific subunit PoFif3 as bait (Supplementary Spreadsheet S4 Sheet 5, rank 53; Sheet 8, rank 249). This finding suggests an association of HapX with the RSC complex.

Interestingly, domain analysis of HapX revealed that it contains an 18-amino-acid Hap4_Hap_bind domain (the minimal binding motif of Hap4 for interaction with Hap2/3/5), similar to that found in *S. cerevisiae* Hap4 (Supplementary Fig. S19). In *S. cerevisiae*, Hap4 functions as a separate activation subunit alongside the trimeric Hap2/3/5 complex and interacts with several SWI/SNF subunits, including one subunit (Swp61/Arp7) shared by SWI/SNF and RSC and three SWI/SNF-specific subunits (Snf5, Swi1, Swi2/Snf2), suggesting an association with the SWI/SNF complex [65, 66]. However, this architecture is not evolutionarily conserved. Unlike *S. cerevisiae*, the NF-Y/CBC complexes in *S. pombe*, filamentous fungi, plants, and animals consist of only three subunits and lack a Hap4 ortholog. In fact, Hap4 appears to be specific to *S. cerevisiae*; even in the closely related yeast *Kluyveromyces lactis* (also a member of Saccharomycotina), the analogous protein (643 amino acids) exhibits only two small regions (11 and 16 amino acids) with high homology to *S. cerevisiae* Hap4, while overall sequence similarity remains weak [67]. Thus, in organisms lacking Hap4, alternative mechanisms must operate.

Despite the presence of the Hap4_Hap_bind domain in HapX, this protein was not a functional Hap4 homolog [68]. Corroborating this, our TAP assays with NF-YA, NF-YB, and NF-YC as baits did not detect HapX, whereas the three NF-Y subunits were consistently identified as top three hits. These results support that HapX is not a component of the NF-Y complex and that the interaction between NF-Y and CRC holo-complexes occurs independently of HapX. Interestingly, in *A. nidulans*, HapX is recognized as an AP-1-like TF and has been shown to dimerize and cooperate with the NF-Y complex in DNA recognition [69, 70]. Cooperation between SWI/SNF and AP-1 complexes has also been documented in human cells, revealing a dependence on AP-1-mediated chromatin localization [71]. Taken together, our findings point to a potential model in which, in species lacking Hap4, the NF-Y complex interacts with CRCs independently; although NF-Y and HapX/AP-1 operate separately, both are capable of engaging with CRC complexes—an interplay whose underlying mechanisms await future investigation.

Consequently, we propose that under lignocellulosic conditions, the NF-Y-SWI/SNF complex activates the expression of certain CWDE genes, while other TFs (or HapX) form repressive complexes with RSC to repress CWDE gene expression. Such repression is comparatively weaker than the NF-Y-SWI/SNF-mediated activation. This antagonism establishes a counterbalancing mechanism governing CWDE gene transcription (Fig. 10, left). Interestingly, although this model implies that the regulatory role of RSC in CWDE gene expression may not be directly linked to NF-Y, the enrichment of the RSC-specific PoFif3 at the promoter regions of two CWDE genes was also dependent on NF-Y. One possible explanation for this contradiction lies in the frequent co-localization of CCAAT boxes, the binding sites of the NF-Y complex, with the motifs of CWDE-related TFs in filamentous fungi. For instance, in the promoter region of the *T. reesei* hemicellulase gene *xyn1*, a CCAAT box is located in close proximity to the motifs recognized by TF Cre1 and Xyr1 [72], both of which are key regulators of CWDE gene expression in filamentous fungi [73]. Notably, Cre1 has been shown to be associated with nucleosome positioning within the cellulase gene *cbh1* (*cel7A*) [74]. Xyr1, whose promoter contains a CCAAT box and is positively regulated by NF-Y [75], has also been reported to activate CWDE gene expression through direct recruitment of the SWI/SNF [76]. In the *P. oxalicum cel7A-1* promoter, a binding motif for TF Ace1, a repressor of cellulase genes, is present in the R2 region (Fig. 9A, position -193, not marked [77]. Therefore, as a global TF, deletion of NF-Y subunits may trigger cascading effects that influence the expression or activity of other TFs, which in turn could affect the recruitment of the RSC complex to target gene promoters.

Many studies have established TF NF-Y as a positive regulator of fungal growth and development [78, 79], consistent with our observed growth and developmental impairment in HK-YC, Δfif1, and Δfif3 under glucose conditions. Nevertheless, potential contributions from alternative TFs persist. Notably, the PoFif3-interacting protein HapX, beyond its primary function in iron homeostasis, demonstrates positive correlation with fungal growth and developmental processes [80, 81]. Given that Δfif3 exhibited more severe defects than Δfif1, we propose that while both NF-Y-SWI/SNF and NF-Y-RSC complexes promote fungal growth and development, the latter may exert a stronger influence (Fig. 10, right).

Although we propose that NF–Y and SWI/SNF/RSC cooperatively regulate CWDE gene transcription, fungal growth, and asexual development, this model has several key limitations. First, while a direct interaction between NF-Y and the SWI/SNF and RSC complexes is well established, and Pofif1 and Pofif3 have NF-Y-dependent enrichment at the promoters of several genes, this does not imply that NF-Y regulates all CCAAT-box-containing genes through the recruitment of these remodelers. The analysis of the relationship between the DEGs in Δfif1 and Δfif3 and the NF-YC binding motif (CCAAT box) showed that among the DEGs identified under cellulose conditions and glucose conditions, the proportions containing a CCAAT box within the −200 bp promoter region were 34.1% in Δfif1 (cellulose), 36.0% in Δfif1 (glucose), 34.5% in Δfif3 (cellulose), and 35.1% in Δfif3 (glucose). Compared to the genomic background, in which 3407 of the 10 021 predicted genes (34.0%) in the WT strain harbor a CCAAT box, these percentages are only slightly elevated and do not represent a significant enrichment (Supplementary Spreadsheet S12). Namely, the recruitment of CRCs by TFs is often gene-specific rather than following a global, invariant pattern [82]. For example, GATA3, a pioneer TF, colocalizes with the SWI/SNF remodeler BRG1 only at “productive sites” where open chromatin is established. In contrast, at “unproductive sites,” GATA3 binding occurs without concomitant BRG1 recruitment [83]. More broadly, genome-wide approaches such as ChIP-seq or ATAC-seq are required to distinguish between permissive NF-Y binding events and those that result in functional SWI/SNF or RSC recruitment. Second, gene expression changes in Δfif1 and Δfif3 mutants cannot be entirely ascribed to impaired NF-Y-dependent engagement with SWI/SNF and RSC complexes, as CRC deficiencies typically exert genome-wide regulatory effects [84]. SWI/SNF and RSC complexes engage not only with TFs but also recruit histone acetyltransferases and other chromatin modifiers [62, 85], creating multifaceted regulatory networks. Third, the current model does not incorporate the role of NF-Y in mediating CCR alleviation to enhance CWDE gene expression under glucose (repressing) conditions [79]. Previous studies have shown that increasing the copy number of CCAAT boxes in the *T. reesei* cellulase gene *cbh1* promoter abolishes glucose repression [58], and synthetic promoters incorporating Hap2/3/5 binding sites enable elevated cellulase gene expression even under repressive conditions [59, 60]. However, whether this CCR-alleviating function operates through SWI/SNF or RSC remains unknown. Our transcriptomic data under glucose conditions show that cellulase genes remain strongly repressed in both Δfif1 and Δfif3 mutants, suggesting that NF-Y’s ability to relieve CCR may involve mechanisms independent of these remodelers.

Studies on SWI/SNF-specific PoFif1 and RSC-specific PoFif3 demonstrate that these two subunits exhibit both shared and distinct functional roles. Transcriptomic analysis revealed that Δfif3 regulates substantially more genes than Δfif1 under both lignocellulosic and glucose conditions. Their target gene sets show limited overlap; under cellulose, only 25.6% of Δfif1-upregulated and 12.1% of Δfif3-upregulated genes overlapped. Their regulation of most CWDE genes was even opposing. In addition, upregulated DEGs in Δfif3, but not in Δfif1, were enriched in nuclear chromosome-related terms. This pattern is consistent with the well-established functional divergence between SWI/SNF and RSC: the two complexes exhibit non-overlapping and opposing regulatory roles [86, 87], with RSC displaying a broader regulatory scope, governing wider chromatin dynamics, and being more essential for viability [88–90]. Nevertheless, as Fif1, Fif3, and the newly identified Fif2 and Fif4 are all newly identified subunits, their precise contributions to complex integrity, remodeling activity, or potential non-complex functions remain unclear. Further biochemical and genomic studies will be essential to dissect the specific MFs of these four subunits.”

The interaction between NF-Y and CRC subunits appears to be mediated by conserved structural features. It is unsurprising that the NF-Y complex simultaneously interacts with multiple subunits within a target complex [91]. However, the ability of a single CRC subunit to interact with NF-YA, NF-YB, and NF-YC may seem counterintuitive, despite autoactivation test in the Y2H strains confirming the absence of false-positive signals. This phenomenon can be explained by the intrinsic glutamine-rich (Q-rich) domains present in all NF-Y subunits, which serve as established platforms for cofactor recruitment [5, 92, 93]. A parallel interaction occurs in *Drosophila*, where general TF TFIID recognizes Q-rich domains of both CBF-B and CBF-C [94]. However, other factors cannot be excluded. For instance, endogenous yeast NF–Y or CRCs complexes could act as bridges; a positive signal may thus reflect an interaction mediated by an assembled endogenous complex rather than direct subunit–subunit contact. Furthermore, certain domains or regions within the tested subunits may exhibit non–specific “protein stickiness” in the Y2H environment [95], potentially yielding positive signals even in the absence of physiologically relevant interactions. The precise molecular determinants in this system warrant further investigation.

When validating the interaction between NF-Y and CRCs, reciprocal experiments using SWI/SNF or RSC subunits as bait failed to detect NF-Y subunits, a phenomenon consistent with prior reports of TF-cofactor detection asymmetry [77, 96]. The low abundance of TFs offers one potential explanation for this observation. For instance, *S. cerevisiae* under identical culture conditions exhibits significantly higher levels of the RSC core subunit Sth1 (10 149 molecules/cell) than the NF-YA subunit (1698 molecules/cell) [97]. In addition, cofactors, particularly co-repressors, have been found to be over tenfold more abundant than TFs in mammalian cells [98]. An alternative possibility is that using a specific cofactor subunit as bait may trigger competitive binding between the target TF and other proteins (including other TFs). This could be attributable to the multi-domain architecture and diverse protein interaction interfaces inherent in multi-subunit cofactors, a model termed “cofactor squelching” [99].

In summary, our results speculatively raise the possibility that NF-Y’s “pseudo-chromatin remodeler” activity could, in part, be explained by its ability to recruit two *bona fide* CRCs: SWI/SNF and RSC. Furthermore, dissection of these two CRCs’ composition in two filamentous fungi revealed four fungus-specific subunits (Fif1–Fif4), providing the first mechanistic framework for lineage-specific chromatin regulation in this ecologically and biotechnologically vital group. Although our experimental system employs filamentous fungi, the evolutionary conservation of NF-Y, SWI/SNF, and RSC complexes from fungi to mammals raises the possibility that NF-Y’s interaction with SWI/SNF and RSC as cofactors represents a universal regulatory paradigm across eukaryotes.

Nevertheless, questions still remain: Does NF-Y exclusively interact with one CRC (SWI/SNF or RSC), or do SWI/SNF and RSC compete for binding to NF-Y at specific loci? How do HapX and/or other TFs integrate into this network? The extent of interspecies variation also remains an open question. While our work in fungi demonstrates that NF-Y interacts with SWI/SNF and RSC and coordinately regulates gene expression, a recent study in mammalian embryonic stem cells reports that NF-Y-dependent promoters remain largely unaffected upon depletion of BRG1, the ATPase of the BAF complex [100]. Thus, the role of NF-Y does not appear fixed: it may act as a remodeler-independent “maintenance” factor in one context, and as a remodeler-dependent recruiter of CRCs in another. This discrepancy may be explained by the fact that the need for chromatin remodelers differs at different promoters or even at the same promoter under different circumstances [82]. Future studies mapping SWI/SNF and RSC occupancy at NF-Y-bound promoters under dynamic conditions will clarify these mechanisms.

## Supplementary Material

gkag543_Supplemental_Files

## Data Availability

Raw MS/MS data for TAP-MS are deposited in iProX (https://www.iprox.cn) under accession codes PXD063169, PXD060703, PXD060705, PXD060617, PXD063167, PXD060670, and PXD072990 (Note: Hap2 = NF-YA, Hap3 = NF-YB, Hap5 = NF-YC). The raw data of transcriptome sequencing have been deposited in NCBI’s Gene Expression Omnibus database under the accession numbers GSE289700 and GSE261111.
