# Supplementary material for: Transcription factor NF-Y complex interacts with chromatin remodeling complexes SWI/SNF and RSC to coordinately regulate gene expression
Source: Nucleic Acids Res. 2026 May 30;54(10):gkag543. doi: 10.1093/nar/gkag543 (PMC13221647; doi:10.1093/nar/gkag543)
Supplement: gkag543_Supplemental_Files [file gkag543_supplemental_files.zip › Supplementary Materials for NARNAR-03098-V-2025.R2- including dataset captions.pdf]

Supplementary Materials for

Transcription factor NF-Y Complex Interacts with Chromatin Remodeling Complexes SWI/SNF and RSC to Coordinately Regulate Gene Expression

Kexuan Ma *et al.*

*Corresponding author. Email: qinyuqi@sdu.edu.cn

This PDF file includes:

Supplementary Table S1
Supplementary Figs. S1 to S19
Captions of Supplementary dataset S1 to S12

Other Supplementary Materials for this manuscript include the following:

Separate DATASET files: Spreadsheet S1 to S12

Table S1 Description of 28 secondary metabolic gene clusters in *P. oxalicum*

Cluster	Gene ID (locus_tag)	SMURF "backbone gene" prediction [a]	Oxidase/ reductase	Corresponding to	
				Fig. 8A	Fig. 8B
Cluster_1	PDE_00787 - PDE_00814	NRPS (PDE_00789)<br>NRPS (PDE_00793)<br>DMAT(PDE_00797)<br>DMAT(PDE_00807)<br>NRPS (PDE_00810)	Yes	II	II
Cluster_2	PDE_01064 - PDE_01077	DMAT (PDE_01066)<br>NRPS (PDE_01071)<br>NRPS (PDE_01077)	Yes	III	
Cluster_3	PDE_01166 - PDE_01177	PKS-Like (PDE_01173)			
Cluster_4	PDE_01185 - PDE_01198	NRPS-Like (PDE_01185)		IV	
Cluster_5	PDE_01212 - PDE_01220	NRPS (PDE_01212)		V	IV
Cluster_6	PDE_01230 - PDE_01242	PKS (PDE_01235)	Yes		V
Cluster_7	PDE_01418 - PDE_01233	NRPS (PDE_01432)	Yes		
Cluster_8	PDE_02130 - PDE_02141	NRPS (PDE_02131)<br>PKS (PDE_02141)	Yes		
Cluster_9	PDE_02203 - PDE_02204	NRPS-Like (PDE_02203)	Yes		
Cluster_10	PDE_02600 - PDE_02610	NRPS (PDE_02610)	Yes		
Cluster_11	PDE_03298 - PDE_03304	NRPS (PDE_03304)	Yes		
Cluster_12	PDE_03415- PDE_03416	NRPS (PDE_03416)			
Cluster_13	PDE_03453- PDE_03455	PKS (PDE_03455)			
Cluster_14	PDE_03920- PDE_03926	PKS (PDE_03926)			
Cluster_15	PDE_04008- PDE_04024	HYBRID (PDE_04017)<br>PKS (PDE_04018)	Yes	VI	
Cluster_16	PDE_04252- PDE_04267	HYBRID (PDE_04252)			
Cluster_17	PDE_04540 - PDE_04545	NRPS (PDE_04253)			
Cluster_18	PDE_05556 - PDE_05569	NRPS (PDE_05564)			
Cluster_19	PDE_06202 - PDE_06213	NRPS-Like (PDE_02203)			
Cluster_20	PDE_06623 - PDE_06230	NRPS-Like (PDE_06630)	Yes		
Cluster_21	PDE_07001 - PDE_07006	NRPS (PDE_07005)	Yes		
Cluster_22	PDE_07162 - PDE_07172	NRPS (PDE_07173)			
Cluster_23	PDE_07373 - PDE_07378	NRPS-Like (PDE_07373)			
Cluster_24	PDE_08154 - PDE_08155	NRPS-Like (PDE_08155)		VII	
Cluster_25	PDE_09188 - PDE_09202	HYBRID (PDE_09198)	Yes		
Cluster_26	PDE_09227 - PDE_09243	PKS (PDE_09237)	Yes	VIII	XI
Cluster_27	PDE_09491 - PDE_09496	PKS (PDE_09491)	Yes		
Cluster_28	PDE_09499 - PDE_10009	PKS (PDE_10006)	Yes		

[a]: DMAT, Demethylallyl tryptophan synthase; NRPS, Nonribosomal peptide synthetases; PKS, Polyketide synthases; HYBRID, PKS-NRPS hybrid.

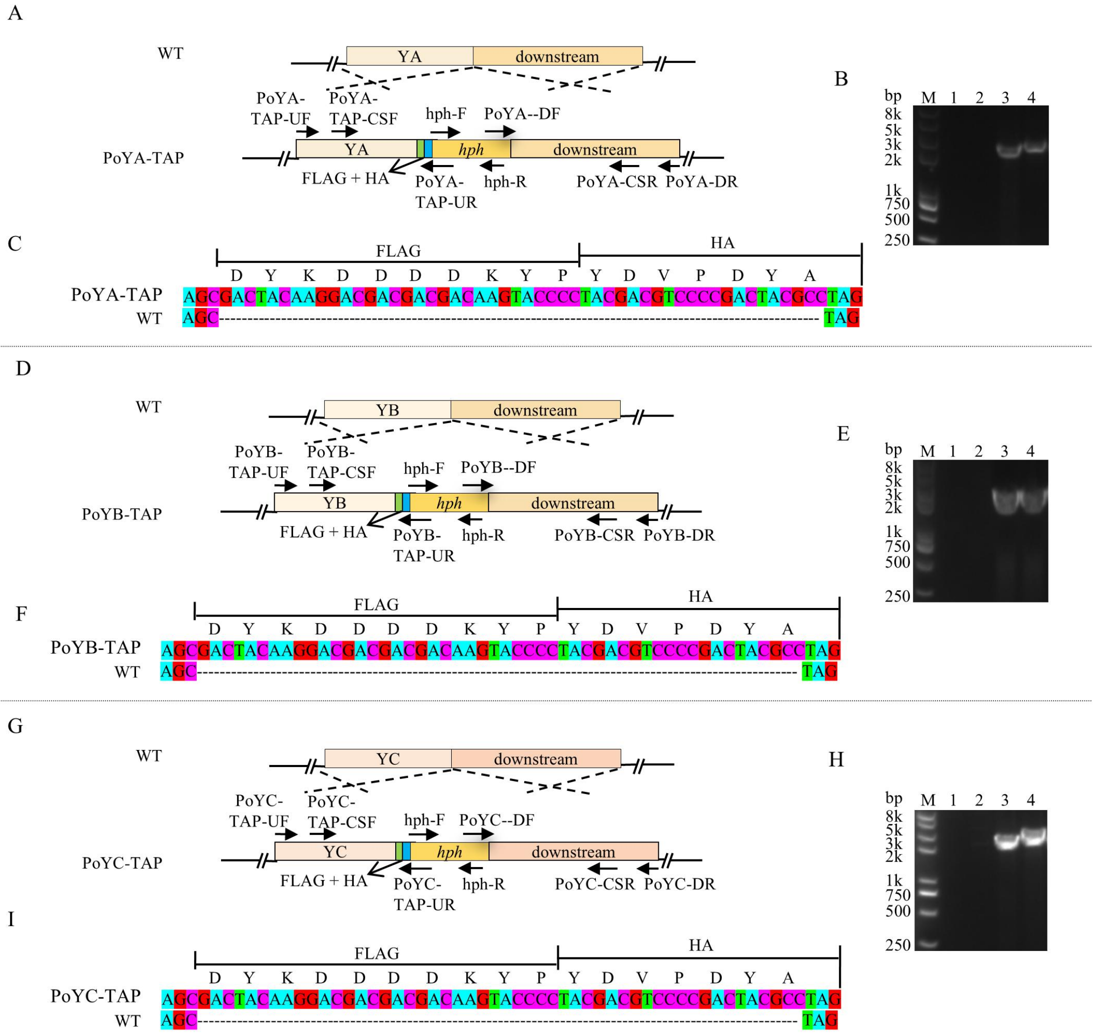

Fig. S1. Construction strategies and verification of strains PoYA-/B-/C-TAP. (A) Construction strategy of PoYA-TAP strain. (B) Results of diagnostic PCR of PoYA-TAP strain. Lane 1 and lane 2 represent the control *P. oxalicum* WT; lane 3 (2743 bp) and lane 4 (2867 bp) represent PoYA-TAP (amplified using primers PoYA-TAP-UF/hph-YZR and hph-YZF/PoYA-TAP-DR, respectively). (C) Sequencing results of the protein PoYA fused with the TAP (FALG-HA) tag.(D) Construction strategy of PoYB-TAP strain. (E) Results of diagnostic PCR of PoYB-TAP strain. Lane 1 and lane 2 represent the control *P. oxalicum* WT; lane 3 (2642 bp) and lane 4 (2723 bp) represent PoYB-TAP (amplified using primers PoYB-TAP-UF/hph-YZR and hph-YZF/PoYB-TAP-DR, respectively). (F) Sequencing results of the protein PoYB fused with the TAP (FALG-HA) tag. (G) Construction strategy of PoYC-TAP strain. (H) Results of diagnostic PCR of PoYC-TAP strain. Lane 1 and lane 2 represent the control *P. oxalicum* WT; lane 3 (2481 bp) and lane 4 (2949 bp) represent PoYC-TAP (amplified using primers PoYC-TAP-UF/hph-YZR and hph-YZF/PoYC-TAP-DR, respectively). (I) Sequencing results of the protein PoYC fused with the TAP (FALG-HA) tag.

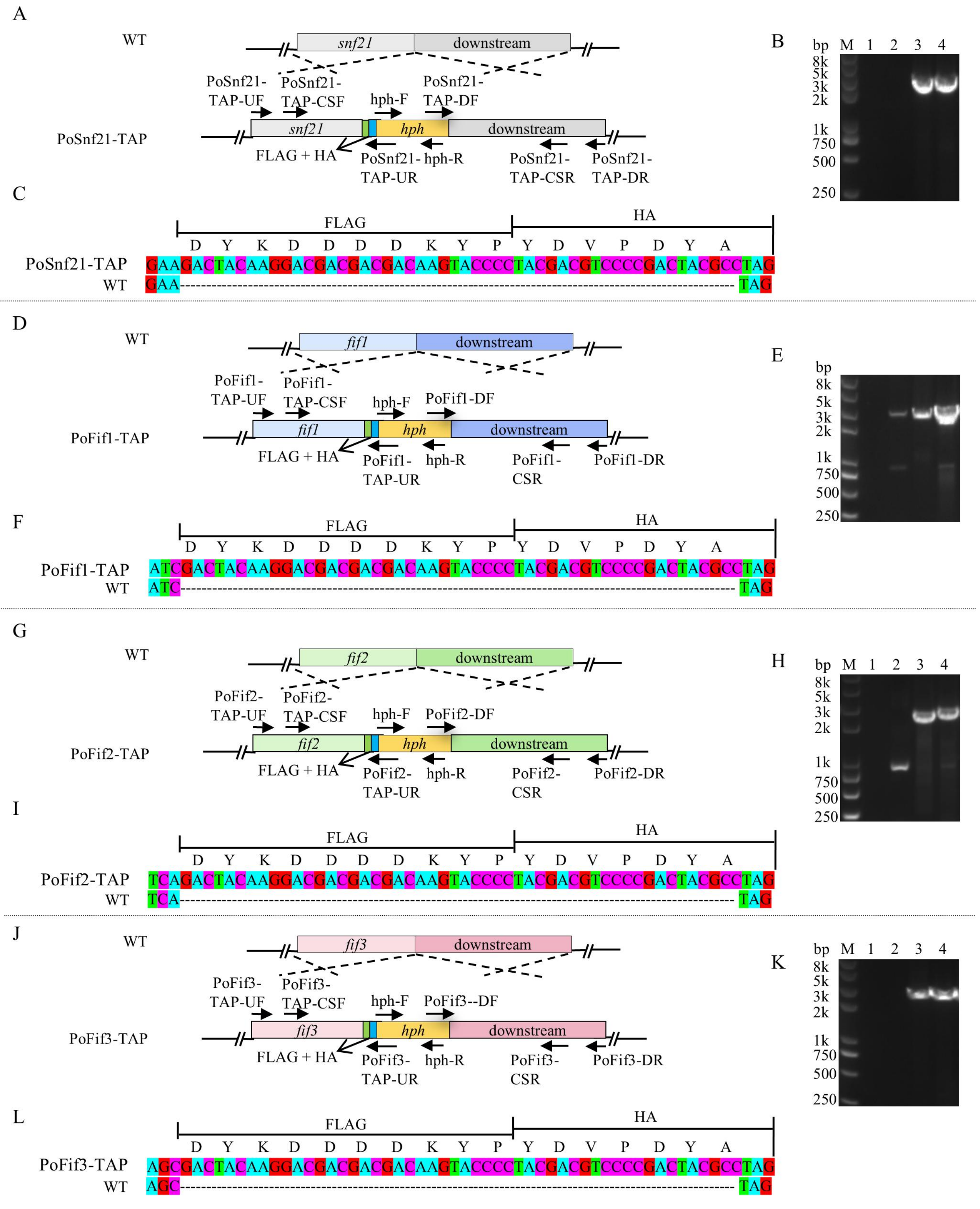

Fig. S2. Construction strategies and verification of strains PoSnf21-/Fif1-/Fif2-/Fif3-TAP. (A) Construction strategy of PoSnf21-TAP strain. (B) Results of diagnostic PCR of PoSnf21-TAP strain. Lane 1 and lane 2 represent the control *P. oxalicum* WT; lane 3 (2595 bp) and lane 4 (2677

bp) represent PoSnf21-TAP (amplified using primers PoSnf21-TAP-UF/hph-YZR and hph-YZF/PoSnf21-TAP-DR, respectively). (C) Sequencing results of the protein PoSnf21 fused with the TAP (FALG-HA) tag. (D) Construction strategy of PoFif1-TAP strain. (E) Results of diagnostic PCR of PoFif1-TAP strain. Lane 1 and lane 2 represent the control *P. oxalicum* WT; lane 3 (2418 bp) and lane 4 (2806 bp) represent PoFif1-TAP (amplified using primers PoFif1-TAP-UF/hph-YZR and hph-YZF/PoFif1-TAP-DR, respectively). (F) Sequencing results of the protein PoFif1 fused with the TAP (FALG-HA) tag. (G) Construction strategy of PoFif2-TAP strain. (H) Results of diagnostic PCR of PoFif2-TAP strain. Lane 1 and lane 2 represent the control *P. oxalicum* WT; lane 3 (2531 bp) and lane 4 (2713 bp) represent PoFif2-TAP (amplified using primers PoFif2-TAP-UF/hph-YZR and hph-YZF/PoFif2-TAP-DR, respectively). (I) Sequencing results of the protein PoFif2 fused with the TAP (FALG-HA) tag. (J) Construction strategy of PoFif3-TAP strain. (K) Results of diagnostic PCR of PoFif3-TAP strain. Lane 1 and lane 2 represent the control *P. oxalicum* WT; lane 3 (2623 bp) and lane 4 (2730 bp) represent PoFif3-TAP (amplified using primers PoFif3-TAP-UF/hph-YZR and hph-YZF/PoFif3-TAP-DR, respectively). (L) Sequencing results of the protein PoFif3 fused with the TAP (FALG-HA) tag.

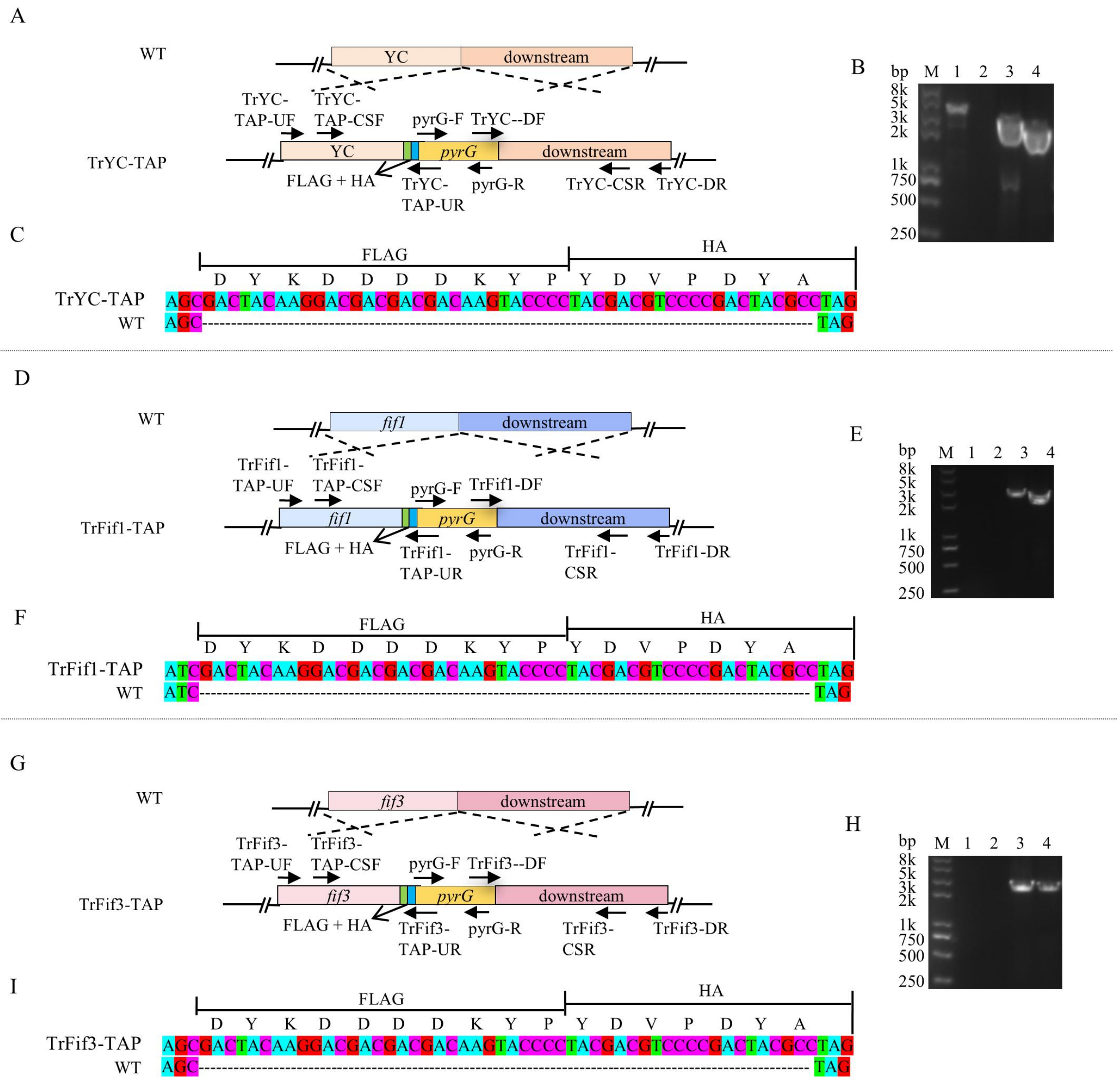

Fig. S3. Construction strategies and verification of strains TrYC-/Fif1-/Fif3-TAP. (A) Construction strategy of TrYC-TAP strain. (B) Results of diagnostic PCR of TrYC-TAP strain. Lane 1 and lane 2 represent the control *T. reesei* WT; lane 3 (2776 bp) and lane 4 (2341 bp) represent TrYC-TAP (amplified using primers TrYC-TAP-UF/pyrG-YZR and pyrG-YZF/TrYC-TAP-DR, respectively). (C) Sequencing results of the protein TrYC fused with the TAP (FALG-HA) tag. (D) Construction strategy of TrFif1-TAP strain. (E) Results of diagnostic PCR of TrFif1-TAP strain. Lane 1 and lane 2 represent the control *T. reesei* WT; lane 3 (2642 bp) and lane 4 (2541 bp) represent TrFif1-TAP (amplified using primersTrFif1-TAP-UF/pyrG-YZR and pyrG-YZF/TrFif1-TAP-DR, respectively). (F) Sequencing results of the protein TrFif2 fused with the TAP (FALG-HA) tag. (G) Construction strategy of TrFif2-TAP strain. (H) Results of diagnostic PCR of TrFif2-TAP strain. Lane 1 and lane 2 represent the control *T. reesei* WT; lane 3 (2561 bp) and lane 4 (2587 bp) represent TrFif2-TAP (amplified using primers TrFif2-TAP-UF/pyrG-YZR and pyrG-YZF/TrFif2-TAP-DR, respectively). (I) Sequencing results of the protein TrFif2 fused with the TAP (FALG-HA) tag.

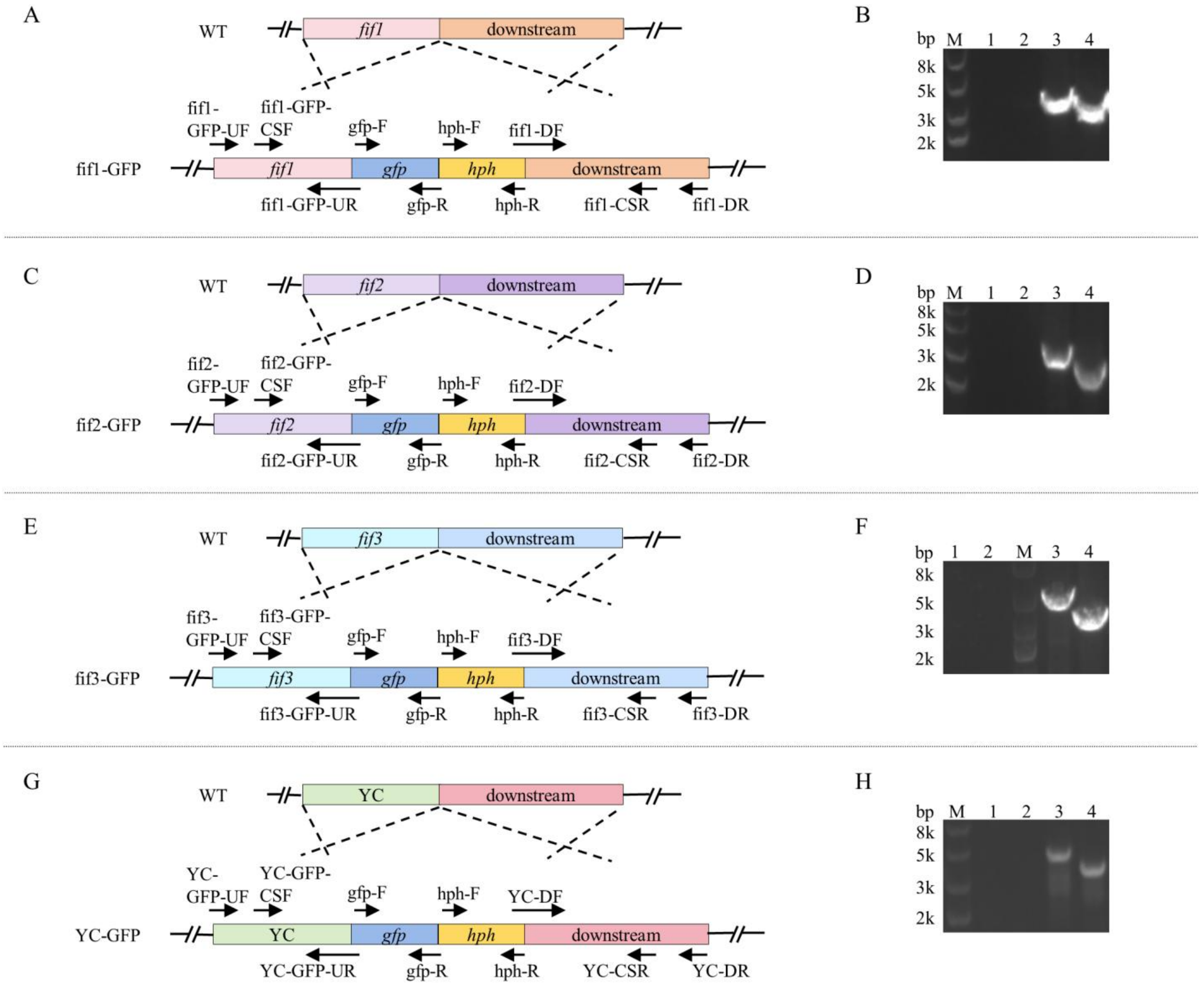

Fig. S4. Construction strategies and verification of strains Fif1/2/3-GFP and YC-GFP. (A) Construction strategy of Fif1-GFP strain. (B) Results of diagnostic PCR of Fif1-GFP strain. Lane 1 and lane 2 represent the control *P. oxalicum* WT; lane 3 (3018 bp) and lane 4 (2806 bp) represent Fif1-GFP (amplified using primers Fif1-GFP-UF/hph-YZR and hph-YZF/Fif1-DR, respectively).(C) Construction strategy of Fif2-GFP strain. (D) Results of diagnostic PCR of Fif2-GFP strain. Lane 1 and lane 2 represent the control *P. oxalicum* WT; lane 3 (3209 bp) and lane 4 (2713 bp) represent Fif2-GFP (amplified using primers Fif2-GFP-UF/hph-YZR and hph-YZF/Fif2-DR, respectively). (E) Construction strategy of Fif3-GFP strain. (F) Results of diagnostic PCR of Fif3-GFP strain. Lane 1 and lane 2 represent the control *P. oxalicum* WT; lane 3 (3355 bp) and lane 4 (2730 bp) represent Fif3-GFP (amplified using primers Fif3-GFP-UF/hph-YZR and hph-YZF/Fif3-DR, respectively). (G) Construction strategy of YC-GFP strain. (H) Results of diagnostic PCR of YC-GFP strain. Lane 1 and lane 2 represent the control *P. oxalicum* WT; lane 3 (3320 bp) and lane 4 (2723 bp) represent YC-GFP (amplified using primers YC-GFP-UF/hph-YZR and hph-YZF/YC-DR, respectively).

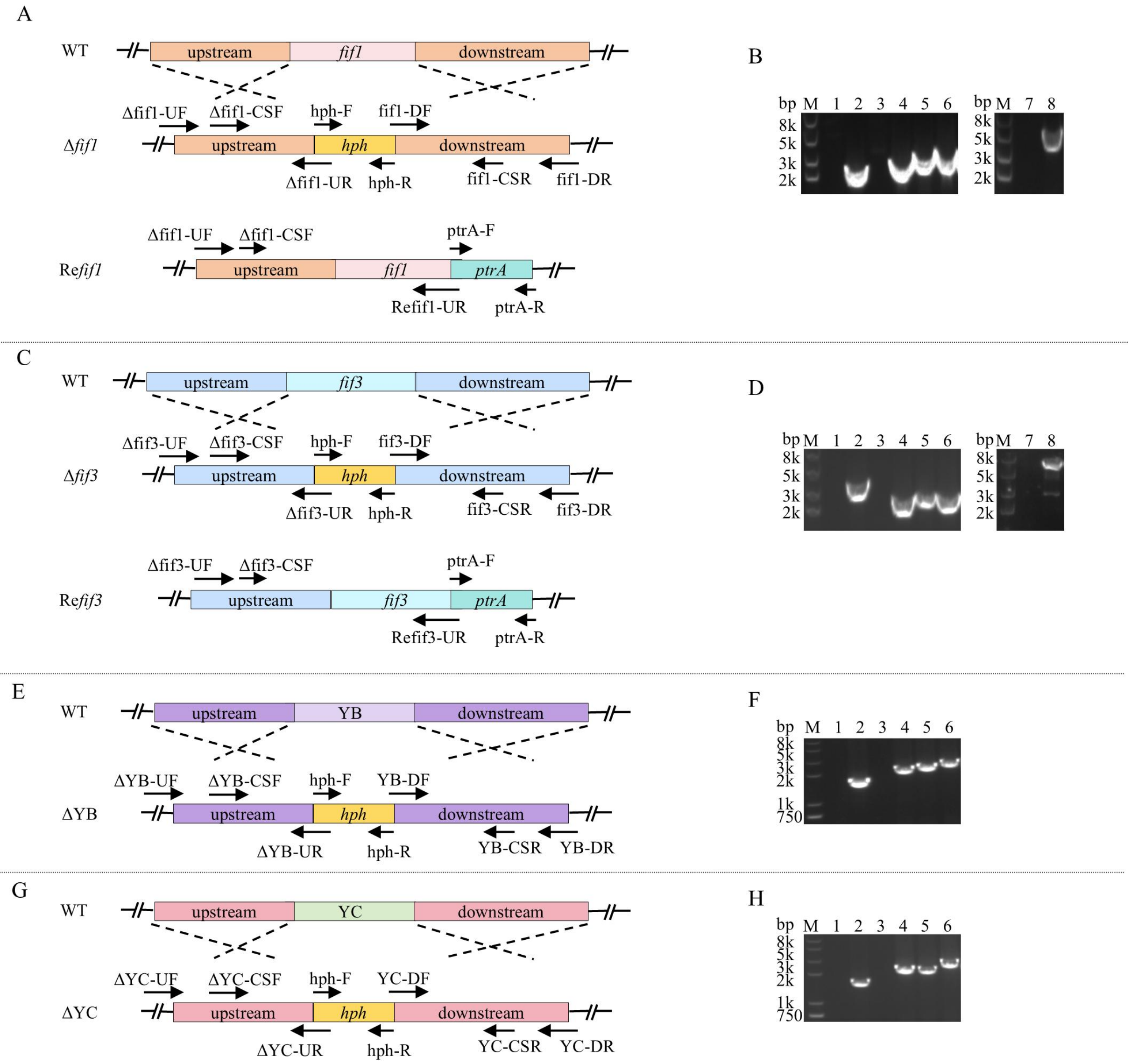

Fig. S5. Construction strategies and verification of strains Δfif1/3, Refif1/3 and ΔYB/C. (A) Construction strategy of Δfif1 strain. (B) Results of diagnostic PCR of Δfif1 strain. Lanes 1, 2, and 3 represent the negative control WT; lane 4 (2409 bp). Lane 5 (2576 bp) and Lane 6 (2805 bp) represent Δfif1 (amplified using primers Δfif1-UF/hph-YZR, Δfif1-YZF/Δfif1-YZR and hph-YZF/fif1-DR, respectively). Lane 7 represents the control P. oxalicum WT, and lane 8 (5302 bp) represent Refif1 (amplified using primers Δfif1-CSF/ptrA-YZR). (C) Construction strategy of Δfif3 strain. (D) Results of diagnostic PCR of Δfif3 strain. Lanes 1, 2, and 3 represent the negative control WT; lane 4 (2143 bp). Lane 5 (2135 bp) and Lane 6 (2016 bp) represent Δfif3 (amplified using primers Δfif3-UF/hph-YZR, Δfif3-YZF/Δfif3-YZR and hph-YZF/fif3-DR, respectively). Lane 7 represents the control *P. oxalicum* WT, and lane 8 (5834 bp) represent Refif3 (amplified using primers Δfif3-CSF/ptrA-YZR). (E) Construction strategy of ΔYB strain. (F) Results of diagnostic PCR of ΔYB strain. Lanes 1, 2, and 3 represent the negative control WT; lane 4 (2336 bp), Lane 5 (2453 bp) and Lane 6 (2723 bp) represent ΔYB (amplified using primers ΔYB-UF/hph-YZR, ΔYB-YZF/ΔYB-YZR and hph-YZF/YB-DR, respectively). (G) Construction strategy of ΔYC strain. (H)

Results of diagnostic PCR of ΔYC strain. Lanes 1, 2, and 3 represent the control WT; lane 4 (2422 bp), Lane 5 (2410 bp) and Lane 6 (2819 bp) represent ΔYC (amplified using primers ΔYC-UF/hph-YZR, ΔYC-YZF/ΔYC-YZR and hph-YZF/YC-DR, respectively).

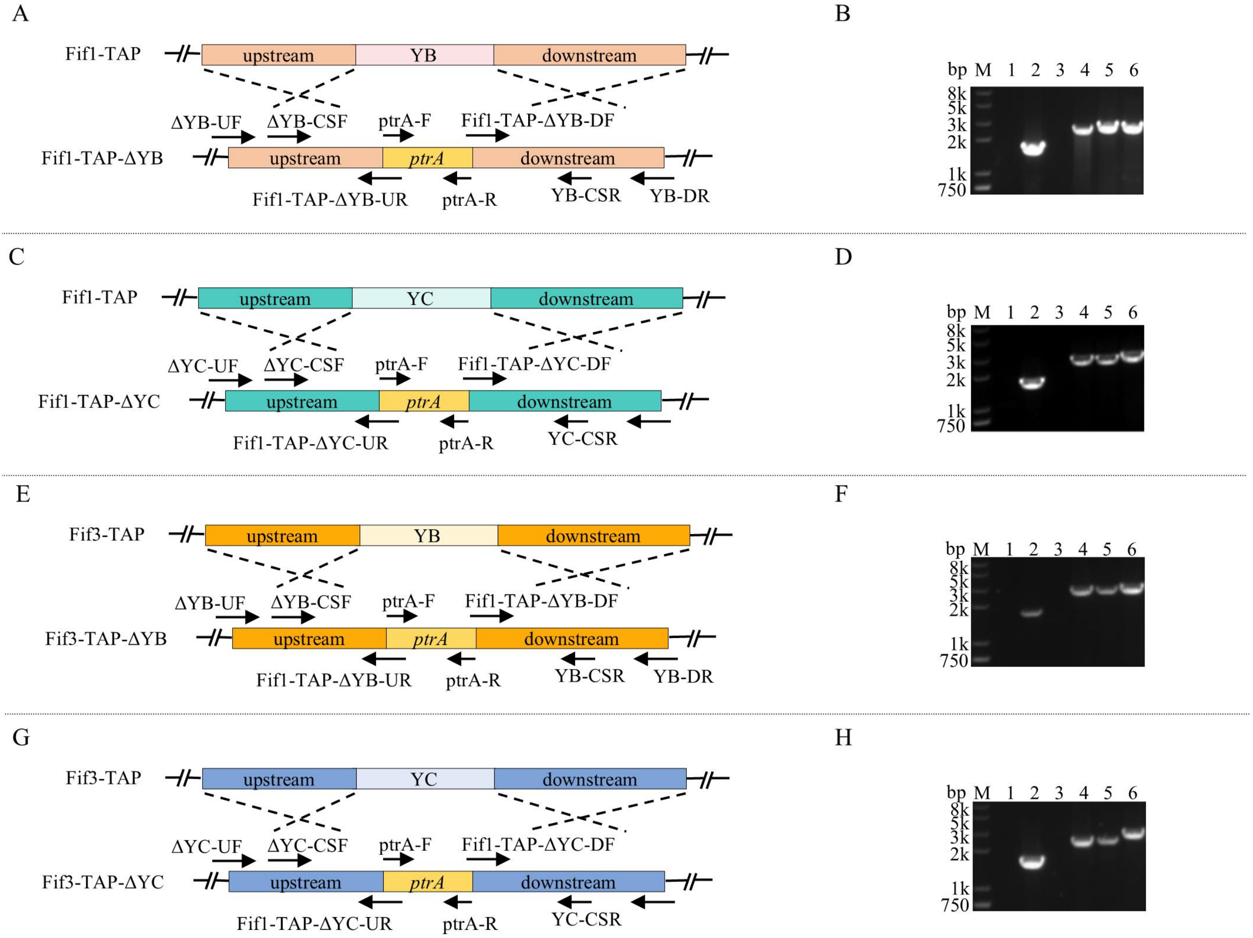

Fig. S6. Construction strategies and verification of strains Fif1-TAP-ΔYB/C and Fif3-TAP-ΔYB/C. (A) Construction strategy of Fif1-TAP-ΔYB strain. (B) Results of diagnostic PCR of Fif1-TAP-ΔYB strain. Lanes 1, 2, and 3 represent the control WT; lane 4 (2411 bp). Lane 5 (2561 bp) and Lane 6 (2407 bp) represent Fif1-TAP-ΔYB (amplified using primers ΔYB-UF/ptrA-YZR, ΔYB-YZF/ΔYB-YZR and ptrA-YZF/YB-DR, respectively). (C) Construction strategy of Fif1-TAP-ΔYC strain. (D) Results of diagnostic PCR of Fif1-TAP-ΔYC strain. Lanes 1, 2, and 3 represent the control WT; lane 4 (2497 bp). Lane 5 (2518 bp) and Lane 6 (2703 bp) represent Fif1-TAP-ΔYC (amplified using primers ΔYC-UF/ptrA-YZR, ΔYC-YZF/ΔYC-YZR and ptrA-YZF/YC-DR, respectively). (E) Construction strategy of Fif3-TAP-ΔYB strain. (F) Results of diagnostic PCR of Fif3-TAP-ΔYB strain. Lanes 1, 2, and 3 represent the negative control WT; lane 4 (2411 bp). Lane 5 (2561 bp) and Lane 6 (2407 bp) represent Fif3-TAP-ΔYB (amplified using primers ΔYB-UF/ptrA-YZR, ΔYB-YZF/ΔYB-YZR and ptrA-YZF/YB-DR, respectively). (G) Construction strategy of Fif3-TAP-ΔYC strain. (H) Results of diagnostic PCR of Fif3-TAP-ΔYC strain. Lanes 1, 2, and 3 represent the control WT; lane 4 (2497 bp). Lane 5 (2518 bp) and Lane 6 (2703 bp) represent Fif3-TAP-ΔYC (amplified using primers ΔYC-UF/ptrA-YZR, ΔYC-YZF/ΔYC-YZR and ptrA-YZF/YC-DR, respectively).

A

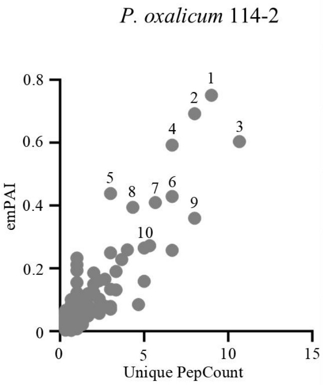

Rank	Name	Description
1	PDE_01092	Actin
2	PDE_09952	Glyceraldehyde-3-phosphate dehydrogenase
3	PDE_04963	Serine hydroxymethyltransferase
4	PDE_04897	Ubiquitin-like domain-containing protein
5	PDE_00491	40S ribosomal protein S22
6	PDE_03961	Ubiquitin-like domain-containing protein
8	PDE_02754	40S ribosomal protein S3
9	PDE_00406	Small ribosomal subunit protein
10	PDE_02596	Elongation factor 1-alpha

B

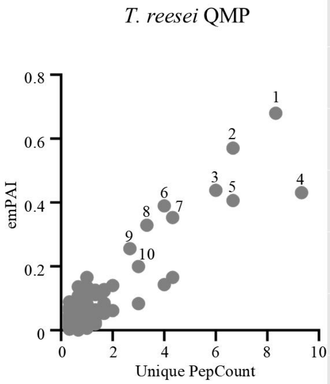

Rank	Name	Description
1	XP_006961104.1	Actin
2	XP_006968883.1	Ubiquitin/small ribosomal subunit protein
3	XP_006961373.1	40S ribosomal protein S3
4	XP_006964056.1	Elongation factor 1-alpha
5	XP_006966790.1	Polyubiquitin
6	XP_006965373.1	-
8	XP_006965357.1	Ribosomal protein S5
9	XP_006962784.1	GTP-binding nuclear protein
10	XP_006965398.1	Ribosomal protein S14

Fig. S7. TAP-MS results for the control strains *P. oxalicum* 114-2 (A) and *T. reesei* QMP (B). Detailed data are provided in Supplementary Spreadsheet S4, Sheet 9 and S5, Sheet 4.

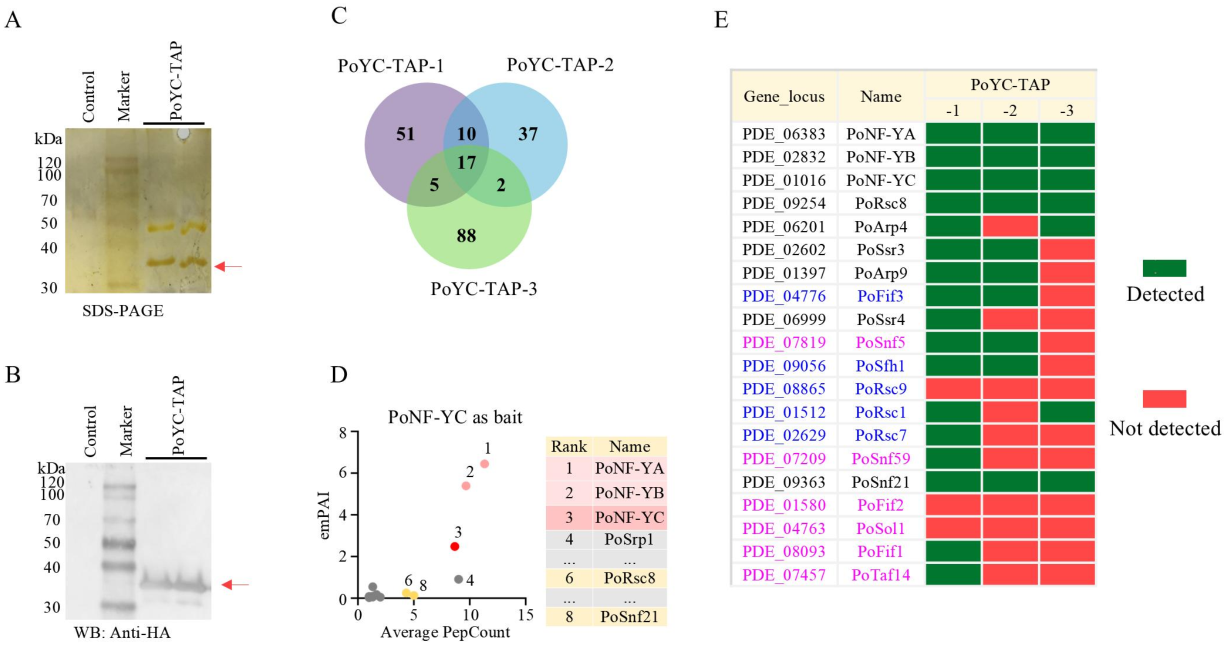

Fig. S8. Results of TAP-MS analysis using the PoNF-YC subunit as bait in *P. oxalicum*. (A, B) SDS-PAGE and Western blot of the TAP eluate. Red arrows indicate the bait protein. (C) Venn diagram showing the number of proteins identified in three biological replicates PoYC-TAP-1/-2/-3. (D) emPAI values versus average peptide counts for the 17 proteins common to all three replicates. Rank, ranked by emPAI value from highest to lowest. Dark red dots/backgrounds, the bait proteins. Light red dots/backgrounds, the two non-bait subunits of the NF-Y complex. Yellow dots/backgrounds, the shared subunits between SWI/SNF and RSC. (E) TAP-MS results from triplicate samples of PoYC-TAP. Black font: the shared subunits between SWI/SNF and RSC. Magenta font: SWI/SNF-specific subunits. Blue font: RSC-specific subunits. Undetected proteins are marked in red. Not all ranking positions are displayed in the figure. The emPAI values for all samples and complete ranked lists are provided in Supplementary Spreadsheets S4, Sheet 1.

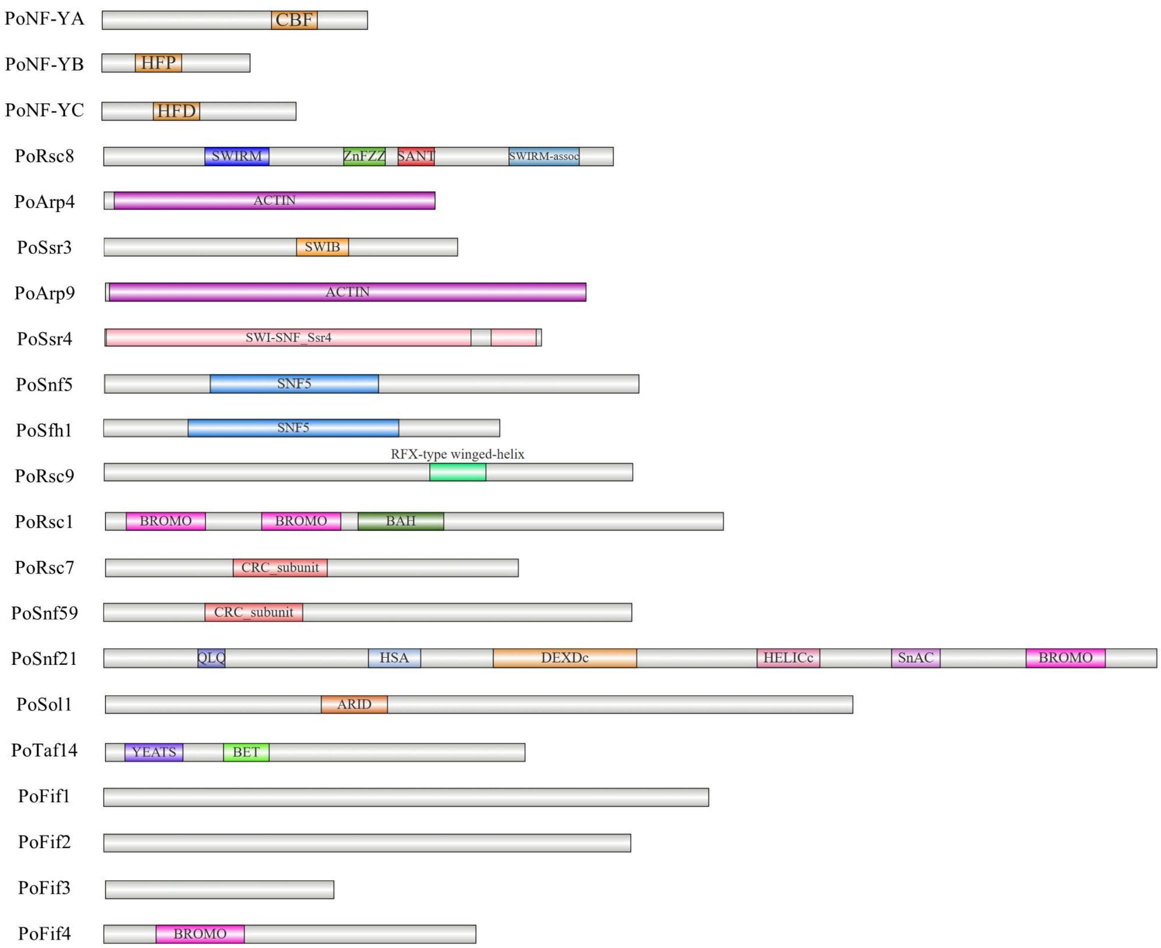

Fig. S9. Domain architectures of subunits from NF-Y, SWI/SNF, and RSC complexes. The analysis was performed using the SMART (http://smart.embl-heidelberg.de/) and Pfam (http://pfam.xfam.org/) databases. Schematic maps were constructed with proportional scaling of respective protein sequences.

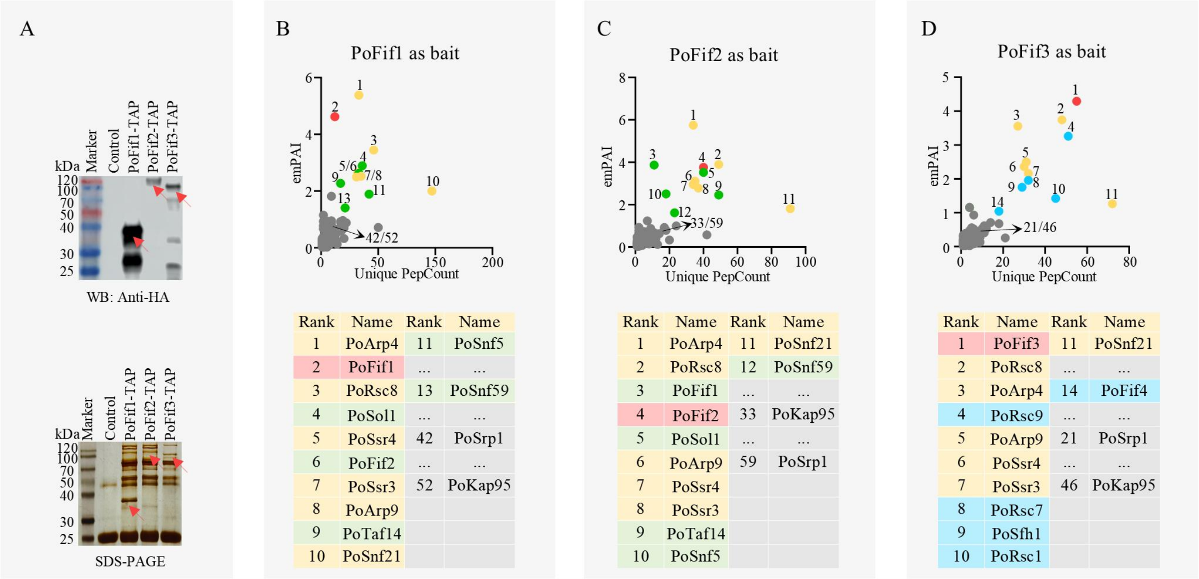

B

Rank	Name	Rank	Name
1	PoArp4	11	PoSnf5
2	PoFif1	...	...
3	PoRsc8	13	PoSnf59
4	PoSol1	...	...
5	PoSsr4	42	PoSrp1
6	PoFif2	...	...
7	PoSsr3	52	PoKap95
8	PoArp9		
9	PoTaf14		
10	PoSnf21		

C

Rank	Name	Rank	Name
1	PoArp4	11	PoSnf21
2	PoRsc8	12	PoSnf59
3	PoFif1	...	...
4	PoFif2	33	PoKap95
5	PoSol1	...	...
6	PoArp9	59	PoSrp1
7	PoSsr4		
8	PoSsr3		
9	PoTaf14		
10	PoSnf5		

D

Rank	Name	Rank	Name
1	PoFif3	11	PoSnf21
2	PoRsc8	...	...
3	PoArp4	14	PoFif4
4	PoRsc9	...	...
5	PoArp9	21	PoSrp1
6	PoSsr4	...	...
7	PoSsr3	46	PoKap95
8	PoRsc7		
9	PoSfh1		
10	PoRsc1		

Fig. S10. Results of TAP-MS using PoFif1, PoFif2, and PoFif3 as baits under high-salt conditions (500 mM NaCl). (A) Western blot (top) and SDS-PAGE (bottom) of the eluates. Red arrows indicate the bait proteins. The theoretical molecular weights of the three baits are 33.1, 77.8, and 92.2 kDa, respectively. (B–D) Proteins identified using PoFif1 (B), PoFif2 (C), and PoFif3 (D) as bait, respectively. Red dots/ backgrounds, the bait proteins. Yellow dots/backgrounds, the shared subunits between SWI/SNF and RSC. Green dots/backgrounds, SWI/SNF-specific subunits. Blue dots/backgrounds, RSC-specific subunits. Grey dots/backgrounds, non-NF-Y or non-CRCs proteins including Importin PoSrp1 (Importin α) and/or PoKap95 (Importin β). Proteins are ranked by emPAI value (highest to lowest). Not all ranking positions are displayed in the figure. The emPAI values for all samples and complete ranked lists are provided in Supplementary Spreadsheets S7.

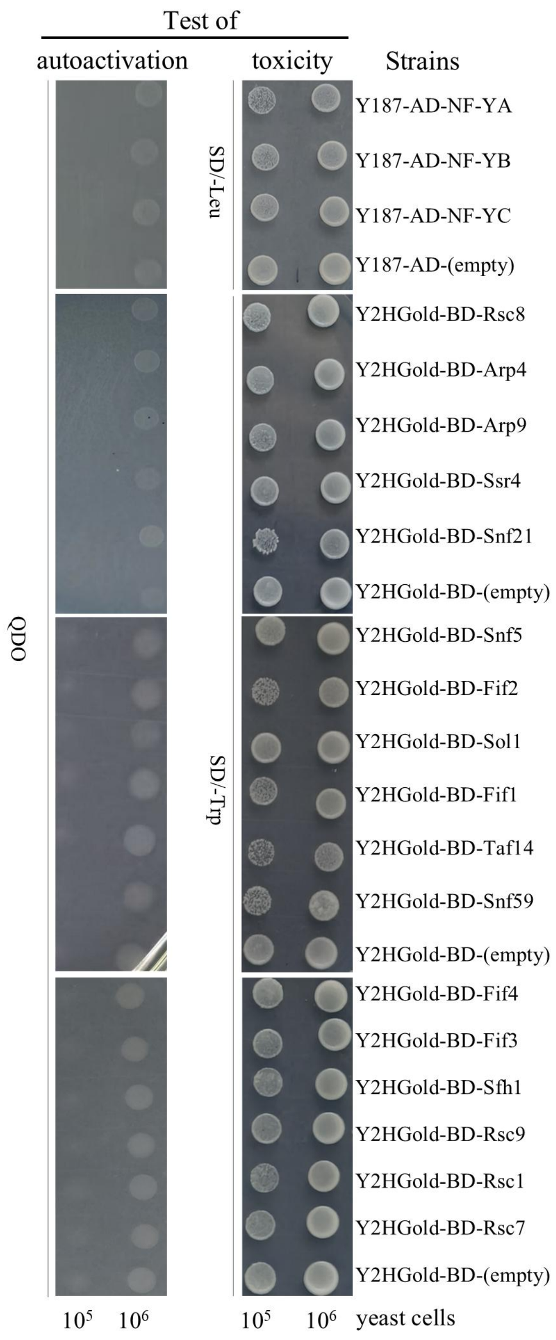

Fig. S11. Test of toxicity and autoactivation of yeast strains for Y2H. Left, autoactivation test. Right, toxicity test.QDO, quadruple-dropout, SD-Ade/-His/-Leu/-Trp.

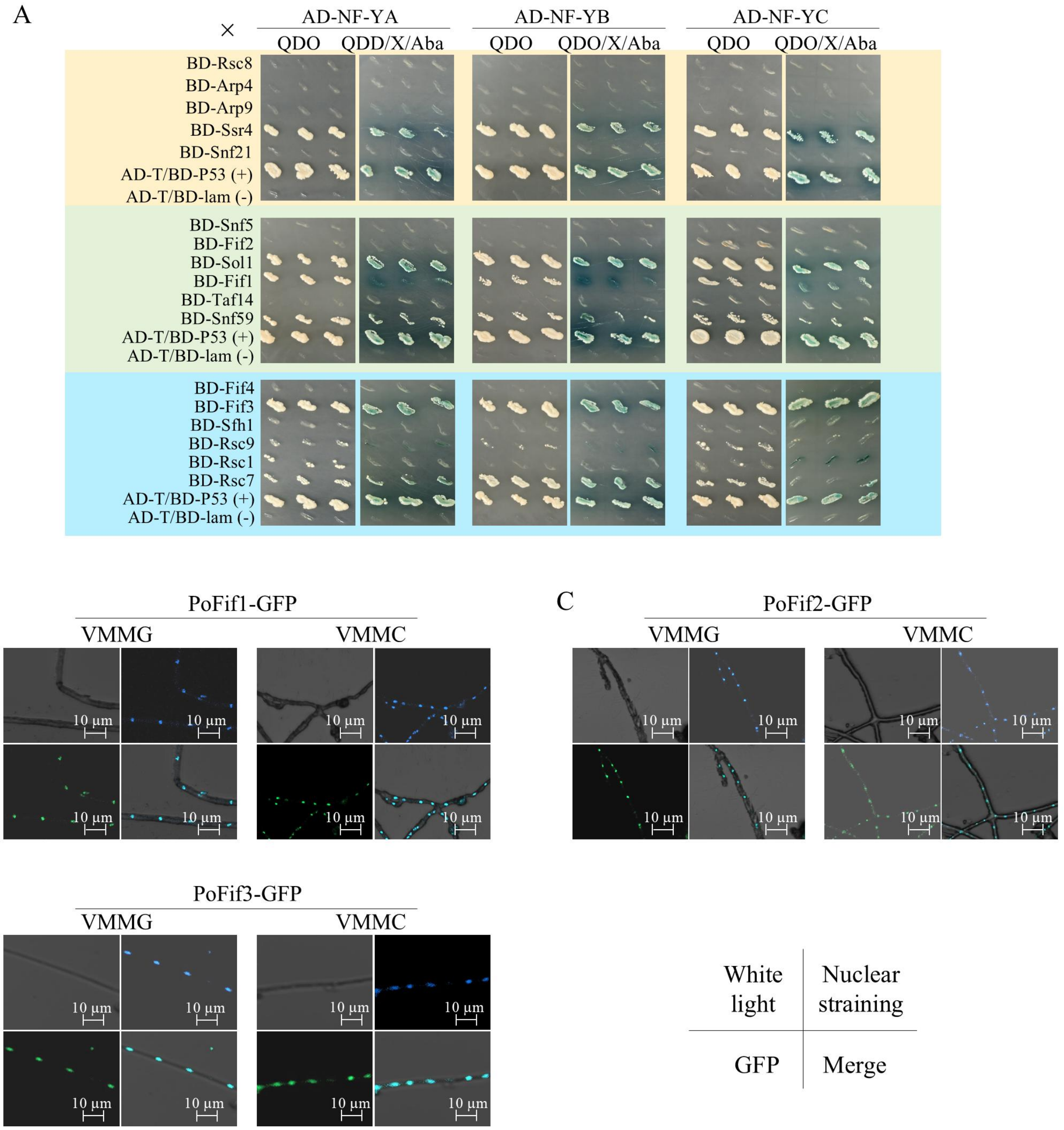

Fig. S12. The results of Y2H and nuclear localization. (A) The results of Y2H using subunits of PoNF-Y, PoSWI/SNF, and PoRSC. BD: the plasmid pGBKT7 containing the binding domain. AD: the plasmid pGADT7 containing the activating domain. QDO: quadruple-dropout, SD-Ade/-His/-Leu/-Trp. QDO/X/Aba: QDO supplemented with X-α-gal and aureobasidin A. Yellow backgrounds, shared units between SWI/SNF and RSC. Green backgrounds, SWI/SNF-specific subunits. Blue backgrounds, RSC-specific subunits. (+): positive control; (-): negative control. **(B–D)** Nuclear localization of PoFif1-Fif3 in glucose (VMMG, left) and cellulose (VMMC, right) conditions, respectively. Each image is divided into four parts: upper left, white light; upper right, Hoechst 33342 staining (nuclei, blue); bottom left, green fluorescence; bottom right, merged image of green fluorescence and nuclear staining.

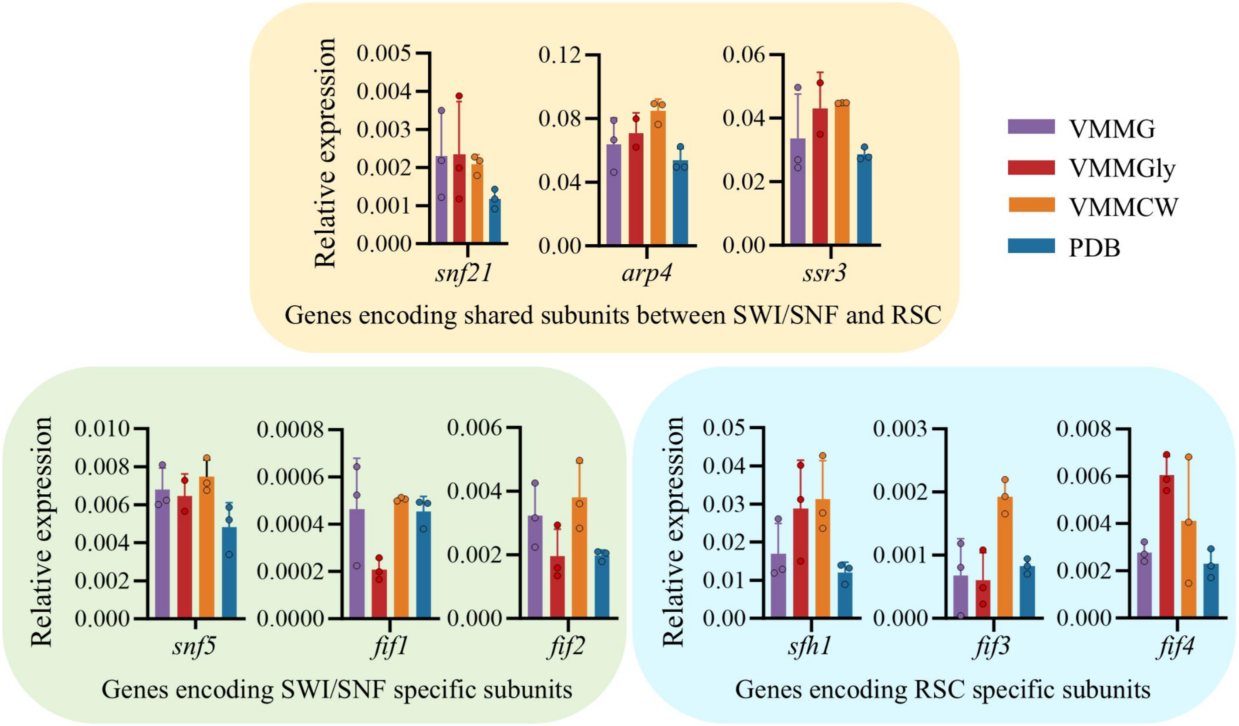

Fig. S13. Transcription level of the CRC subunit gene assayed by qRT-PCR. Yellow backgrounds: the shared subunits between SWI/SNF and RSC. Green ackgrounds: SWI/SNF-specific subunits. Blue backgrounds: RSC-specific subunits. The values were normalized to the actin gene levels (set to 1).

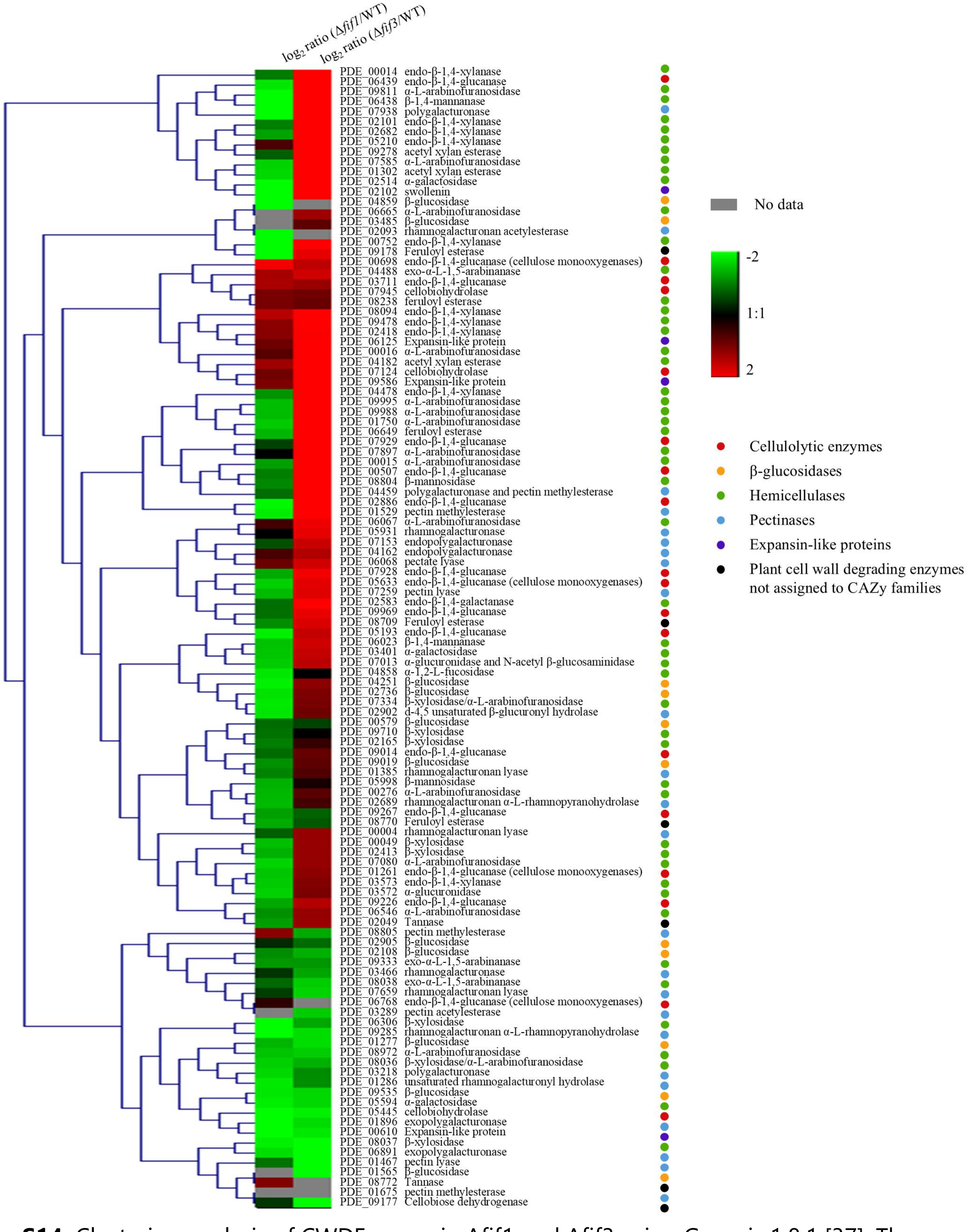

Fig. S14. Clustering analysis of CWDE genes in Δfif1 and Δfif3 using Genesis 1.8.1 [37]. The gradient color bar represents $\log_2$ (FoldChange) expression values in mutants *vs*. the WT strain. FoldChange, the ratio of the mean FPKM of three biological replicates from each mutant to the mean FPKM of three biological replicates from the WT strain.

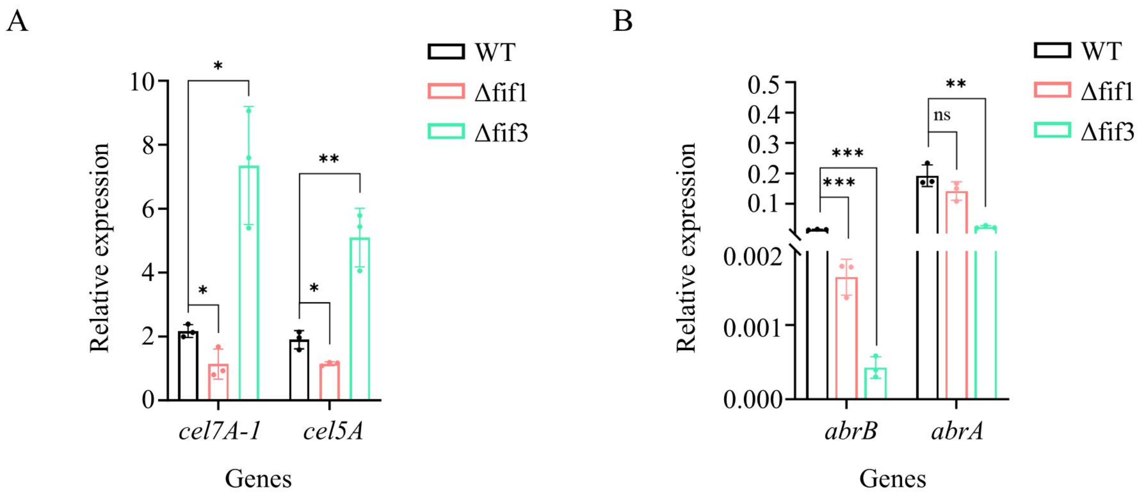

Fig. S15. Transcript levels of two cellulose-degrading genes (A) and two development-related regulatory genes (B) determined by qRT-PCR.

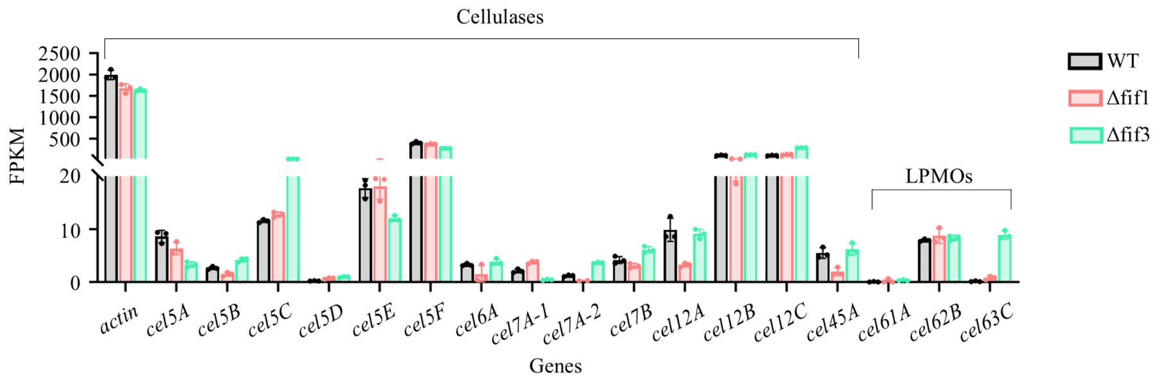

Fig. S16. Cellulose degradation-related gene expression patterns in Δfif1 and Δfif3 mutants in response to glucose conditions.

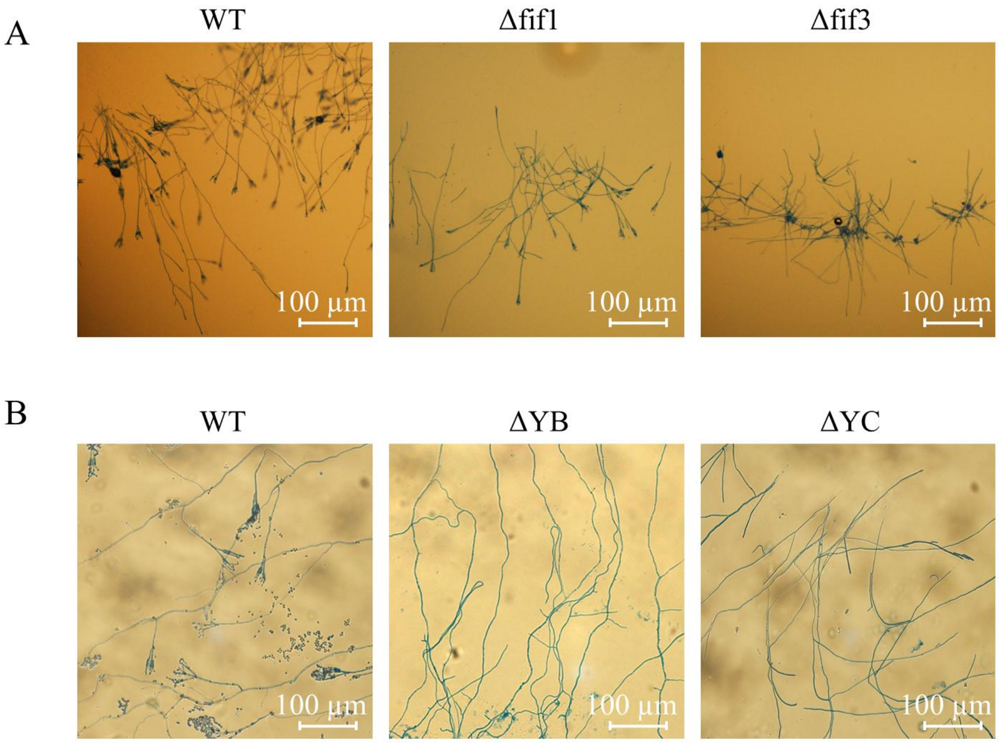

Fig. S17. Conidiophore observation of WT, Δfif1, and Δfif3 (A) and ΔYB and ΔYC (B) at 22 hours post-inoculation.

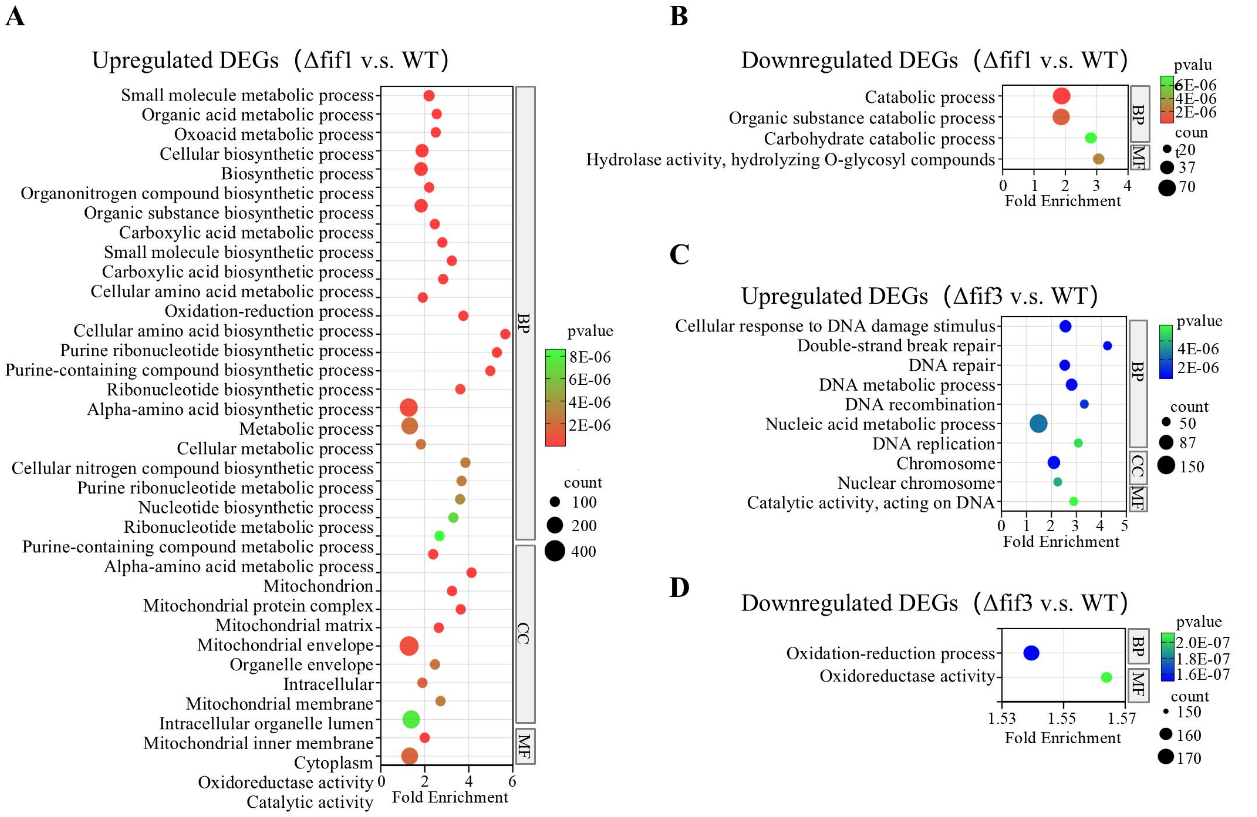

Fig. S18. GO enrichment of in Δfif1 and Δfif3 under glucose signal. GO enrichment of upregulated DEGs (A) and downregulated DEGs (B) in Δfif1. GO enrichment of upregulated DEGs (C) and downregulated DEGs (D) in Δfif3. The enrichment analysis was conducted in ShinyGO, v.0.82 (https://bioinformatics.sdstate.edu/go/). BP, biological process; CC, cellular component; MF, molecular function.

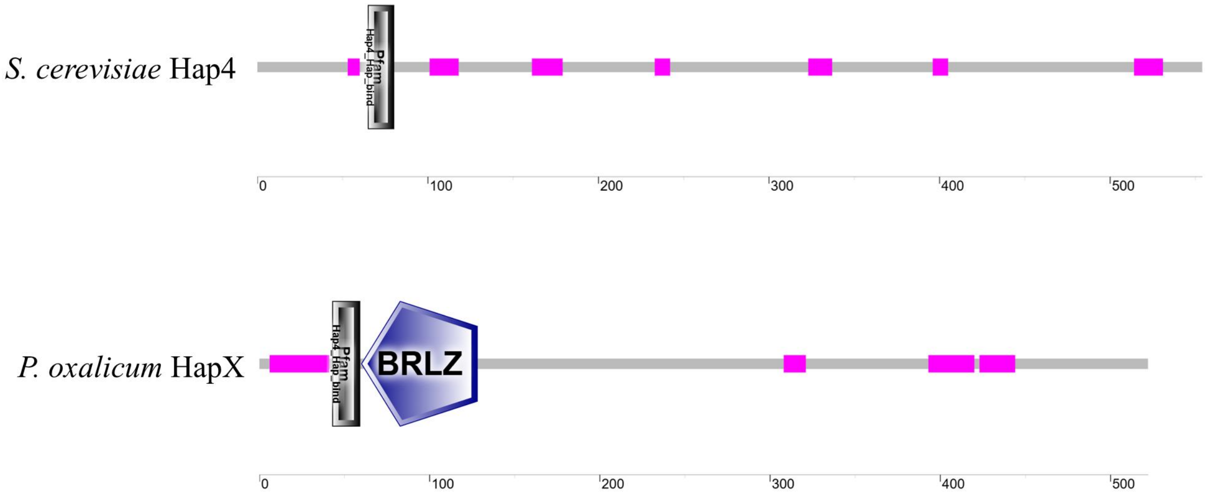

Fig. S19. Domain architectures of *S. cerevisiae* Hap4 and *P. oxalicum* HapX. The analysis was performed using the SMART (http://smart.embl-heidelberg.de/). Hap4_Hap_bind: minimal binding motif of Hap4 for binding to Hap2/3/5. BRLZ: basic region leucin zipper.

Captions of Supplementary datasets

Spreadsheet S1. Strains used in this study.

Spreadsheet S2. The gene names, corresponding loci, and UniProt entries of the proteins involved in this study.

Spreadsheet S3. Primers used in this study.

Spreadsheet S4. Proteins identified by TAP-MS using *P. oxalicum* PoNF-YA (Sheet 2), PoNF-YB (Sheet 3), PoNF-YC (Sheet 1 and 4), PoSnf21 (Sheet 5), PoFif1 (Sheet 6), PoFif2 (Sheet 7), and PoFif3 (Sheet 8) as baits. The proteins identified in the control *P. oxalicum* 114-2 are shown in Sheet 8. For each sample, peptide counts (PepCount), emPAI values, and protein descriptions are presented, with proteins ranked by descending emPAI value. Dark red backgrounds, the bait proteins. Light red backgrounds, the two non-bait subunits of the NF-Y complex. Yellow backgrounds, the shared subunits between SWI/SNF and RSC. Green backgrounds, SWI/SNF-specific subunits. Blue backgrounds, RSC-specific subunits.

Spreadsheet S5. Proteins identified by TAP-MS using *T. reesei* TrNF-YC (Sheet 1), TrFif1 (Sheet 2), and TrFif3 (Sheet 3) as baits. The proteins identified in the control *T. reesei* QMP are shown in Sheet 4. For each sample, peptide counts (PepCount), emPAI values, and protein descriptions are presented, with proteins ranked by descending emPAI value. Dark red backgrounds, the bait proteins. Light red backgrounds, the two non-bait subunits of the NF-Y complex. Yellow backgrounds, the shared subunits between SWI/SNF and RSC. Green backgrounds, SWI/SNF-specific subunits. Blue backgrounds, RSC-specific subunits.

Spreadsheet S6. BLASTP results of PoFif1, PoFif2, PoFif3, and PoFif4 queries against representative filamentous fungi from Eurotiomycetes, Dothideomycetes, Leotiomycetes, and Sordariomycetes.

Spreadsheet S7. Proteins identified by TAP-MS under high-salt conditions using PoFif1 (Sheet 1), PoFif2 (Sheet 2), and PoFif3 (Sheet 3) as baits. For each sample, peptide counts

(PepCount), emPAI values, and protein descriptions are presented, with proteins ranked by descending emPAI value. Dark red backgrounds, the bait proteins. Light red backgrounds, the two non-bait subunits of the NF-Y complex. Yellow backgrounds, the shared subunits between SWI/SNF and RSC. Green backgrounds, SWI/SNF-specific subunits. Blue backgrounds, RSC-specific subunits.

Spreadsheet S8. List of *P. oxalicum* SWI/SNF and RSC subunits and their orthologs in *S. cerevisiae*, *S. pombe*, and *H. sapiens*, identified by sequence homology.

Spreadsheet S9. Prediction scores for Fif1–Fif4 and their putative interacting partners. The prediction was performed using AlphaFold Multimer (https://cosmic-cryoem.org/tools/alphafoldmultimer/). ipTM, interface predicted Template Modeling score. pTM, predicted Template Modeling score.

Spreadsheet S10. Lists of differentially expressed genes (DEGs) ($|\log_2(\text{FoldChange})| \geq 1$, Q-value < 0.05) in Δfif1 vs. WT and Δfif3 vs. WT under lignocellulosic conditions.

Spreadsheet S11. Lists of differentially expressed genes (DEGs) ($|\log_2(\text{FoldChange})| \geq 1$, Q-value < 0.05) in Δfif1 vs. WT and Δfif3 vs. WT under glucose conditions.

Spreadsheet S12. Lists of genes containing a CCAAT box within -200 bp in promoters. Sheet 1, All such genes in the WT strain. Sheets 2 and 3: DGEs containing a CCAAT box in the Δfif1 mutant under cellulose and glucose conditions, respectively. Sheets 4 and 5: DGEs containing a CCAAT box in the Δfif3 mutant under cellulose and glucose conditions, respectively.
